# Supplementary material for: A cluster-randomized trial of water, sanitation, handwashing and nutritional interventions on stress and epigenetic programming
Source: Nat Commun. 2024 Apr 26;15:3572. doi: 10.1038/s41467-024-47896-z (PMC11053067; doi:10.1038/s41467-024-47896-z)
Supplement: Supplementary file 1 — Supplementary Information [file 41467_2024_47896_MOESM1_ESM.pdf]

SUPPLEMENTARY INFORMATION

Table of Contents

CONSORT Checklist 2

Figures

Supplementary Figure 1. Unadjusted means and 95% confidence intervals by the Control arm and Nutrition + WSH arms for all stress outcomes 4

Supplementary Figure 2. Pre- and post-stressor cortisol concentrations by the Control arm and Nutrition + WSH arm 5

Tables

Supplementary Table 1. Enrollment characteristics by intervention group within the WASH Benefits main trial study population, within the stress study population enrolled in Year 1, and within the stress study population lost to follow-up at Year 2 6

Supplementary Table 2. Effect of nutrition, water, sanitation, and handwashing intervention on oxidative stress measurements on Bangladeshi children at age 14 months 7

Supplementary Table 3. Effect of nutrition, water, sanitation, and handwashing intervention on stress response and DNA methylation measurements on Bangladeshi children at age 28 months 8

Supplementary Table 4. Effect modification with child sex on oxidative stress at age 14 months........ 9

Supplementary Table 5. Effect modification with child sex on stress response and DNA methylation at age 28 months........ 10

Supplementary Note

Trial protocol 11

CONSORT Checklist

Item	Description	Reported in Section
**Title and Abstract**		
1a	Identification as a randomized trial in the title; Identification as a cluster randomized trial in the title	Title
1b	Structured summary of trial design, methods, results, and conclusions	Abstract
**Introduction**		
Background and Objectives		
2a	Scientific background and explanation of rationale; Rationale for using a cluster design	Main
2b	Specific objectives or hypotheses; Whether objectives pertain to the cluster level, the individual participant level, or both	Abstract; Main
**Methods**		
Trial Design		
3a	Description of trial design (such as parallel, factorial) including allocation ratio; Definition of cluster and description of how the design features apply to the clusters	Methods
3b	Important changes to methods after trial commencement (such as eligibility criteria), with reasons	N/A
Participants		
4a	Eligibility criteria for participants; Eligibility criteria for clusters	Methods (Participants)
4b	Settings and locations where the data were collected	Methods
Interventions		
5	The interventions for each group with sufficient details to allow replication, including how and when they were actually administered; Whether interventions pertain to the cluster level, the individual participant level, or both	Methods (Procedures)
Outcomes		
6a	Completely defined pre-specified primary and secondary outcome measures, including how and when they were assessed; Whether outcome measures pertain to the cluster level, the individual participant level, or both	Methods (Outcomes)
6b	Any changes to trial outcomes after the trial commenced, with reasons	N/A
Sample Size		
7a	How sample size was determined; Method of calculation, number of cluster(s) (and whether equal or unequal cluster sizes are assumed), cluster size, a coefficient of intracluster correlation (ICC or k), and an indication of its uncertainty	Methods
7b	When applicable, explanation of any interim analyses and stopping guidelines	N/A
**Randomization**		
Sequence Generation		
8a	Method used to generate the random allocation sequence	Methods (Study design and randomization)
8b	Type of randomization; details of any restriction (such as blocking and block size); Details of stratification or matching if used	Methods (Study design and randomization)
Allocation Concealment Mechanism		
9	Mechanism used to implement the random allocation sequence (such as sequentially numbered containers), describing any steps taken to conceal the sequence until interventions were assigned; Specification that allocation was based on clusters rather than individuals and whether allocation concealment (if any) was at the cluster level, the individual participant level, or both	Methods (Study design and randomization)
Implementation		
10a	Who generated the random allocation sequence, who enrolled clusters, and who assigned clusters to interventions	Methods (Study design and randomization)
10b	Mechanism by which individual participants were included in clusters for the purposes of the trial (such as complete enumeration, random sampling)	Methods (Study design and randomization)
10c	From whom consent was sought (representatives of the cluster, or individual cluster members, or both) and whether consent was sought before or after randomization	Methods (Ethics)
Blinding		
11a	If done, who was blinded after assignment to interventions (for example, participants, care providers, those assessing outcomes) and how	Methods (Study design and randomization)
11b	If relevant, description of the similarity of interventions	N/A

Item	Description	Reported in Section
Statistical Methods		
12a	Statistical methods used to compare groups for primary and secondary outcomes; How clustering was taken into account	Methods (Statistical analysis)
12b	Methods for additional analyses, such as subgroup analyses and adjusted analyses	Methods (Statistical analysis)
**Results**		
Participant Flow		
13a	For each group, the numbers of participants/clusters who were randomly assigned, received intended treatment, and were analyzed for the primary outcome	Results
13b	For each group, losses and exclusions after randomization, together with reasons, for both clusters and individual cluster members	Methods (Participants)
Recruitment		
14a	Dates defining the periods of recruitment and follow-up	N/A
14b	Why the trial ended or was stopped	N/A
Baseline Data		
15	A table showing baseline demographic and clinical characteristics for each group; Baseline characteristics for the individual and cluster levels as applicable for each group	Table 1; Supplementary Table 1
Numbers Analyzed		
16	For each group, number of participants/clusters (denominator) included in each analysis and whether the analysis was by the original assigned groups	Results; Table 1
Outcomes and Estimation		
17a	For each primary and secondary outcome, results for each group, and the estimated effect size and its precision (such as 95% confidence interval); Results at the individual and cluster levels as applicable and a coefficient of intracluster correlation (ICC or k) for each primary outcome	Results; Tables 2 and 3; Supplementary Tables 2-3
17b	For binary outcome, presentation of both absolute and relative effect sizes is recommended	N/A
Ancillary Analyses		
18	Results of any other analyses performed, including subgroup analyses and adjusted analyses, distinguishing pre-specified from exploratory	Supplementary Tables 4-5
Harms		
19	All important harms or unintended effects in each group	N/A
**Discussion**		
Limitations		
20	Trial limitations, addressing sources of potential bias, imprecision and, if relevant, multiplicity of analyses	Discussion
Generalizability		
21	Generalizability (external validity, applicability) of the trial findings; Generalizability to clusters and/or individual participants (as relevant)	Discussion
Interpretation		
22	Interpretation consistent with results, balancing benefits and harms, and considering other relevant evidence	Abstract; Discussion
**Other Information**		
Registration		
23	Registration number and name of trial registry	Methods (Statistical analysis)
Protocol		
24	Where the full trial protocol can be accessed, if available	Methods (Statistical analysis)
Funding		
25	Sources of funding and other support (such as supply of drugs), role of funders	Funding

Supplementary Figure 1. Unadjusted means and 95% confidence intervals by the Control arm and Nutrition + WSH arms for all stress outcomes

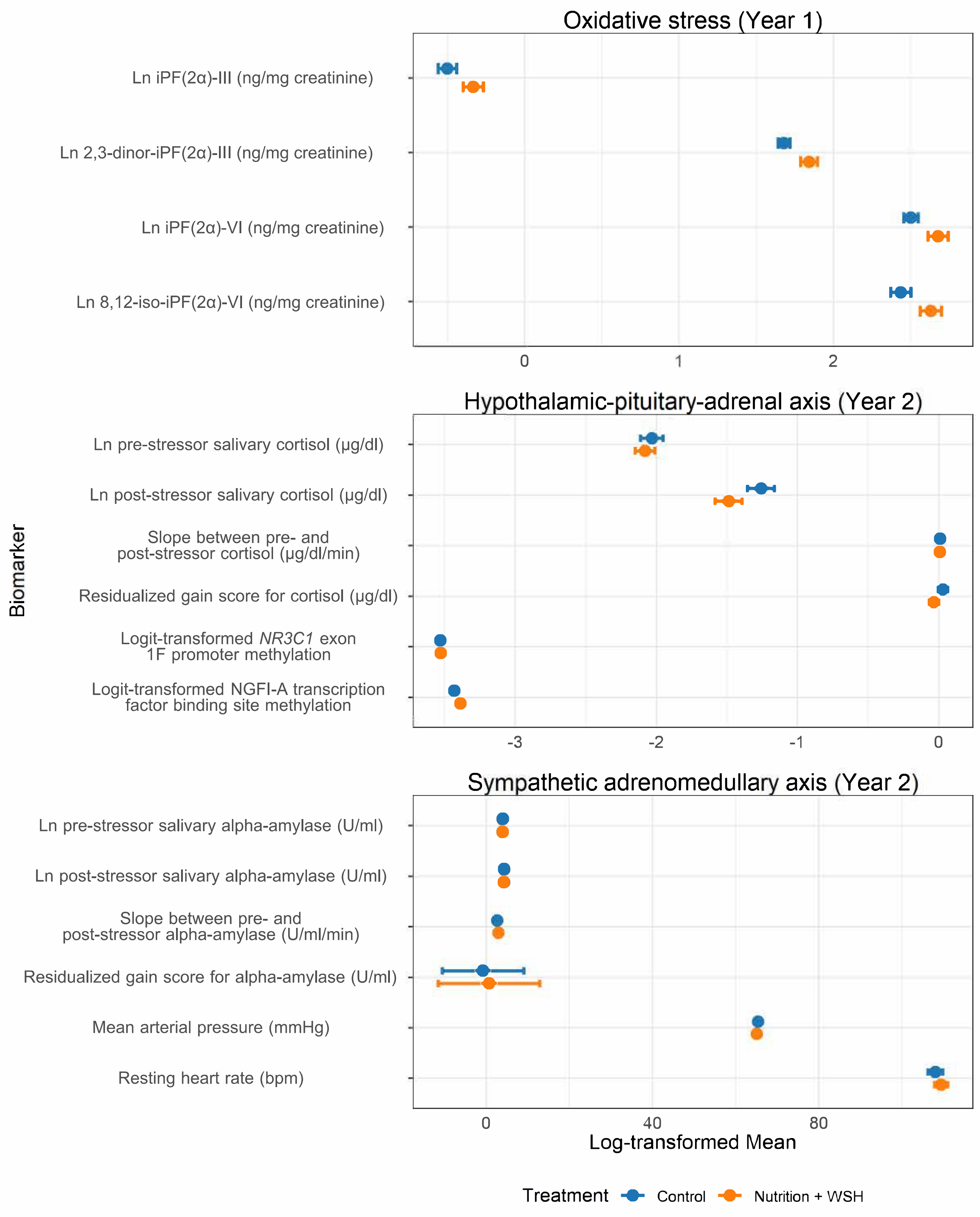

Source data are provided as a Source Data file.

Supplementary Figure 2. Pre- and post-stressor cortisol concentrations by the Control arm and Nutrition + WSH arm

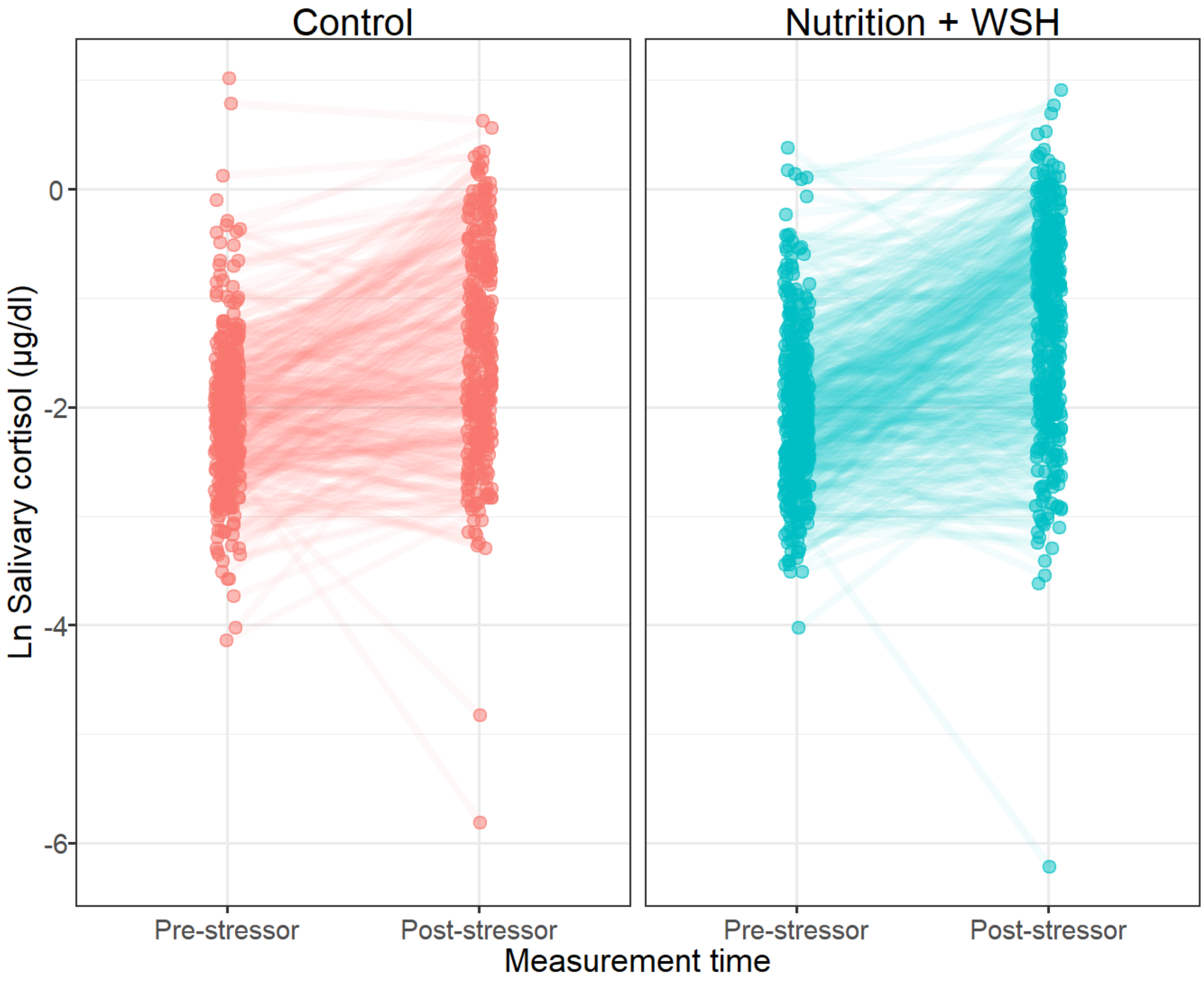

Source data are provided as a Source Data file.

Supplementary Table 1. Enrollment characteristics by intervention group within the WASH Benefits main trial study population, within the stress study population enrolled in Year 1, and within the stress study population lost to follow-up at Year 2

	WASH Benefits Main Trial		Stress Status Study: Had outcomes at Year 1		Stress Status Study: Lost to follow-up at Year 2	
No. of compounds:	Control (N=1382)	N + WSH Intervention (N=686)	Control (N=332)	N+WSH Intervention (N=356)	Control (N=45)	N + WSH Intervention (N=38)
**Maternal**						
Age(years)	24 (5)	24 (6)	23 (5)	24 (6)	23 (5)	24 (6)
Years of education	6 (3)	6 (3)	7 (3)	6 (3)	7 (3)	5 (4)
**Paternal**						
Years of education	5 (4)	5 (4)	6 (4)	5 (4)	6 (3)	5 (4)
Works in agriculture	414 (30%)	207 (30%)	80 (24%)	101 (28%)	10 (22%)	9 (24%)
**Household**						
Number of people	5 (2)	5 (2)	5 (3)	5 (2)	5 (3)	5 (2)
Has electricity	784 (57%)	412 (60%)	200 (60%)	214 (60%)	27 (60%)	20 (53%)
Has a cement floor	145 (10%)	72 (10%)	59 (18%)	43 (12%)	8 (18%)	2 (5%)
Acres of agricultural land owned	0.15 (0.21)	0.14 (0.38)	0.18 (0.25)	0.13 (0.13)	0.18 (0.25)	0.09 (0.09)
**Drinking Water**						
Shallow tubewell primary water source	1038 (75%)	504 (73%)	236 (71%)	249 (70%)	31 (69%)	24 (63%)
Stored water observed at home	666 (48%)	331 (48%)	168 (51%)	185 (52%)	27 (60%)	18 (47%)
Reported treating water yesterday	4 (0%)	2 (0%)	1 (0%)	1 (0%)	0 (0%)	0 (0%)
Distance (mins) to primary water source	1 (3)	1 (2)	1 (2)	1 (2)	1 (1)	1 (1)
**Sanitation**						
Reported daily open defecation						
Adult men	97 (7%)	50 (7%)	12 (4%)	31 (9%)	0 (0%)	3 (8%)
Adult women	62 (4%)	24 (4%)	8 (2%)	16 (5%)	0 (0%)	1 (3%)
Children: 8 to <15 years	53 (10%)	28 (10%)	6 (4%)	18 (11%)	2 (10%)	3 (20%)
Children: 3 to <8 years	267 (38%)	134 (37%)	47 (29%)	68 (33%)	8 (42%)	7 (28%)
Children: 0 to <3 years[a]	245 (82%)	123 (88%)	55 (71%)	69 (88%)	6 (67%)	6 (86%)
Latrine						
Owned[b]	750 (54%)	367 (53%)	208 (63%)	191 (54%)	33 (73%)	18 (47%)
Concrete Slab	1251 (95%)	621 (94%)	315 (97%)	317 (93%)	45 (100%)	37 (100%)
Functional water seal	358 (31%)	155 (27%)	118 (39%)	89 (32%)	21 (48%)	11 (35%)
Visible stool on slab or floor	625 (48%)	298 (46%)	149 (46%)	177 (53%)	17 (38%)	20 (54%)
Owned a child potty	61 (4%)	30 (4%)	27 (8%)	16 (4%)	9 (20%)	0 (0%)
Human feces observed in the						
House	114 (8%)	49 (8%)	23 (7%)	30 (8%)	5 (11%)	2 (5%)
Child's play area	21 (2%)	7 (1%)	4 (1%)	6 (2%)	1 (2%)	0 (0%)
**Handwashing location**						
Within six steps of latrine						
Has water	178 (14%)	72 (11%)	57 (19%)	45 (14%)	5 (12%)	2 (6%)
Has soap	88 (7%)	36 (6%)	30 (10%)	20 (6%)	4 (10%)	2 (6%)
Within six steps of kitchen						
Has water	118 (9%)	60 (9%)	32 (10%)	33 (10%)	2 (5%)	2 (6%)
Has soap	33 (3%)	18 (3%)	12 (4%)	9 (3%)	0 (0%)	0 (0%)
**Nutrition**						
Household is food secure[c]	932 (67%)	485 (71%)	246 (74%)	251 (71%)	34 (76%)	25 (66%)

Data are n (%) or mean (SD). Percentages were estimated from slightly smaller denominators than those shown at the top of the table for the following variables due to missing values: mother's age, father's education, father works in agriculture, acres of land owned, open defecation, latrine has a concrete slab, latrine has a functional water seal, visible stool on latrine slab or floor, ownership of child potty, observed feces in the house or child's play area, handwashing variables.

[a]Open defecation does not include diaper disposal of feces.

[b]Households who do not own a latrine typically share a latrine with extended family members who live in the same compound.

[c]Assessed by the Household Food Insecurity Access Scale.

Source data are provided as a Source Data file.

Supplementary Table 2. Effect of nutrition, water, sanitation, and handwashing intervention on oxidative stress measurements on Bangladeshi children at age 14 months

Urinary F2-isoprostanes	N	Absolute Mean	Mean	SD	Unadjusted difference: Intervention v. Control		Age- and sex-adjusted difference: Intervention v. Control		Fully adjusted difference: Intervention v. Control[a]		IPCW adjusted difference: Intervention v. Control[b]	
					95% CI	P-value	95% CI	P-value	95% CI	P-value	95% CI	P-value
**Ln iPF(2α)-III (ng/mg creatinine)**												
Control	332	0.83	-0.33	0.54								
Nutrition + WSH	356	0.7	-0.5	0.56	-0.16 (-0.27, -0.06)	0.002	-0.17 (-0.28, -0.06)	0.002	-0.14 (-0.22, -0.05)	0.001	-0.14 (-0.17, -0.11)	<0.001
**Ln 2,3-dinor-iPF(2α)-III (ng/mg creatinine)**												
Control	332	6.66	1.84	0.33								
Nutrition + WSH	356	5.64	1.68	0.32	-0.16 (-0.23, -0.09)	<0.001	-0.19 (-0.26, -0.13)	<0.001	-0.18 (-0.24, -0.12)	<0.001	-0.22 (-0.24, -0.2)	<0.001
**Ln iPF(2α)-VI (ng/mg creatinine)**												
Control	332	16.36	2.68	0.46								
Nutrition + WSH	356	13.5	2.5	0.44	-0.17 (-0.25, -0.1)	<0.001	-0.2 (-0.27, -0.13)	<0.001	-0.19 (-0.25, -0.12)	<0.001	-0.11 (-0.14, -0.08)	<0.001
**Ln 8,12-iso-iPF(2α)-VI (ng/mg creatinine)**												
Control	332	16.05	2.63	0.55								
Nutrition + WSH	356	13.49	2.44	0.57	-0.19 (-0.29, -0.1)	<0.001	-0.21 (-0.31, -0.12)	<0.001	-0.24 (-0.32, -0.16)	<0.001	-0.18 (-0.22, -0.15)	<0.001

We used targeted maximum likelihood estimation with influence curve-based standard errors accounting for clustered observations from the trial's geographic block-randomized design. Confidence intervals were adjusted for clustered observations using robust standard errors. The statistical tests were two-sided, and no adjustments were made for multiple comparisons.

[a]Adjusted for prespecified covariates: child sex, child birth order, mother's age, mother's height, mother's education, number of children <18 years in the household, number of individuals living in the compound, distance in minutes to the primary water source, household food security, household floor materials, household wall materials, household electricity, and household assets (wardrobe, table, chair, clock, khat, chouki, radio, television, refrigerator, bicycle, motorcycle, sewing machine, mobile phone, cattle, goats, and chickens), child age at dates of urine, vitals, and saliva collection, and monsoon season at dates of urine, vitals, and saliva collection.

[b]Inverse probability of censoring weighting.

Source data are provided as a Source Data file.

Supplementary Table 3. Effect of nutrition, water, sanitation, and handwashing intervention on stress response and DNA methylation measurements on Bangladeshi children at age 28 months

Outcome	N	Absolute Mean	Mean	SD	Unadjusted difference: Intervention v. Control		Age- and sex-adjusted difference: Intervention v. Control		Fully adjusted difference: Intervention v. Control[a]		IPCW adjusted difference: Intervention v. Control[b]	
					95% CI	P-value	95% CI	P-value	95% CI	P-value	95% CI	P-value
**Ln pre-stressor Salivary alpha-amylase (U/ml)**												
Control	354	74.9	4.01	0.81								
Nutrition + WSH	394	75.92	3.98	0.89	-0.03 (-0.19, 0.13)	0.731	-0.02 (-0.18, 0.14)	0.785	-0.02 (-0.17, 0.14)	0.834	-0.01 (-0.15, 0.13)	0.914
**Ln post-stressor Salivary alpha-amylase (U/ml)**												
Control	339	124.06	4.47	0.9								
Nutrition + WSH	375	122.53	4.41	0.99	-0.06 (-0.26, 0.13)	0.530	-0.06 (-0.24, 0.13)	0.550	-0.01 (-0.2, 0.18)	0.928	-0.02 (-0.08, 0.04)	0.470
**Slope between pre- and post-stressor alpha-amylase (U/ml/min)**												
Control	335	2.99	2.99	6.43								
Nutrition + WSH	367	2.73	2.73	5.29	-0.22 (-1.07, 0.64)	0.622	-0.2 (-1.02, 0.62)	0.635	0.09 (-0.72, 0.91)	0.821	0.3 (-0.66, 1.26)	0.542
**Residualized gain score for alpha-amylase (U/ml)**												
Control	335	0.77	0.77	108.21								
Nutrition + WSH	368	-0.7	-0.7	89.31	-1.17 (-15.16, 12.82)	0.870	-0.19 (-13.82, 13.45)	0.979	4.97 (-8.79, 18.72)	0.479	17.77 (12.08, 23.47)	<0.001
**Ln pre-stressor salivary cortisol (µg/dl)**												
Control	357	0.17	-2.08	0.69								
Nutrition + WSH	396	0.18	-2.03	0.73	0.05 (-0.08, 0.18)	0.458	0.04 (-0.09, 0.17)	0.553	0.04 (-0.08, 0.17)	0.487	0.05 (0.01, 0.09)	0.008
**Ln post-stressor salivary cortisol (µg/dl)**												
Control	312	0.34	-1.49	0.96								
Nutrition + WSH	385	0.42	-1.26	0.95	0.24 (0.07, 0.4)	0.005	0.21 (0.05, 0.37)	0.010	0.21 (0.06, 0.36)	0.007	0.2 (0.06, 0.34)	0.005
**Slope between pre- and post-stressor cortisol (µg/dl/min)**												
Control	311	0.01	0.01	0.01								
Nutrition + WSH	380	0.01	0.01	0.01	0.002 (0, 0.003)	0.035	0.002 (0, 0.003)	0.053	0.002 (0, 0.003)	0.031	0.003 (0.001, 0.004)	<0.001
**Residualized gain score for cortisol (µg/dl)**												
Control	311	-0.03	-0.03	0.27								
Nutrition + WSH	380	0.03	0.03	0.32	0.06 (0.01, 0.12)	0.023	0.06 (0, 0.11)	0.033	0.06 (0.01, 0.11)	0.018	0.09 (0.04, 0.15)	0.001
**Mean arterial pressure (mmHg)**												
Control	353	65.18	65.18	6.14								
Nutrition + WSH	399	65.5	65.5	6.78	0.33 (-1, 1.66)	0.625	0.32 (-1.06, 1.7)	0.649	0.31 (-0.84, 1.46)	0.596	0.46 (0.06, 0.86)	0.025
**Resting heart rate (bpm)**												
Control	358	109.49	109.49	14.43								
Nutrition + WSH	398	108.12	108.12	17.12	-1.35 (-4.08, 1.39)	0.334	-1.43 (-4.14, 1.27)	0.299	-1.65 (-4.26, 0.97)	0.218	-1.36 (-2.29, -0.42)	0.005
**Logit-transformed *NR3C1* exon 1F promoter methylation**												
Control	346	0.39	-3.53	0.09								
Nutrition + WSH	396	0.38	-3.53	0.1	-0.001 (-0.02, 0.018)	0.917	-0.001 (-0.021, 0.019)	0.895	-0.003 (-0.02, 0.014)	0.720	-0.01 (-0.02, 0)	0.005
**Logit-transformed NGFI-A transcription factor binding site methylation**												
Control	336	0.9	-3.39	0.25								
Nutrition + WSH	386	0.77	-3.43	0.26	-0.04 (-0.08, 0)	0.037	-0.04 (-0.08, 0)	0.080	-0.04 (-0.08, 0)	0.037	-0.05 (-0.09, -0.01)	0.017

We used targeted maximum likelihood estimation with influence curve-based standard errors accounting for clustered observations from the trial's geographic block-randomized design. Confidence intervals were adjusted for clustered observations using robust standard errors. The statistical tests were two-sided, and no adjustments were made for multiple comparisons.

[a]Adjusted for prespecified covariates: child sex, child birth order, mother's age, mother's height, mother's education, number of children <18 years in the household, number of individuals living in the compound, distance in minutes to the primary water source, household food security, household floor materials, household wall materials, household electricity, and household assets (wardrobe, table, chair, clock, khat, chouki, radio, television, refrigerator, bicycle, motorcycle, sewing machine, mobile phone, cattle, goats, and chickens), child age at dates of urine, vitals, and saliva collection, and monsoon season at dates of urine, vitals, and saliva collection. For salivary alpha-amylase and cortisol analyses only, time of sampling was included as a covariate.

[b]Inverse probability of censoring weighting.

Source data are provided as a Source Data file.

Supplementary Table 4. Effect modification with sex at 14 months

	Female					Male					Interaction P-value
Urinary F2-isoprostanes	N	Mean	SD	Unadjusted difference: Intervention vs. Control (95% CI)	P-value	N	Mean	SD	Unadjusted difference: Intervention vs. Control (95% CI)	P-value	
Ln iPF(2α)-III (ng/mg creatinine)	349	0.77	0.42	-0.11 (-0.23, 0)	0.053	339	0.75	0.56	-0.22 (-0.35, -0.1)	<0.001	0.231
Ln 2,3-dinor-iPF(2α)-III (ng/mg creatinine)	349	6.33	2.37	-0.21 (-0.29, -0.13)	<0.001	339	5.94	1.92	-0.12 (-0.19, -0.05)	0.001	0.051
Ln iPF(2α)-VI (ng/mg creatinine)	349	15.3	7.28	-0.16 (-0.26, -0.06)	0.001	339	14.44	11.73	-0.19 (-0.29, -0.09)	<0.001	0.712
Ln 8,12-iso-iPF(2α)-VI (ng/mg creatinine)	349	15.66	9.65	-0.22 (-0.34, -0.09)	<0.001	339	13.77	7.93	-0.18 (-0.3, -0.06)	0.004	0.628

We used targeted maximum likelihood estimation with influence curve-based standard errors accounting for clustered observations from the trial's geographic block-randomized design. Confidence intervals were adjusted for clustered observations using robust standard errors. The statistical tests were two-sided, and no adjustments were made for multiple comparisons.
Source data are provided as a Source Data file.

Supplementary Table 5. Effect modification with sex at 28 months

	Female					Male					Interaction P-value
Outcome	N	Mean	SD	Unadjusted difference: Intervention vs. Control (95% CI)	P-value	N	Mean	SD	Unadjusted difference: Intervention vs. Control (95% CI)	P-value	
Mean arterial pressure (mmHg)	393	65.26	6.45	-0.01 (-1.43, 1.41)	0.993	359	65.45	6.53	0.67 (-0.97, 2.3)	0.427	0.496
Resting heart rate (bpm)	393	110.15	16.24	0.57 (-2.3, 3.44)	0.697	363	107.27	15.42	-3.53 (-6.62, -0.44)	0.025	0.027
Ln pre-stressor alpha-amylase (U/ml)	388	70.87	56.22	-0.03 (-0.2, 0.14)	0.718	360	80.35	68.76	-0.02 (-0.19, 0.15)	0.804	0.920
Ln post-stressor alpha-amylase (U/ml)	371	122.57	126.06	-0.02 (-0.21, 0.17)	0.873	343	124	97.8	-0.11 (-0.33, 0.11)	0.328	0.536
Ln pre-stressor cortisol (µg/dl)	390	0.19	0.24	0.16 (0.01, 0.32)	0.038	363	0.16	0.13	-0.08 (-0.23, 0.08)	0.330	0.035
Ln post-stressor cortisol (µg/dl)	363	0.4	0.38	0.31 (0.14, 0.47)	<0.001	334	0.37	0.31	0.15 (-0.04, 0.34)	0.128	0.188
Logit-transformed *NR3C1* exon 1F promoter methylation	387	0.4	0.35	0 (-0.02, 0.03)	0.708	355	0.36	0.25	-0.01 (-0.03, 0.01)	0.425	0.345
Logit-transformed NGFI-A transcription factor binding site methylation	379	0.82	0.94	-0.02 (-0.07, 0.03)	0.414	343	0.85	0.96	-0.07 (-0.12, -0.01)	0.012	0.166
Slope between pre- and post-stressor alpha-amylase (U/ml/min)	364	3.02	6.65	0.23 (-1.16, 1.63)	0.741	338	2.67	4.87	-0.81 (-2, 0.39)	0.186	0.277
Slope between pre- and post-stressor cortisol (µg/dl/min)	358	0.01	0.01	0 (0, 0)	0.052	333	0.01	0.01	0 (0, 0)	0.079	0.862
Residualized gain score for alpha-amylase (U/ml)	364	3.15	113.43	5.12 (-18.6, 28.84)	0.672	339	-3.38	79.94	-8.7 (-26.76, 9.35)	0.345	0.380
Residualized gain score for cortisol (µg/dl)	358	0	0.32	0.07 (0, 0.13)	0.036	333	0	0.28	0.06 (0, 0.12)	0.063	0.774

We used targeted maximum likelihood estimation with influence curve-based standard errors accounting for clustered observations from the trial's geographic block-randomized design. Confidence intervals were adjusted for clustered observations using robust standard errors. The statistical tests were two-sided, and no adjustments were made for multiple comparisons.
Source data are provided as a Source Data file.

Amendment Application 1

Personnel Information 4

Vulnerable Subject Checklist 7

Study Sites 8

General Checklist 9

Funding 10

Expedited Paragraphs 14

Purpose, Background, Collaborative Research 17

Subject Population 35

Study Procedures, Alternatives to Participation 43

Radiation 71

Medical Equipment, Investigational Devices 72

Drugs, Reagents, or Chemicals 73

Risks and Discomforts 75

Benefits, Confidentiality 78

Potential Financial Conflict of Interest 82

Informed Consent 83

Child Assent & Parent Permission 98

HIPAA 101

Attachments 103

Assurance 109

Event History 111

PROTOCOL
Biomedical
Non-Exempt
Berkeley

Protocol # 2011-09-3652
Date Printed: 10/11/2021

**Protocol Title:**	Measuring the benefits of sanitation, water quality, handwashing and nutrition interventions for improving health and development in rural Bangladesh
**Protocol Status:**	APPROVED
**Date Submitted:**	08/18/2014
**Approval Period:**	**09/16/2014-11/03/2016**
**Important Note:**	**This Print View may not reflect all comments and contingencies for approval. Please check the comments section of the online protocol. Questions that appear to not have been answered may not have been required for this submission. Please see the system application for more details.**

* * * Amendment Application * * *

Amendment Application

1. Summarize the amendment (or proposed changes) you wish to make to your study.

a.We added Mark Davis, Juergen Erhardt, and Jessica Grembi to Section 3a and 4a.
b.We added information about collection of saliva and mothers' urine, and hair to Sections 2, 6a, 9a, 9b.
c.We added details about an anemia assessment to Sections 2, 9a, 15g.
d.We added details about a microbiome assessment to be conducted after endline (Section 9a).
e.We modified section 5a and 5b to reflect changes proposed in this amendment.
f.We updated information about selection of environmental enteropathy cohort members in Section 9a and increased the number of children invited to participate in the EE assessment to 2,000 instead of 1,500 (Sections 5a, 5b, 9a).
g.We refined details about the analysis of urine samples using mass spectrometry (Section 9a).
h.We added details about immunological analyses of blood samples to be conducted at Stanford (Section 9a).
i.We increased the total blood volume we propose to collect from participants (Section 9).
j.We added details about a sustainability assessment to be conducted at endline (Section 9).
k.We added details about analyzing stool samples for soil-transmitted helminths and deworming all children at endline (Section 9a).
l.We added details about chlorine water testing in households reporting that they treated water (Section 9a).
m.We added details about assessment of child development and maternal intelligence at endline (Section 9a).
n.We modified consent forms i, j, and u and added consent forms for the microbiome assessment (1z and 2z) (Section 19).
o.We added a document with updated endline survey modules.
p.We added funding information for the anemia work, which is funded through a subaward from UC Davis.
q.We added the latest IRB approval letter from ICDDR,B (June 2014).
r.We added information about the involvement of a witness during the informed consent process to section 6a.
s.We added information about the reason for deworming participants who provide a stool sample to section 9a.

2. Explain the reason(s) for the proposed amendment(s).

a.These new collaborators will contribute to immunological and microbiome analyses of biological specimens.
b.These activities are part of the EE substudy.
c.The anemia assessment will contribute to the critical evidence gap regarding the causes of anemia and interventions to prevent it.
d.The microbiome assessment will contribute to our understanding of the association between WASH interventions and the development of the gut microbiota.

Protocol Title: Measuring the benefits of sanitation, water quality, handwashing and nutrition interventions for improving health and development in rural Bangladesh

Protocol Status: APPROVED

Date Submitted: 08/18/2014

Approval Period: 09/16/2014-11/03/2016

Important Note: This Print View may not reflect all comments and contingencies for approval. Please check the comments section of the online protocol.
Questions that appear to not have been answered may not have been required for this submission. Please see the system application for more details.

interventions and the development of the gut microbiota.
e.We increased the number of people we will invite to participate in the EE substudy from 1,500 to 2,000 because we have determined we need to do so to reach our desired sample of 1,500 given current response rates. While we have listed the number of children and mothers invited to participate as 2,000 for each group instead of 1,500 this does not impact the total number of study participants because we will invite individuals who are already enrolled in the main trial.
f.See 2e.
g.We made this modification to be consistent with international standards for EE analyses, which are currently under development.
h.These details have been developed since our last amendment.
i.The increase in blood volume reflects the additional anemia activities proposed in this amendment.
j.We will evaluate the extent to which the interventions are in use at the end of the trial and to ask questions which will help them assess whether the interventions will continue to be used after the trial.
k.This activity is part of the parasites assessment.
l.These details were developed since our last protocol amendment.
m.These details were developed since our last protocol amendment.
n.These changes reflect the additional samples we propose to collect as described in Section 9a.
o.These are new instruments to be administered at endline.
p.This award was processed since our last amendment.
q.We received this since our last amendment.
r.The consent forms include a thumb print/signature line for a witness but the rationale for this was previously not described in the protocol.
s.We previously did not discuss our rationale for this.

3. **Indicate how the change(s) impact the level of risk to subjects:**

	**Increase**
Y	**No Change**
	**Decrease**

4. **Describe any effects the change(s) will have regarding risk(s) to the subjects:**

N/A

5. **Will this amendment require the re-consent of any currently enrolled subjects?** N

If YES, please explain.

6. **Is this modification consistent with the scope of research activities as described in the proposal(s) for the grant(s) funding the research? (Check N/A** Y

---

described in the proposal(s) for the grant(s) funding the research? (Check N/A if you have no external funding)

7. **Proceed to the appropriate section(s) and make your changes. The list of sections that have been changed or modified will appear below:**

**Protocol Title:**	Measuring the benefits of sanitation, water quality, handwashing and nutrition interventions for improving health and development in rural Bangladesh
**Protocol Status:**	APPROVED
**Date Submitted:**	08/18/2014
**Approval Period:**	**09/16/2014-11/03/2016**
**Important Note:**	**This Print View may not reflect all comments and contingencies for approval. Please check the comments section of the online protocol. Questions that appear to not have been answered may not have been required for this submission. Please see the system application for more details.**

* * * Personnel Information * * *

Enter all UC Berkeley study personnel (if not previously entered) and relevant training information. Please read Personnel Titles and Responsibilities: Roles in eProtocol before completing this section.

Note: The Principal Investigator or Faculty Sponsor, Co-Principal Investigator, Student or Postdoctoral Investigator, Administrative Contact, and Other Contact can EDIT and SUBMIT. Other Personnel can only VIEW the protocol.

Principal Investigator or Faculty Sponsor

**Name of Principal Investigator**	**Degree (e.g., MS/PhD)**	**Title**
Jack COLFORD		Professor
**Email**	**Phone**	**Fax**
jcolford@berkeley.edu	+1 510 642-9370	+1 510 666-2551
**Department Name**	**Mailing Address**	
Pub Hlth-Epidemiology	94720-7358	

UCB status (select all that apply):

X	Faculty		Postdoc		Grad		Undergrad		Other	

Faculty (with some exceptions), staff, and students engaged in human subjects research must complete either the biomedical or social-behavioral human research course through the online Collaborative Institutional Training Initiative (CITI), depending upon which is most germane to the research. ALL PIs on an NIH award are required to complete either CITI or NIH Training. See Training and Education for more information.

If applicable, please insert date (mm/dd/yy) of completion in appropriate box(es) below:

CITI	NIH	Other Training (title & date completed)

Administrative Contact

**Name of Administrative Contact**	**Degree**	**Title**
Jade D. Benjamin-Chung	MPH	
**Email**	**Phone**	**Fax**

jadebc@berkeley.edu

**Department Name**	**Mailing Address**
Pub Hlth-Epidemiology	

UCB status (select all that apply):

	**Faculty**		**Postdoc**	X	**Grad**		**Undergrad**		**Other**	

Other Contact

**Name of Other Contact**	**Degree**	**Title**
Melanie Beth GENDELL		Administrative Manager
**Email**	**Phone**	**Fax**
mgendell@berkeley.edu	+1 510 643-5742	+1 510 643-7316
**Department Name**	**Mailing Address**	
	94720-7360	

UCB status (select all that apply):

	**Faculty**		**Postdoc**		**Grad**		**Undergrad**		**Other**	

Other Personnel

Name	Degree	Title	Department Name
Benjamin F. Arnold		Epidemiologist	Pub Hlth-Epidemiology
Alan HUBBARD		Associate Professor	Pub Hlth-Biostatistics
Lia C. FERNALD		Associate Professor	Pub Hlth-Nutrition
Audrie Lin	PhD	Specialist	Pub Hlth-Epidemiology
Kara Nelson		Professor	Civ Engr/CEE Environmental
Ayse Ercumen	PhD	Specialist	Pub Hlth-Epidemiology

Protocol Title: Measuring the benefits of sanitation, water quality, handwashing and nutrition interventions for improving health and development in rural Bangladesh

Protocol Status: APPROVED

Date Submitted: 08/18/2014

Approval Period: 09/16/2014-11/03/2016

Important Note: This Print View may not reflect all comments and contingencies for approval. Please check the comments section of the online protocol.
Questions that appear to not have been answered may not have been required for this submission. Please see the system application for more details.

Protocol Title: Measuring the benefits of sanitation, water quality, handwashing and nutrition interventions for improving health and development in rural Bangladesh

Protocol Status: APPROVED

Date Submitted: 08/18/2014

Approval Period: 09/16/2014-11/03/2016

Important Note: **This Print View may not reflect all comments and contingencies for approval. Please check the comments section of the online protocol. Questions that appear to not have been answered may not have been required for this submission. Please see the system application for more details.**

* * * Vulnerable Subject Checklist * * *

Vulnerable Subject Checklist

Yes	No	
Y		Children/Minors
	N	Prisoners
Y		Pregnant Women
	N	Fetuses
Y		Neonates
Y		Educationally Disadvantaged
Y		Economically Disadvantaged
	N	Cognitively Impaired
	N	Other (i.e., any vulnerable subject population(s) not specified above)

**Protocol Title:**	Measuring the benefits of sanitation, water quality, handwashing and nutrition interventions for improving health and development in rural Bangladesh
**Protocol Status:**	APPROVED
**Date Submitted:**	08/18/2014
**Approval Period:**	**09/16/2014-11/03/2016**
**Important Note:**	**This Print View may not reflect all comments and contingencies for approval. Please check the comments section of the online protocol. Questions that appear to not have been answered may not have been required for this submission. Please see the system application for more details.**

* * * Study Sites * * *

Study Sites

Select All That Apply :

International

X International Site(s) (specify country, region, and township or village)

Bangladesh

Local

X UC Berkeley

X UC Davis

UC Irvine

UC Los Angeles

UC Merced

UC Riverside

UC San Diego

UC San Francisco

UC Santa Barbara

UC Santa Cruz

Lawrence Berkeley National Laboratory

Alameda Unified School District (specify schools below)

Berkeley Unified School District (specify schools below)

Oakland Unified School District (specify schools below)

Other (Specify other Study Sites)

**Protocol Title:**	Measuring the benefits of sanitation, water quality, handwashing and nutrition interventions for improving health and development in rural Bangladesh
**Protocol Status:**	APPROVED
**Date Submitted:**	08/18/2014
**Approval Period:**	**09/16/2014-11/03/2016**
**Important Note:**	**This Print View may not reflect all comments and contingencies for approval. Please check the comments section of the online protocol. Questions that appear to not have been answered may not have been required for this submission. Please see the system application for more details.**

* * * General Checklist * * *

General Checklist

Yes	No	
	N	Is the research receiving any federal funding (e.g., NIH, NSF, DOD, etc.)?
	N	Is another campus relying on UC Berkeley for IRB review by means of the UC System Memorandum of Understanding (MOU)?
Y		Is another institution relying on UC Berkeley for IRB review by means of an Inter-institutional IRB Authorization Agreement?
	N	Will subjects be paid for participation?
	N	Is this protocol administratively supported by Research Enterprise Services (RES)?
	N	Does the research constitute a <a href=http://cphs.berkeley.edu/faqs.html#clinicaltrials target=blank > clinical trial?
Y		Any use of human blood, body fluids, tissues, or cells (including cell lines)* by drawing samples, accepting samples already drawn, receiving samples from any source, or in any other way?
		If yes, Lab Location: ICDDRB Bangladesh
		And Biological Use Authorization (BUA) #(s):
Y		Will biological specimens be stored for future research projects?
	N	Will specimens be sent out of UCB as part of a research agreement?
	N	Will proprietary drug or device testing be done?
	N	Any use of embryonic stem cells? *NOTE: If research involves embryonic stem cells, see UCB Stem Cell Policy and Committee.
	N	Any use of medical devices or equipment cleared/approved for marketing?
	N	Any use of any experimental or investigational devices or equipment (i.e., not cleared/approved for marketing?)
Y		Any use of commercially available drugs, reagents, or other chemicals administered to subjects (even if drugs themselves are not being studied)?
	N	Any use of investigational drugs, reagents, or chemicals (i.e., not cleared/approved for marketing)?

Protocol Title:	Measuring the benefits of sanitation, water quality, handwashing and nutrition interventions for improving health and development in rural Bangladesh
Protocol Status:	APPROVED
Date Submitted:	08/18/2014
Approval Period:	09/16/2014-11/03/2016
Important Note:	This Print View may not reflect all comments and contingencies for approval. Please check the comments section of the online protocol. Questions that appear to not have been answered may not have been required for this submission. Please see the system application for more details.

* * * Funding * * *

Funding Checklist

If the research is not funded, check the "Not Funded" box below. If the research is funded, add the funding source to the appropriate table below.

NOTE: Only the Principal Investigator (PI) of the grant or subcontract can add his or her own SPO Funding information in this section. The PI of the grant must also be listed in the Personnel Information section of the protocol in one of the following roles: Principal Investigator or Faculty Sponsor, Student or Postdoctoral Investigator, Co-Principal Investigator, Administrative Contact, or Other Contact. Training Grants can be added by anyone in one of the aforementioned roles. For step-by-step instructions, see Add SPO Funding Quick Guide

Not Funded

SPO - Funding

SPO ID	Sponsor	Sponsor Award ID	Prime Sponsor

SPO - Funding

SPO ID	20130310
Sponsor Award ID	
Sponsor	Bill & Melinda Gates Foundation
Prime Sponsor	
Funding Status	Pending
Principal Investigator	Colford Jr, John M
Co-Investigator (s)	
Admin Unit	SPH Administrative Services
Project Title	Measuring the Benefits of Sanitation, Water Quality, Handwashing and Nutrition Interventions for Improving Health and Development
Amount	$271,336
Start	9/17/2009
End	9/30/2015

**Protocol Title:**	Measuring the benefits of sanitation, water quality, handwashing and nutrition interventions for improving health and development in rural Bangladesh
**Protocol Status:**	APPROVED
**Date Submitted:**	08/18/2014
**Approval Period:**	**09/16/2014-11/03/2016**
**Important Note:**	**This Print View may not reflect all comments and contingencies for approval. Please check the comments section of the online protocol. Questions that appear to not have been answered may not have been required for this submission. Please see the system application for more details.**

**Subcontracts**	Yes

**SPO ID**	028064-002
**Sponsor Award ID**	OPPGD759
**Sponsor**	Bill & Melinda Gates Foundation
**Prime Sponsor**	
**Funding Status**	Active
**Principal Investigator**	Colford Jr, John M
**Co-Investigator (s)**	
**Admin Unit**	School of Public Health
**Project Title**	Measuring the Benefits of Sanitation, Water Quality and Handwashing Interventions for Improving Health and Development
**Amount**	$28,521,066
**Start**	9/17/2009
**End**	9/30/2015
**Subcontracts**	No

**SPO ID**	20131960
**Sponsor Award ID**	
**Sponsor**	NIH National Institutes of Health - Miscellaneous
**Prime Sponsor**	
**Funding Status**	Pending
**Principal Investigator**	Colford Jr, John M
**Co-Investigator (s)**	
**Admin Unit**	School of Public Health
**Project Title**	Effects of Sanitation of Pathogen Transmission and Child Health in Bangladesh
**Amount**	$2,692,294
**Start**	9/1/2013
**End**	8/31/2017

Protocol Title: Measuring the benefits of sanitation, water quality, handwashing and nutrition interventions for improving health and development in rural Bangladesh

Protocol Status: APPROVED

Date Submitted: 08/18/2014

Approval Period: 09/16/2014-11/03/2016

Important Note: **This Print View may not reflect all comments and contingencies for approval. Please check the comments section of the online protocol. Questions that appear to not have been answered may not have been required for this submission. Please see the system application for more details.**

**Subcontracts**	Yes

**SPO ID**	20141262
**Sponsor Award ID**	
**Sponsor**	Stanford University
**Prime Sponsor**	World Bank
**Funding Status**	Funded
**Principal Investigator**	Colford Jr, John M
**Co-Investigator (s)**	
**Admin Unit**	Dean's Office, Public Health
**Project Title**	Assessment of Exposure Pathways to Fecal Contamination, Assocation with Diarrhea and Sanitation Coverage in Bangladesh
**Amount**	$40,744
**Start**	6/1/2013
**End**	4/30/2014
**Subcontracts**	No

**SPO ID**	20140633
**Sponsor Award ID**	
**Sponsor**	NIH National Institute of Child Health & Human Development
**Prime Sponsor**	
**Funding Status**	Pending
**Principal Investigator**	Colford Jr, John M
**Co-Investigator (s)**	
**Admin Unit**	Dean's Office, Public Health
**Project Title**	Spillover Effects of Water, Sanitation, and Hygiene Interventions on Child Health
**Amount**	$368,089
**Start**	4/1/2014

**Protocol Title:**	Measuring the benefits of sanitation, water quality, handwashing and nutrition interventions for improving health and development in rural Bangladesh
**Protocol Status:**	APPROVED
**Date Submitted:**	08/18/2014
**Approval Period:**	**09/16/2014-11/03/2016**
**Important Note:**	**This Print View may not reflect all comments and contingencies for approval. Please check the comments section of the online protocol. Questions that appear to not have been answered may not have been required for this submission. Please see the system application for more details.**

**End**	3/31/2016
**Subcontracts**	Yes

**SPO ID**	20142940
**Sponsor Award ID**	
**Sponsor**	UC Davis
**Prime Sponsor**	Thrasher Research Fund
**Funding Status**	Funded
**Principal Investigator**	John M Colford Jr
**Co-Investigator (s)**	
**Admin Unit**	SPH Divisional Rsrch and Cntrs
**Project Title**	Evaluating the impact of water, sanitation, hygiene and nutrition interventions on infant micronutrient status and anemia risk in the WASH Benefits study
**Amount**	$37,597
**Start**	7/1/2015
**End**	3/31/2017
**Subcontracts**	No

Funding - Other

**Protocol Title:**	Measuring the benefits of sanitation, water quality, handwashing and nutrition interventions for improving health and development in rural Bangladesh
**Protocol Status:**	APPROVED
**Date Submitted:**	08/18/2014
**Approval Period:**	**09/16/2014-11/03/2016**
**Important Note:**	**This Print View may not reflect all comments and contingencies for approval. Please check the comments section of the online protocol. Questions that appear to not have been answered may not have been required for this submission. Please see the system application for more details.**

* * * Expedited Paragraphs * * *

Request for Expedited Review

An expedited review procedure consists of a review of research involving human subjects by the IRB Chair, or by one or more experienced reviewers designated by the Chairperson from among the members of the committees.

In order to be eligible for expedited review, ALL aspects of the research must include activities that (1) present no more than minimal risk to human subjects, and (2) involve only procedures included in one or more of the specific categories listed below.

**If requesting Expedited Review, select one or more of the applicable paragraph(s) below.
(DO NOT select any paragraph(s) if your protocol does not qualify for expedited review. Protocols that do not qualify for expedited review will be reviewed by the full (convened) Committee.)**

1. **Clinical studies of drugs and medical devices only when conditions (a) and (b) are met.**

   a) Research on drugs for which an investigational new drug application (21 CFR Part 312) is not required. (Note: Research on marketed drugs that significantly increases the risks or decreases the acceptability of the risks associated with the use of the product is not eligible for expedited review.)

   b) Research on medical devices for which

   i) an investigational device exemption application (21 CFR Part 812) is not required; or
   ii) the medical device is cleared/approved for marketing and the medical device is being used in accordance with its cleared/approved labeling.

X 2. **Collection of blood samples by finger stick, heel stick, ear stick, or venipuncture as follows:**

   a) From healthy, non-pregnant adults who weigh at least 110 pounds. For these subjects, the amounts drawn may not exceed 550 ml in an 8 week period and collection may not occur more frequently than 2 times per week; or

   b) From other adults and children, considering the age, weight, and health of the subjects, the collection procedure, the amount of blood to be collected, and the frequency with which it will be

collected. For these subjects, the amount drawn may not exceed the lesser of 50 ml or 3 ml per kg in an 8 week period and collection may not occur more frequently than 2 times per week.

X 3. **Prospective collection of biological specimen for research purposes by non-invasive means.**

Examples:

a) hair and nail clippings in a non-disfiguring manner;
b) deciduous teeth at time of exfoliation or if routine patient care indicates a need for extraction;
c) permanent teeth if routine patient care indicates a need for extraction;
d) excreta and external secretions (including sweat);
e) uncannulated saliva collected either in an unstimulated fashion or stimulated by chewing gumbase or wax or by applying a dilute citric solution to the tongue;
f) placenta removed at delivery;
g) amniotic fluid obtained at the time of rupture of the membrane prior to or during labor;
h) supra- and subgingival dental plaque and calculus, provided the collection procedure is not more invasive than routine prophylactic scaling of the teeth and the process is accomplished in accordance with accepted prophylactic techniques;
i) mucosal and skin cells collected by buccal scraping or swab, skin swab, or mouth washings;
j) sputum collected after saline mist nebulization.

X 4. **Collection of data through non-invasive procedures (not involving general anesthesia or sedation) routinely employed in clinical practice, excluding procedures involving x rays or microwaves. Where medical devices are employed, they must be cleared/approved for marketing. (Studies intended to evaluate the safety and effectiveness of the medical device are not generally eligible for expedited review, including studies of cleared medical devices for new indications.)**

Examples:

a) physical sensors that are applied either to the surface of the body or at a distance and do not involve input of significant amounts of energy into the subject or an invasion of the subject's privacy;
b) weighing or testing sensory acuity;
c) magnetic resonance imaging;
d) electrocardiography, electroencephalography, thermography, detection of naturally occurring radioactivity, electroretinography, ultrasound, diagnostic infrared imaging, doppler blood flow, and echocardiography;
e) moderate exercise, muscular strength testing, body composition assessment, and flexibility testing where appropriate given the age, weight, and health of the individual.

**Protocol Title:**	Measuring the benefits of sanitation, water quality, handwashing and nutrition interventions for improving health and development in rural Bangladesh
**Protocol Status:**	APPROVED
**Date Submitted:**	08/18/2014
**Approval Period:**	**09/16/2014-11/03/2016**
**Important Note:**	**This Print View may not reflect all comments and contingencies for approval. Please check the comments section of the online protocol. Questions that appear to not have been answered may not have been required for this submission. Please see the system application for more details.**

5. **Research involving materials (data, documents, records, or specimens) that have been collected or will be collected solely for non-research purposes (such as medical treatment or diagnosis). (NOTE: Some research in this paragraph may be exempt from the HHS regulations for the protection of human subjects. 45 CFR 46.101(b)(4). This listing refers only to research that is not exempt.)**

6. **Collection of data from voice, video, digital, or image recordings made for research purposes.**

7. **Research on individual or group characteristics or behavior (including, but not limited to, research on perception, cognition, motivation, identity, language, communication, cultural beliefs or practices, and social behavior) or research employing survey, interview, oral history, focus group, program evaluation, human factors evaluation, or quality assurance methodologies. (NOTE: Some research in this category may be exempt from the HHS regulations for the protection of human subjects. 45 CFR 46.101(b)(2) and (b)(3). This listing refers only to research that is not exempt.)**

8. **Continuing review of research previously approved by the convened IRB as follows:**

   a) Where (i) the research is permanently closed to the enrollment of new subjects; (ii) all subjects have completed all research-related interventions; and (iii) the research remains active only for long-term follow-up of subjects; or

   b) Where no subjects have been enrolled and no additional risks have been identified; or

   c) Where the remaining research activities are limited to data analysis.

9. **Continuing review of research, not conducted under an investigational new drug application or investigational device exemption where categories two (2) through eight (8) do not apply but the IRB has determined and documented at a convened meeting that the research involves no greater than minimal risk and no additional risks have been identified.**

Protocol Title: Measuring the benefits of sanitation, water quality, handwashing and nutrition interventions for improving health and development in rural Bangladesh

Protocol Status: APPROVED

Date Submitted: 08/18/2014

Approval Period: 09/16/2014-11/03/2016

Important Note: **This Print View may not reflect all comments and contingencies for approval. Please check the comments section of the online protocol. Questions that appear to not have been answered may not have been required for this submission. Please see the system application for more details.**

* * * Purpose, Background, Collaborative Research * * *

Old CPHS # (for Protocols approved before eProtocol)

Study Title

Measuring the benefits of sanitation, water quality, handwashing and nutrition interventions for improving health and development in rural Bangladesh

Complete each section. When a question is not applicable, enter "N/A". Do not leave any sections blank.

1. Purpose

Provide a brief explanation of the proposed research, including specific study hypothesis, objectives, and rationale.

The goal of the WASH Benefits study is to generate rigorous evidence about the impacts of sanitation, water quality, and handwashing (WASH) and nutrition interventions on child health and development in the first years of life. The primary hypotheses of the study are:
H1: Water, sanitation, handwashing, nutrition and their combination improve child health and development.
H2: When delivered in combination, water, sanitation and handwashing interventions reduce child diarrhea more than when delivered individually.
H3: Combined Nutrient supplementation and WASH interventions improve child growth and development more than nutrient supplementation alone.

The study objectives are to:

1) Rigorously measure health benefits arising from low-cost WASH approaches including local promoters and subsidies for simple technologies (e.g. latrine improvements or potties for children, chlorine dispensers, and handwashing stations) and evaluate the degree to which, in resource-constrained settings, there is added health benefit to delivering multiple interventions concurrently (sanitation services, drinking water, and handwashing promotion)

Most of the burden of diarrheal disease is thought to be preventable with improvements in sanitation, water quality, and hygiene. However, in rural areas of low-income countries it is often prohibitively expensive to provide residents with networked sanitation and water treatment that provide microbiologically and chemically safe water and consistently separate feces from the environment. We have almost no evidence that allows direct comparison of the health benefits or cost-effectiveness of improvements in sanitation, water quality, and hygiene, nor on how the benefits of these interventions aggregate when provided in combination. Such evidence is critical for guiding the allocation of public and donor funds to achieve the maximum health impact given limited resources. In the absence of credible evidence, little change from the status quo can be expected, even if the impact of current practices is unknown. A rigorous evaluation of these interventions that documents changes in outcomes associated with long-run economic success, such as early childhood growth, could have at least two important influences on policy. First, such evidence could help to maximize the value of existing resources by shifting expenditures to the most cost-

---

effective interventions. In addition, such evidence could help generate more resources for these sectors by resolving uncertainty regarding the efficacy of water, sanitation, and hygiene interventions and identifying simple technologies and approaches to behavior change that cost-effectively improve health and could be replicated at scale.
2) Measure the impact of lipid-based nutrient supplementation (LNS) alone and in combination with sanitation, water and hygiene interventions on child growth and development.
For children whose food intake is insufficient, LNS helps reduce gross energy shortfalls and provide essential micronutrients. The energy and micronutrients that LNS provides are likely to improve nutritional indicators, length-for-age and child development, particularly among children that are at highest risk for severe stunting. It is possible that improved nutrition alone can reduce the negative effects of infection on growth and development due to the improved ability of better-nourished children to fight off enteric infections and exhibit catch-up growth during the convalescent period.
A combined LNS+WASH intervention could have greater impacts on growth and development than LNS alone. The reasons for this are two-fold. First, the likely reciprocal relationship between enteric infection and malnutrition in young children suggests that the provision of joint interventions that interrupt both components of the “vicious cycle” may have effects that exceed interventions that interrupt just one component. Indeed, if there is a reciprocal relationship between enteric infection and malnutrition, the feedback loop toward decline could, in principal, be reversed and leveraged to enhance growth and development. There is a second plausible scenario that could result in larger combined effects: if the interventions are deployed together, the available energy for growth and development could be enhanced by improved utilization of the additional nutrition provided by LNS that would have not been available to the child without complementary infection control. Such improved utilization could be achieved by reductions in acute diarrheal disease and the chronic symptoms that characterize environmental enteropathy.
3) Measure the impact of nutritional supplements and household environmental interventions on environmental enteropathy biomarkers, and more clearly elucidate this potential pathway between environmental interventions and child growth and development.
If improvements in sanitation, water quality, and hand hygiene could reduce the severity of intestinal malabsorption from environmental enteropathy (EE) either by preventing its acquisition or by reversing the pathology, this would represent an important contribution to global public health. EE is an inflammatory disorder of the small intestine that results in reduced nutrient absorption and increased gut permeability (and thus increased immune system stimulation). The scientific literature to date suggests that EE is most likely caused by poor sanitation, water quality and hygiene in low income countries, but there are no studies that demonstrate a specific association between EE and environmental conditions separated from other exposures in a low income environment. The WASH Benefits study is uniquely positioned to gather randomized evidence about the impact of household environmental interventions on EE in young children. Furthermore, the study will also be positioned to gather rigorous evidence about the independent and combined impact of supplemental nutrition and WASH interventions on EE biomarkers.
4) Measure the impact of WASH and nutrition interventions on intestinal parasitic infection prevalence and intensity.
Observational studies suggest that environmental interventions can reduce parasite infection if sanitation and hygiene conditions improve in a large share of the population. Pre-school aged children spend much of their time in the immediate home environment. It is with this in mind that the WASH Benefits study focuses on household environmental interventions. There is a large body of evidence that documents household level clustering and within-household transmission of Cryptosporidium, Giardia, and E. histolytica, as well as soil-transmitted helminthes (Ascaris, Trichuris, and hookworm). The observed patterns suggest that intestinal parasites may be a useful marker of enteric pathogen transmission more broadly. The simultaneous measurement of parasitic infections, caregiver-reported diarrhea, and

environmental enteropathy biomarkers will allow us to explore this secondary hypothesis.

5) Measure the impact of interactions between the water, sanitation, hygiene and nutritional interventions and the mother and child's intestinal microbiome, immune function, internal biochemical environment and genetic disposition.

We expect that the interventions will have different effects in different children and that some of these differences will be mediated by differences in the mother and child's intestinal microbiome, immune function, internal biochemical environment and genetic disposition. We will collect maternal and child samples to permit future assessment of mother and child's intestinal microbiome, immune function, internal biochemical environment and genetic disposition, and so explore the importance of interactions between these characteristics and study outcomes. Measurements of immune function, internal biochemical environment including nutrition and genetic characteristics are especially dynamic areas of scientific research where important new insights are emerging each month. The capacity to explore these factors will provide the opportunity to understand the mechanism and generalizability of any impacts on child health and development that are observed within the study.
For example a child who acquires a specific population of microbiological organisms from her mother's intestinal microbiome may be more resistant to infection with intestinal pathogens and so less likely to benefit from sanitation interventions. As a second example, the haplotype HLA AW-31 is strongly associated with the development of tropical sprue, which is a disease of the small intestine that affects intestinal absorption (Menendez-Corrada R, Nettleship E, Santiago-Delfin E. 1986. HLA and Tropical Sprue. Lancet 328:1183-1185.). We would expect that interventions to impact this small intestinal pathology would be different in the presense of persons who genetically have HLA AW-31 compared to those who do not. This example illustrates the potential importance of genotype in the development of intestinal pathology, which could consequently modify the efficacy of the interventions. By exploring genetic characteristics, such as HLA AW-31 and those that will be described in the coming 20 years, we set ourselves up to optimally understand the mechanism of interaction between genetic characteristics and intervention efficacy.

As part of the assessment of lead, a particularly potent neurotoxin that can affect child intellectual development, we plan to leverage the study to assess the prevalence of lead exposure, identify the environmental source and pathway, and strive to understand the primary incentives that encourage environmental contamination with lead.

2. Background

Give relevant background (e.g., summarize previous/current related studies) on condition, procedure, product, etc. under investigation, including citations if applicable (attach bibliography in Attachments section).

An estimated 2.2 million children under the age of 5 years die from diarrheal disease each year (WHO 2008). Children who survive multiple episodes of diarrhea and enteric infections commonly develop environmental enteropathy, an inflammatory disorder of the intestines that compromises nutrient absorption (Haghighi 1997). Repeated episodes of diarrhea and chronic environmental enteropathy in early childhood reduce growth and cognitive function, and impair school performance (Alderman 2006,

---

Protocol Title: Measuring the benefits of sanitation, water quality, handwashing and nutrition interventions for improving health and development in rural Bangladesh

Protocol Status: APPROVED

Date Submitted: 08/18/2014

Approval Period: 09/16/2014-11/03/2016

Important Note: **This Print View may not reflect all comments and contingencies for approval. Please check the comments section of the online protocol. Questions that appear to not have been answered may not have been required for this submission. Please see the system application for more details.**

Checkley 2008, Lorntz 2006, Niehaus 2002, Petri 2008). This in turn can reduce income later in life (Boissiere 1985). Thus, repeated episodes of childhood diarrhea and enteric infection may exact a long-run toll, perpetuating a cycle of poverty and ill health.

Water, Sanitation, and Handwashing Interventions
Most of the burden of diarrheal disease is thought to be preventable with improvements in sanitation, water quality, and hygiene (Ezzati 2003). However, in rural areas of low-income countries it is often prohibitively expensive to provide residents with networked sanitation and water treatment that provide microbiologically and chemically safe water and consistently separate feces from the environment.

This has led to a movement towards alternative non-networked solutions including improved sanitation efforts, efforts to increase water treatment by households, and programs to increase handwashing with soap. Observational studies suggest that reducing open defecation is potentially important in reducing the transmission of both diarrhea and trachoma (Esrey 1996, Esrey 1991). There is evidence that water treatment, in particular with dilute chlorine solution, can reduce self-reported diarrhea (Arnold 2007, Clasen 2007). There is also strong evidence that handwashing with soap can dramatically reduce self-reported diarrhea as well as other diseases (Ejemot 2008, Rabie 2006). Some researchers however have called for evidence on more objective measures such as physical growth and cognition, rather than reports by family members (Schmidt 2009). We have almost no evidence that allows direct comparison of the health benefits or cost-effectiveness of improvements in sanitation, water quality, and hygiene, nor on how the benefits of these interventions aggregate when provided in combination.

Improved Sanitation
Improved sanitation effectively separates human excreta from human contact and the environment. The most common sanitation technologies in developing countries, particularly in rural areas, are various forms of private latrines. Observational studies suggest that households that receive improved sanitation experience 24% less diarrhea than households without sanitary facilities (Daniels 1990, Barreto 2007). However, there has never been a randomized controlled trial (RCT) to confirm these observational findings.

Full subsidies for appropriately designed latrines have resulted in high levels of coverage and regular use. For example, in the Gambia improved pit latrines were provided free of charge to 666 households in 32 villages. After 25 to 47 months each household was revisited; 77% of the provided latrines were still in use and 97% of latrines owners said they would make a new latrine when their current one was full (Simms 2005). In an evaluation of one of the Carter Center's subsidized latrine provision programs that included a random sample of 200 households across 50 villages in Niger, 86% of latrines were in regular use and 70% were clean after one year during unannounced visits (Diallo 2007). In southern India, a recent observational study found that despite high levels of latrine coverage (57%) following a community mobilization campaign, 40% of households with toilets continued to defecate in the open and there was no improvement in child diarrhea or growth – suggesting that health impacts are not guaranteed by high coverage and are likely context dependent (Arnold 2010).

The vast majority of gastrointestinal illness is caused by fecal-oral pathogen transmission through complex, environmentally mediated pathways including drinking water, ambient waters, hands, food, soil and vectors. Sanitation coverage is a primary barrier measure that aims to prevent fecal contamination from entering the environment and could plausibly break all of these pathways. However, it is uncertain whether increasing sanitation coverage effectively reduces environmental contamination and there are

conflicting findings on the impact of sanitation improvements on child health.

Handwashing Promotion
There is significant evidence from randomized controlled trials that households receiving intense encouragement to regularly wash their hands with soap have less self-reported diarrhea and respiratory disease than households who continue their normal hand hygiene practices (Ejemot 2008, Rabie 2006). However, the existing evidence comes from trials which used very intensive interventions to encourage handwashing interventions that would not be practical to implement at scale. A key issue is the difficulty in knowing how to encourage greater take up of handwashing at reasonable cost.

One key barrier to handwashing is the difficulty and high use of water required in filling a basin, washing hands in the basin, and emptying the basin. In an observational evaluation in rural Bangladesh, households that had soap or water at their most convenient place to wash hands were twice as likely to wash their hands with soap after fecal contact than households that lacked these essential supplies (Luby 2009) [29]. Of course causality is not clear in the absence of a randomized controlled trial. A variety of simple low-cost handwashing stations have been developed which provide a place to wash hands and a source of flowing water. These can be as simple as plastic containers which one can tilt to create a stream of water by pulling rope with one's feet, or plastic containers plugged with sticks, which release a trickle of water when the stick is removed. A well-placed handwashing station provides a visual cue to spur handwashing and greatly increases the convenience of handwashing. There is considerable evidence across the social sciences that convenience is a key factor in promoting behavior change. In fact, one important lesson from the literature on behavioral change is that making something easy can be more effective at inducing change than education or promotional messaging (Kaplan 1986, Sallis 2008, Kremer 2009). Additionally, if they are placed outside of the latrines they are at least partially in public view, and there is evidence at least in a western context that people are much more likely to wash hands after using a toilet if they believe they may be observed (Pederson 1986, Ram et al 2010).

Water Treatment
There is little evidence that providing water supplies that meet the engineering definition of "improved" lead to health or social benefits for the population. In contrast, evidence from settings where diarrhea is a leading cause of death shows that improving the microbiological quality of drinking water markedly reduces reported diarrhea (Clasen 2006, Fewtrell 2005, Arnold 2007). Randomized controlled trials conducted using various household-based point-of-use water treatment technologies have demonstrated that households that consume regularly treated water report substantially less diarrhea than households using untreated water. However, self-reported diarrhea, which is the outcome measure for the majority of these studies, may be subject to measurement error potentially correlated with treatment. For this reason, long-term objective outcomes such as those planned for the proposed study (i.e. anthropometric measurements, cognition, and environmental enteropathy) may prove more convincing from a policy perspective and may be more likely to motivate action from policy makers (WHO & UNICEF Progress on drinking water & sanitation 2008).

Chlorination is the household water treatment solution of choice in many contexts given its safety and cost-effectiveness. It has been used in piped water systems around the world for almost a century. In many places where such infrastructure is absent or imperfectly maintained, dilute chlorine solution is marketed as a consumer good used in the home. Although chlorinating household drinking water reduces reported diarrhea by 20-40%, take-up has been low under the current social marketing model. Members of the

study team for this proposal have developed and piloted a chlorine dispenser in rural Kenya, which is a simple device that is installed at communal water sources to enable water treatment at the point of collection. When the dispenser is provided along with a local promoter to encourage its use, we find that take-up is on the order of 60-70% in communities with access to a dispenser, as compared to 5-10% in communities with access only to the traditional model of chlorine distribution. Moreover, in contrast to other strategies that we tested, chlorine use appears to be stable or even rising over time, likely because the dispenser technology makes water treatment cheap and easy for users, and harnesses positive peer effects: the public nature of the dispenser allows community members to implicitly and explicitly remind each other to treat their water.
In Bangladesh the study will use Aquatabs for chlorination. Aquatabs are effervescent water purification tablets that utilize sodium dichloroisocyanurate (NaDCC) as the chlorine donor. NaDCC was judged to be a safe and appropriate treatment for water by the World Health Organization (Clasen and Edmondson, 2006). In a previous field study in Geneva slum in Dhaka, use of Aquatabs consistently yielded an appropriate chlorine dose and dramatically improved drinking water quality (Clasen 2007). In pilot testing in rural Bangladesh Aquatabs provided with a safe water storage container were acceptable to the community with 78% of households having chlorine residual in store drinking water on unannounced follow-up visits.

Pilot Work (2010-12-2601)
The WASH Benefits pilot work has allowed the study teams to refine the sanitation, handwashing and water quality interventions and identify hardware and behavior change packages that result in high levels of uptake.

Based on the results of the pilot work, the following water, sanitation, and hygiene interventions will be implemented:

Intervention class

Sanitation: Sanitation promotion, child potties, sani-scoop hoes to remove feces from household environments, latrine upgrades to a dual pit latrine

Handwashing: Promotion of handwashing with soap or waterless hand sanitizers at critical times, handwashing stations, soapy water at handwashing locations

Water quality: Chlorine tablets (Aquatabs) + safe storage vessels, water treatment promotion
----

Nutrition Intervention
There is abundant evidence that the prenatal period and the first two years of life are a critical window for intervention in growth and development: infection and poor nutrition during this window can negatively impact an individual's long-term cognitive development and lifetime physiologic trajectory (Checkley 2003, Berkman 2002, Black 2008, Guerrant 1999, Niehaus 2002, Tarleton 2006, Bhutta 2008, Crimmins 2006, Grantham-McGregor 2007, Victora 2010). Nutritional interventions during the first years of life improve schooling and income in adolescents and adults up to 35 years later (Victora 2008, Hoddinott 2008). Yet, a systematic review of the impacts of complementary feeding and supplementation interventions reports that

even the most successful of these interventions increase length-for-age Z-scores by 0.69 SDs, which is approximately 1/3 of the mean growth deficit for African and Southeast Asian populations (the mean intervention effect is 0.28 SDs) (Dewey 2008).

One hypothesis for why nutritional supplementation appears to be necessary but not sufficient to eliminate growth shortfalls is that chronic infection and colonization of the small intestine by fecal bacteria impedes nutrient absorption and creates low-level immune system stimulation, a condition called environmental enteropathy (Lunn 2000). Environmental enteropathy is characterized by damage to mucosa in the wall of small intestine that decreases its surface area for nutrient absorption and increases its permeability to antigenic molecules that stimulate immune system defenses (Lunn 2000, Campbell 2003). Biomarkers for intestinal permeability and immune system stimulation have been more strongly associated than acute diarrhea with growth shortfalls (Campbell 2003). The mucosal damage that characterizes environmental enteropathy is caused by the body's inflammatory response to the ingestion of fecal bacteria, and when people move to lower-bacteria environments the condition resolves (Haghighi 1997). Recently, nutritionists have hypothesized that reducing a child's fecal bacteria exposure during the first years of life through improved sanitation, handwashing or water treatment may improve gut function and subsequent growth (Humphrey 2009).

For children whose food intake is insufficient, lipid-based nutritional supplementation (LNS) helps reduce gross energy shortfalls and provide essential micronutrients. The energy and micronutrients that LNS provides are likely to improve nutritional indicators, length-for-age and child development (Adu-Afarwuah 2008, Adu-Afarwuah 2007, Dewey 2008, Walker 2007, Rosales 2009, Bryan 2004), particularly among children that are at highest risk for severe stunting (Phuka 2008, Phuka 2009). It is possible that improved nutrition alone can reduce the negative effects of infection on growth and development due to the improved ability of better-nourished children to fight off enteric infections and exhibit catch-up growth during the convalescence period (Guerrant 1992, Guerrant 2008).

The specific LNS we propose to use is a next generation supplement to Nutributter; members of our team (Drs. Dewey and Steward at UC Davis) have been involved in the development of the supplement and are currently deploying it in ongoing randomized, controlled trials in Bangladesh, Burkina Faso, Ghana and Malawi as part of the iLiNS project and related studies (iLiNS.org). Nutributter and related LNS interventions have demonstrated efficacy for improving child growth and development when provided daily after age six months (Adu-Afarwuah 2008, Adu-Afarwuah 2007). We propose a combined energy / micronutrient supplement because micronutrient supplementation alone is unlikely to have a large impact on linear growth (Ramakrishnan 1989). LNS is administered daily using 10 gram sachets that can be mixed into existing meals (e.g., porridge); a child eats two sachets per day. LNS is intended to supplement – and not replace – breastfeeding and locally available complementary foods, by providing 108 kcal/day and including a broad suite of essential fatty acids and micronutrients at dosages appropriate for children in this age group. It has an 18-month shelf life, does not spoil at high temperatures and costs as little as $0.10 per day. Its compliance has been over 88% in controlled trials (Adu- Afarwuah 2008), in part due to the ease of incorporating it into existing feeding routines. Breastfeeding is highly prevalent in both populations, and so we have focused on supplements that would not replace this essential source of nutrition (Black 2008).

Our collaborators at UC Davis have a series of ongoing randomized trials evaluating the impact of LNS supplementation provided to pregnant and lactating women and/or their infants in Ghana, Malawi, and Burkina Faso through the International Lipid Based Nutrient Supplementation Project (www.ilins.org). The

objectives of the project include the development of low-cost, acceptable LNS formulations using locally available ingredients and evaluation of the efficacy of reduced cost formulations of LNS for infants, young children and pregnant women. Acceptability trials have been conducted in all the three of the countries with positive results. Importantly, the iLiNS project has already demonstrated that LNS is acceptable among young children in similar cultures to the rural Bangladesh population.

Biomarkers for Environmental Enteropathy
Environmental enteropathy, an inflammatory disorder of the intestines that compromises nutrient absorption, is associated with child malnutrition and poor development (Haghighi 1997, Humphrey 2009, McKay 2010). Environmental enteropathy is one of the main hypothesized pathways for the impact of our interventions on growth and development. Measurement of environmental enteropathy symptoms will provide important information about the mechanism for intervention impacts (or lack of impact) in this study. Altered intestinal permeability is an indicator of environmental enteropathy, measured using a dual-sugar permeability test in which the lactulose:mannitol urinary excretion ratio is measured (Lunn 2000, Campbell 2003). The child is given a combination of the two sugars, lactulose and mannitol. Mannitol diffuses through a transcellular pathway and is used to assess the absorptive capacity and mucosal surface area of the enterocytes. Lactulose is typically minimally absorbed via the paracellular tight junctions and thus, it is used to assess epithelial integrity. A normal intestinal epithelium absorbs nearly all mannitol, but almost no lactulose. A damaged epithelium absorbs mannitol less efficiently and more lactulose. By measuring the lactulose: mannitol ratio in the urine passed over the subsequent 3-5 hours, the intestinal absorptive efficiency can be calculated and the severity of environmental enteropathy inferred.

Earlier studies demonstrated that environmental enteropathy as assessed by intestinal absorption is widespread in low income tropical countries where fecal contamination of water, food, and the environment are common in contrast to rarely being seen among normal residents of high income temperate countries (Haghighi 2003). Environmental enteropathy is acquired early in childhood. Stillborn children in tropical countries have normal intestinal small intestinal cellular structure (Haghighi 1979). During the first three months of life mannitol/lactulose absorption is normal in children in The Gambia compared to children in the UK, however after three months intestinal absorption among Gambian children progressively decreases during the first year of life (Lunn 1991). Recent studies in Bangladesh confirm intestinal malabsorption consistent with environmental enteropathy is present in children 3 – 24 months of age in the rural Dhamrai subdistrict; the degree of impairment in absorption increased in children between 3 and 12 months of age (Goto 2009a, Goto 2009b). The intestinal absorption and pathology of migrants who move from highly contaminated low income tropical countries to developed temperate countries normalizes within 3 to 5 years (Gerson 1971).

If improvements in sanitation, water quality, hand hygiene and nutrition could reduce the severity of intestinal malabsorption from environmental enteropathy either by preventing its acquisition or by reversing the pathology, this would represent an important contribution to global public health, and would be a useful outcome assessment for the larger planned intervention study. Nutritionists have recently argued that environmental enteropathy is most likely caused by poor sanitation and hygiene in low income countries (Humphrey 2009, McKay 2010). Yet, there are no studies that demonstrate a specific association between environmental enteropathy and poor sanitation separated from other exposures in a low income country environment, although one study from Rhodesia 30 years ago noted an association between intestinal absorption and socioeconomic status (Thomas 1976).

Pilot Environmental Enteropathy Work in Bangladesh (2010-11-2536)

In our Bangladesh environmental enteropathy pilot study, we selected 119 children from an existing cohort (SHEWA-B intervention assessment study) who lived in different levels of household environmental cleanliness based on sanitation, water quality and handwashing indicators. The children were between age 8 and 48 mo in May 2010 and lived in 83 different rural villages across Bangladesh.

The 66 children from households with improved household hygiene lived in homes with good sanitation (flush/septic/piped sewerage or a pit latrine with slab and water seal), good water quality (median E. coli < 10 CFU/100 ml in up to 8 samples collected over 24 mo), and favorable handwashing conditions (a dedicated location to wash hands stocked with soap and water). In contrast, the 53 children who lived in homes with poor household hygiene lacked adequate sanitation (open defecation, open pit latrines, slabs with broken water seals, toilets that flush to “somewhere else” or hanging toilets), had poor water quality (median E. coli ≥ 10 CFU/100 ml), and had unfavorable handwashing conditions (no dedicated location to wash hands, or a dedicated location that lacked either water or soap). The definitions of improved hygienic conditions were chosen to reflect indicators that we hope to improve through intervention in the WASH Benefits study.

Children in the two environments differed greatly in their growth: after statistical adjustment for potentially confounding differences, children in households with improved hygienic conditions had 0.54 SDs (95%CI 0.06, 1.01) higher HAZ than children in households with poor hygienic conditions (unadjusted difference = 0.91 SDs). Importantly, the children also differed in biomarkers for environmental enteropathy. After statistical adjustment for measures of socioeconomic status, children living in improved hygienic households had lactulose : mannitol (L:M) ratios that were –0.32 SDs lower than children living in poor hygienic conditions (95% CI –0.72, 0.08). Children in improved hygienic households also had lower Immunoglobulin G endotoxin core antibody (IgG EndoCAb) titers (–0.23 SDs, 95% CI: –0.63, 0.17) than children living in poor hygienic conditions. After adjusting for age and sex, the L:M ratio was also strongly associated with HAZ in the population: a 1-unit increase in the log L:M was associated with a –0.36 SDs reduction in HAZ (95% CI –0.64, –0.07).

These pilot results support our original rationale to conduct the main WASH Benefits study. However, because household environmental conditions in the pilot were not randomized, it remains possible that differences observed between the children in growth and EE biomarkers result from unmeasured or unquantifiable differences between groups that we cannot control for without an experiment. A randomized trial that delivers high impact household environmental interventions (i.e., interventions with good uptake and high efficacy at reducing pathogen transmission to young children) in large populations as we have in our Kenya and Bangladesh cohorts would provide more conclusive evidence.

Effect of the interventions on telomere length and allostatic load

Multiple in utero and early life exposures to biological and psychosocial stress may increase allostatic load (the cumulative biological damage from chronic stress) and increase susceptibility to disease later in life(Entringer et al., 2010; Shonkoff et al., 2009; Tomiyama et al., 2012). The attrition of telomeres, the repetitive DNA sequences protecting the tips of chromosomes, may serve as a biomarker of cumulative lifetime stress or play a causal role in the etiology of various diseases, or both(Entringer et al., 2011).

Protocol Title: Measuring the benefits of sanitation, water quality, handwashing and nutrition interventions for improving health and development in rural Bangladesh

Protocol Status: APPROVED

Date Submitted: 08/18/2014

Approval Period: 09/16/2014-11/03/2016

Important Note: This Print View may not reflect all comments and contingencies for approval. Please check the comments section of the online protocol. Questions that appear to not have been answered may not have been required for this submission. Please see the system application for more details.

Telomere attrition may contribute to chromosomal instability, premature apoptosis, and organ damage(Armanios, 2013; Calado and Young, 2009). During the sensitive period of early postnatal life, cellular replication occurs at a rapid rate as the immune system, brain, and other systems develop(Zeichner et al., 1999). Since telomeres are a key determinant of tissue development and shorten at a dramatically faster rate in infancy compared to in adulthood, it is efficient to focus on early childhood factors that may accelerate telomere attrition(Frenck et al., 1998; Zeichner et al., 1999). Although telomere attrition within the context of various diseases in adult populations has been widely studied(Calado and Young, 2009; Lin et al., 2012), little is known about the pregnancy and early life risk factors associated with telomere attrition in infants from low-income countries. Complex pathways connect early life insults – micronutrient deficiencies, environmental enteropathy, and family violence – to adverse child health outcomes, and accumulating evidence implicates telomere attrition, allostatic load, inflammation, and growth factors as potentially important underlying mechanisms linking these environmental stressors and disease susceptibility. The trial design will enable us to a) measure the effect of the interventions on telomere attrition, b) examine the association between environmental enteropathy, telomere attrition, linear growth faltering, and poor cognitive development, and c) evaluate the impact of maternal psychological stress on child allostatic load, telomere length, growth trajectories, and cognitive development.

Micronutrient deficiencies may accelerate telomere attrition in children. Since the vast majority of cells are engaged in the DNA synthesis phase during early childhood development, the additive or synergistic effects of several micronutrient deficiencies could produce destructive effects on genome stability leading to negative health sequelae later in life(Fenech, 2005). Micronutrients maintain the genome by serving as cofactors for enzymes, participating in DNA synthesis and repair, and inhibiting oxidative stress-induced DNA damage(Bull and Fenech, 2008). In Bangladeshi children ages 24-48 months, 97% of children had inadequate folate intake(Arsenault et al., 2013). The thymidine-rich telomere repeat sequence, (TTAGGG)n, may be highly susceptible to folate-deficient conditions that favor the incorporation of elevated levels of uracil into the DNA rather than thymidine, which then leads to chromosome breaks(Blount et al., 1997). The impact of specific micronutrients on telomere length requires further study, and telomere attrition, in turn, may emerge as a sensitive marker of nutritional deficiency. This trial would be the first to examine the potential association of micronutrient deficiencies and telomere attrition and to measure the impact of nutrition interventions on telomere lengths in children.

The chronic infections and inflammation endemic in low-income countries with poor WASH may contribute to telomere attrition. When human adult subjects were experimentally exposed to a common cold virus, those with longer telomeres displayed more resistance to acute upper respiratory infection and clinical illness compared to those with shorter telomeres(Cohen et al., 2013). Celiac disease and environmental enteropathy share similar histologic features of intestinal inflammation, and the telomeres from small intestinal biopsies in individuals with celiac disease were shorter than the telomeres of healthy controls(Cottliar et al., 2003). An important question to explore within the context of the study is whether environmental enteropathy affects telomere attrition or whether telomere attrition exacerbates or increases susceptibility to environmental enteropathy. Furthermore, we will elucidate the potential associations between telomere lengths, linear growth, and cognitive development.

The association between psychological stress, telomere attrition, and growth faltering could be mediated through glucocorticoid and immune activation and oxidative stress. The hypothalamic-pituitary-adrenal (HPA) axis serves a vital role in the neuroendocrine systemic response to stress, and its contribution to telomere attrition has not yet been elucidated. Studies have demonstrated that chronic stress leads to glucocorticoid receptor resistance, a decreased sensitivity of immune cells to cortisol(Cohen et al., 2012;

Protocol Title: Measuring the benefits of sanitation, water quality, handwashing and nutrition interventions for improving health and development in rural Bangladesh

Protocol Status: APPROVED

Date Submitted: 08/18/2014

Approval Period: **09/16/2014-11/03/2016**

Important Note: **This Print View may not reflect all comments and contingencies for approval. Please check the comments section of the online protocol. Questions that appear to not have been answered may not have been required for this submission. Please see the system application for more details.**

Miller et al., 2002). Due to a lack of glucocorticoid regulation, a prolonged pro-inflammatory cytokine response ensues causing damage to multiple systems throughout the body. This dysregulation of the pro-inflammatory response is particularly detrimental when it occurs during the first two years of a child's life, the critical window of growth, because it negatively affects the growth hormone/insulin-like growth factor 1 (GH/IGF-1) endocrine axis. Growth hormone stimulates the secretion of IGF-1, an important regulator of cell proliferation, immunity, and inflammation(Deelen et al., 2014). During acute stress or infections, the body utilizes the pro-inflammatory cytokines to restrict growth and energy storage and instead, redirects energy to ensure survival(O'Connor et al., 2008). During the first two years of life, a period of rapid growth and development for a child, chronic stress induces a protracted pro-inflammatory response that diverts energy towards the immune system by dampening the anabolic activities of IGF-1 and instead promoting protein catabolism, thereby contributing to childhood stunting(Livingstone, 2013). Several child cohort studies have suggested associations between increased cortisol secretion, decreased IGF-1 levels, and greater risk of growth faltering(Cianfarani et al., 2002; Cianfarani et al., 1998; Idohou-Dossou et al., 2003; Kilic et al., 2004). The potential association between IGF-1 and telomeres has not yet been examined in children. Two potential applications of understanding this molecular pathway are 1) to develop biomarkers to evaluate the efficacy of interventions and 2) to generate possible targets for intervention to alter child growth trajectories.

Overall, this study will extend our understanding of how biological and social determinants, specifically micronutrient deficiencies, environmental enteropathy, and psychological stress, shape child health outcomes on a cellular level. This trial will be the first to evaluate the impact of water, sanitation, hygiene, and nutrition interventions on telomere length in infants living in Bangladesh. The insights from this research could build upon existing foundations to implement and assess holistic strategies to improve nutrition, decrease fecal contamination, reduce family stress, and ultimately cultivate a healthy environment to promote telomere elongation and advance child development.

Assessment of interactions

Since intervention assignment will be randomized the study groups will have similar population characteristics that will permit inferring that observed differences in outcome are attributable to the intervention. However, we expect that the interventions will have different effects in different children and that some of these differences will be mediated by differences in the mother and child's intestinal microbiome, immune function, internal biochemical environment and genetic disposition. For example a child who acquires a specific pattern of microbiological organisms from her mother's intestinal microbiome may be more resistant to infection with intestinal pathogens and so less likely to benefit from sanitation interventions. Immune function, internal biochemical environment including nutrition and genetic characteristics are especially dynamic areas of scientific research where important new insights are emerging each month. The capacity to explore these issues will provide the opportunity to understand the mechanism and generalizability of any impacts on child health and development that are observed within the study.

One exposure that has the potential to interact with child development is lead. Humans exposed to lead experience irreversible impairment of intellectual function (Bellinger et al., 1992). Two studies of residents living in rural Bangladesh remote from roads and industry report unexpectedly high blood lead levels including 14% of children in a rural area of Dinajpur District having blood lead levels >10 μg/dL (Mitra et

---

Protocol Title: Measuring the benefits of sanitation, water quality, handwashing and nutrition interventions for improving health and development in rural Bangladesh

Protocol Status: APPROVED

Date Submitted: 08/18/2014

Approval Period: 09/16/2014-11/03/2016

Important Note: **This Print View may not reflect all comments and contingencies for approval. Please check the comments section of the online protocol. Questions that appear to not have been answered may not have been required for this submission. Please see the system application for more details.**

al., 2009) (twice the current 5 µg/dL level used to identify US residents at high risk (Advisory Committee on Childhood Lead Poisoning Prevention, 2012)) and postpartum women in rural Matlab, where the median equivalent blood lead concentration of 6.0 µg/dL exceeded the high risk threshold (Bergkvist et al., 2010). A few studies have explored potential sources of lead in rural Bangladesh. Lead concentrations in soil used for agriculture in Mymensingh District, Bangladesh was twice as high as soil collected from adjacent plots used for non-agricultural domestic purposes (40.6 ppm versus 20.7 ppm) (Muhibbullah et al., 2005). Rice, the primary dietary staple in Bangladesh, collected from households in the Matlab study contained a median of 25 µg/kg of lead (Bergkvist et al., 2010).

Environmental Microbial Assessment

Fecal-oral pathogen transmission is a complex process. The complexity arises from a multitude of transmission pathways, a broad diversity of pathogens, the importance of environmental conditions, and interactions between the environment and human behavior. Water, sanitation and hygiene interventions present primary and secondary barriers that separate feces from the environment and should block enteric pathogen transmission. Measuring fecal contamination along environmentally mediated pathogen transmission pathways, including water, hands, soil, food and flies, will enable us to understand which of these pathways are successfully broken by our interventions and elucidate the factors behind their success or failure in improving child development outcomes. Detailed assessment of contamination along these pathways (including measurement of fecal indicator bacteria, microbial source tracking to differentiate between human and animal sources of contamination, and detection of common diarrheagenic pathogens such as pathogenic E. coli, Shigella and rotavirus using culture-based and molecular techniques) will allow a nuanced understanding of the impact of the interventions on disease transmission in young children in the rural Bangladeshi setting. In-depth information on environmental contamination will also allow us to explore the relationship between environmental enteropathy in children and fecal contamination in their living environment.

Spillovers of WASH Interventions
While there is a rich literature on the health effects of WASH interventions for children receiving such interventions, to our knowledge, no studies have measured the health effects of such interventions on children that are geographically proximate to WASH intervention recipients who did not receive the intervention themselves. Effects of interventions on those not receiving interventions are termed “spillovers,” and failing to account for spillovers in the same direction as the effect on the treated (“positive spillover”) will lead to underestimates of the efficacy and cost effectiveness of an intervention. As such, measurement of spillovers is important for the prioritization of interventions and allocation of public funds. Although many studies have applied mathematical models to spillovers of infectious diseases, very few studies have empirically measured spillovers for infectious diseases (Anderson & May 1992; Anderson & Medley 1985; Medley et al. 1993; Basáñez et al., 2012; Chan et al, 1994; Magalhães et al., 2011; Halloran et al., 2002; Bansal et al., 2006; O'Brien et al., 2007). Our proposed study will be one of the first to do so, and it will be the first to generate empirical spillover estimates for WASH interventions. This study will generate unique evidence to inform the estimation of the cost effectiveness of interventions and optimal resource allocation to maximize health benefits for rural populations in developing countries. It will concurrently advance the general methodology for spillover measurement in multiple disciplines.

Effect of the interventions on anemia
Anemia in preschool children results in poor physical growth, impaired cognitive development, reduced school achievement and, when severe, may result in increased mortality risk. The prevalence of preschool

child anemia (hemoglobin concentration<110 g/L) has been estimated at 64% in Bangladesh. While it is recognized that anemia is a multifactoral disorder, the relative contribution to anemia from micronutrient deficiency, infection, and hemoglobinopathies among low-income populations has not been well characterized. It has been assumed that more than half of the burden of anemia can be attributed to iron deficiency, yet the interplay between undernutrition and infection may reveal a more complicated story. Although iron supplementation interventions have had modest success at reducing anemia risk, high rates remain even after supplementation. This may be due to a failure to address the clinical and sub-clinical infectious causes of anemia. Certain parasitic or other enteric infections such as diarrheal disease may also contribute to anemia due to blood loss or inflammation. It has been hypothesized that environmental enteropathy (EE) is one such condition in which repeated often subclinical enteric infection, thought to be due to poor water, sanitation, and hygiene, results in a chronic state of gut inflammation and nutrient malabsorption that may contribute to anemia (Prendergast et al, 2012). No interventions designed to both improve nutrition and reduce infection simultaneously have rigorously evaluated the impact on anemia. The trial's randomized, factorial design will enable us to experimentally measure the independent and combined effects of interventions to reduce infection and undernutrition in young children. The study's findings will contribute to the critical evidence gap regarding the causes of anemia and interventions to prevent it. The results of this study are likely to be broadly applicable to other rural, low income populations where food insecurity, poor access to safe water, and inadequate sanitation coexist and could identify new strategies to address this important and intractable problem.

3. Collaborative Research

a) If any non-UCB institutions or individuals are engaged in the research, explain here.

The following collaborating institutions are are also engaged in the research, in that they will be involved in obtaining consent and collecting data data through contact with human subjects:

The International Centre for Diarrheal Disease Research, Bangladesh. This organization will employ the staff that will engage in the field work and have contact with human subjects. They will work with the raw data that includes personal identifying data. Mahbubur Rahman is a Public Health Specialist at ICDDR,B who is responsible for managing field logistics for this study.

Steve Luby from Stanford University will coordinate collaborators across institutions, provide input on intervention, protocol, and questionnaire development, and contribute to analysis and manuscript writing responsibilities. Mark Davis at Stanford will contribute to the immunological analysis in the environmental enteropathy subset.

Collaborators at UC Davis will support the development of the nutrition intervention, questions related to nutrition intervention, and uptake measures related to nutrition.

Collaborators at Emory University will provide input on measurement and will help to ensure that the Bangladesh protocol is aligned with the WASH Benefits Kenya protocol.

Michael Kremer from Innovations for Poverty Action will provide input on measurement and will help to ensure that the Bangladesh protocol is aligned with the WASH Benefits Kenya protocol.

Collaborators from Emory University and Innovations for Poverty Action are not directly engaged in the

---

Protocol Title: Measuring the benefits of sanitation, water quality, handwashing and nutrition interventions for improving health and development in rural Bangladesh

Protocol Status: APPROVED

Date Submitted: 08/18/2014

Approval Period: 09/16/2014-11/03/2016

Important Note: **This Print View may not reflect all comments and contingencies for approval. Please check the comments section of the online protocol.
Questions that appear to not have been answered may not have been required for this submission. Please see the system application for more details.**

Collaborators from Emory University and Innovations for Poverty Action are not directly engaged in the proposed research but will serve in an advisory role.

Wagner College. Dr. Mohammed Alauddin will contribute to the environmental enteropathy portion of the study. He is an Analytical Chemist, and he will measure one of the important biomarkers of EE, namely Lactoluse-Mannitol (LM) ratio in urine collected from children in the WASH Benefits study. Dr. Alauddin will employ highly sensitive technique of high performance liquid chromatography and mass spectrometry (LC-MS/MS) for the urinary analysis of lactoluse and mannitol in children. The analysis will be carried out in Dr. Alauddin’s research laboratory at Wagner College backed by appropriate method validation and quality control check. In addition to the LM analysis in urine samples, Dr. Alauddin will collaborate with the UC Berkeley team and the ICDDRB team in Bangladesh in the data analysis, interpretation, report writing and successful completion of the project. He will have access to identifiable data.

VitMin Lab. Dr. Juergen Erhardt will conduct micronutrient and acute phase protein panel analyses of blood samples as part of the nutritional assessment. Dr. Erhardt will not have access to identifiable information. He is not involved in the study design.

b) If any non-UCB institutions or individuals are collaborating in the research, complete the table below and attach any relevant IRB approvals in the Attachments section.

Non-UCB institutions

Institution Name	Individual Contact/ Affiliate of Institution	FWA #	Local IRB Review? (Y or N)	IRB Approval Date	IRB Approval Expiration Date
UC Davis			N		
International Centre for Diarrheal Disease Research, Bangladesh	Leanne Unicomb	00001468	Y	4/9/12	2/28/15
Emory University			N		
Stanford University	Steve Luby	00000935	Y	12/17/2013	12/17/2014
Innovations for Poverty Action	Michael Kremer		N		
Wagner College	Mohammed Alauddin	None	N		
VitMin Lab	Juergen Erhardt		N		

Protocol Title: Measuring the benefits of sanitation, water quality, handwashing and nutrition interventions for improving health and development in rural Bangladesh

Protocol Status: APPROVED

Date Submitted: 08/18/2014

Approval Period: 09/16/2014-11/03/2016

Important Note: **This Print View may not reflect all comments and contingencies for approval. Please check the comments section of the online protocol. Questions that appear to not have been answered may not have been required for this submission. Please see the system application for more details.**

4. Qualifications of Study Personnel

a) Explain expertise of Principal Investigator, Student/Postdoc Investigator, Faculty Sponsor (if applicable), any Co-Investigators or other key personnel listed in the application, and how it relates to their specific roles in the study team.

John M. Colford, Jr., MD, PhD (Principal Investigator)
Dr. Colford obtained his M.D. from Johns Hopkins School of Medicine, and his Ph.D. in Epidemiology from the University of California, Berkeley (1996). He is a Professor of Epidemiology in the School of Public Health at UC Berkeley. Dr. Colford has extensive experience in and prior funding from NIH, CDC, and the USEPA for the conduct of randomized trials and other studies of drinking water and environmental health issues internationally and in the United States. Dr. Colford will ensure communication with the Gates Foundation, oversee collaboration between the University of California Berkeley and ICDDRB, oversee protocol approval process through the University of California Berkeley and assure that the project activities are aligned with the obligations to the Gates Foundation.

Benjamin Arnold, PhD (Key Personnel)
Dr. Arnold is an epidemiologist in Dr. Colford's research group. He has participated in numerous studies of water, sanitation and hygiene interventions throughout Asia, Africa and Latin America. He will contribute to the study design, development of survey instruments, data analysis and scientific publications.

Alan Hubbard, PhD (Key Personnel)
Dr. Hubbard is an Associate Professor of Biostatistics, School of Public Health, UC Berkeley. He will provide guidance for the study design and statistical analysis.

Lia Fernald, PhD (Key Personnel)
Dr. Fernald, Associate Professor of Public Health Nutrition, will lead the design and execution of the outcome measures concerned with child growth and development (a role similar to that which she currently has the on Gates/World Bank WSP Project).

Kara Nelson, PhD (Key Personnel)
Dr. Nelson is a Professor of Environmental Engineering. She will oversee the environmental microbial assessment and related data analysis activities.

Jade Benjamin Chung (Graduate Student Researcher)
Jade coordinated the pilot environmental enteropathy study in Bangladesh, and is now a doctoral student at UC Berkeley. Jade will assist with the environmental enteropathy assessment and nutrition intervention implementation for the main trial in Bangladesh.

Audrie Lin, PhD (Postdoctoral Researcher)
Ms. Lin will provide guidance on the environmental enteropathy assessment and related data analysis activities. She holds a PhD in Microbiology and has experience conducting field work in Bangladesh.

Ayse Ercumen, PhD (Postdoctoral Researcher)
Dr. Ercumen will provide guidance to the environmental microbial assessment and related data analysis

---

Protocol Title: Measuring the benefits of sanitation, water quality, handwashing and nutrition interventions for improving health and development in rural Bangladesh

Protocol Status: APPROVED

Date Submitted: 08/18/2014

Approval Period: 09/16/2014-11/03/2016

Important Note: **This Print View may not reflect all comments and contingencies for approval. Please check the comments section of the online protocol. Questions that appear to not have been answered may not have been required for this submission. Please see the system application for more details.**

activities. She holds a PhD in Epidemiology and has experience conducting field work in Bangladesh.

Co-Investigators from Stanford University
Steve Luby, MD
Dr. Luby is a Professor of Infectious Diseases at Stanford University and is a Co-Principal Investigator. He will co-lead the study in Bangladesh. He will draft the protocol, coordinate collaborators, assign project implementation, analytic, report and manuscript writing responsibilities.

Amy Pickering, PhD (Postdoctoral Researcher)
Dr. Pickering will provide guidance to the environmental microbial assessment and related data analysis activities. She holds a PhD in Environmental Engineering and has experience conducting field work in Bangladesh.

Jessica Grembi, PhD Student
Ms. Grembi will lead the microbiome analysis.

Mark Davis, PhD
Dr. will support the immunological analysis in the environmental enteropathy subset.

Researchers from ICDDR,B
Leanne Unicomb, PhD, MMed Sci (Co-Investigator)
Dr. Unicomb (epidemiologist) is a Co-Principal Investigator. She will supervise the Bangladesh based project implementation team, ensuring coordination and collaboration among research investigators, supporting project management, and drafts of the manuscript on the water treatment intervention and changing the water treatment indicators after intervention.

Tahmeed Ahmed, MBBS, PhD (Co-Investigator)
Dr. Ahmed is a pediatrician and nutritionist with broad experience conducting research on undernutrition among children in Bangladesh including collaborating with Dr. Dewey and colleagues at the University of California Davis on studies of Lipid based Nutrition Supplement in Bangladesh. Dr. Ahmed will oversee the nutrition intervention in Bangladesh, work with his team to ensure that measurements of child growth are valid, and assist in study design data interpretation and manuscript writing.

Mahbubur Rahman, MBBS
Mr. Rahman is Public Health Specialist at ICDDR,B who is responsible for managing field logistics for this study.

Abu Naser, MBBS
Mr. Naser is a Public Health Specialist at ICDDR,B who is responsible for the post-intervention assessment.

Co-investigators from UC Davis

Kathryn Dewey, PhD
Dr. Dewey is a Professor of Nutrition and Director of the Program in International and Community Nutrition at UC Davis. Dr. Dewey's research area is maternal and infant nutrition, in both affluent and low income countries. She has conducted research in the U.S., Mexico, Costa Rica, Honduras, Guatemala, Peru,

Indonesia, Thailand and Ghana. Her professional service includes extensive consultation for WHO, UNICEF, the Pan American Health Organization and the March of Dimes, and serving as President of the Society for International Nutrition Research (2000-02) and of the International Society for Research on Human Milk and Lactation (2006-2008). She currently serves as chair of the Technical Advisory Group for the Alive and Thrive Project and is a member of the Steering Committee for the WHO Multicentre Growth Reference Study and the NIH Technical Working Group on Iron and Malaria. Dr. Dewey will collaborate on the nutritional components of the study.

Christine Stewart, PhD MPH
Dr. Stewart is an Assistant Professor of Nutrition at UC Davis and her research focuses on maternal and child nutrition in low income countries. Dr. Stewart will oversee the nutrition intervention activities in Kenya, and will advise all co-investigators on the LNS intervention protocol, monitoring and nutritional outcome measurement in the study. She will also assist in study design, data interpretation and manuscript writing.

Co-intestigators from Innovations for Poverty Action

Clair Null, PhD (IPA Affliliate and Emory University)
Dr. Null is an Assistant Professor of Global Health at the Rollins School of Public Health, Emory University. Dr. Null is the Principal Investigator of the parallel study in Kenya. She received a PhD in Agricultural and Resource Economics at the University of California at Berkeley in 2009. She will assure that the activities in Bangladesh and Kenya are sufficiently
aligned so that the subsequent interventions will be sufficiently similar so that the data can be combined, and will oversee the translation of the Kenya experience to Bangladesh.

Michael Kremer, PhD (IPA Affiliate).
Dr. Kremer will lead the design and evaluation of the behavioral economics components of the study

Consultants
In addition to Co-investigators and collaborators at IPA, ICDDRB, and UC Davis, a number of other individuals are providing expert advise on various aspects of the research. These experts will not be engaged in human subjects activities related to the project. These experts include Tom Clasen (Senior Lecturer in the Department of Infectious and Tropical Diseases, London School of Hygiene & Tropical Medicine), Patricia K. Kariger (Ph.D.), Dr. Pavani Ram (Assistant Professor of Social and Preventative Medicine, University at Buffalo), Dr. Elli Leontsini (Associate Professor of International Health, Johns Hopkins University) and Dr.
Peter Winch (Professor of International Health, Johns Hopkins University).

Mohammed Alauddin, Wagner College
Dr. Alauddin graduated in Chemistry from the University of Dhaka in 1975, earned his Ph.D. in Analytical Chemistry from the University of Kentucky, USA in 1982. His research includes the application of analytical techniques in solving problems of environmental, biological and geological significance. Dr. Alauddin is actively engaged in projects dealing with women and child health issues in Bangladesh in collaboration with institutions in the USA, Australia and Bangladesh.

Juergen Erhardt, Hohenheim University
Juergen Erhardt received a PhD in human nutrition is an expert in laboratory assessments of Vitamin A and iron status.

---

b) In case of international research, describe the expertise you have, or have access to, which prepares you to conduct research in this location and/or with this subject population, including specific qualifications (e.g., relevant coursework, background, experience, training). Also, explain your knowledge of local community attitudes and cultural norms, and cultural sensitivities necessary to carry out the research. See CPHS Guidelines on Research in an International Setting.

The core team will be assisted by many persons at ICDDR,B (these persons are listed as key personnel on the IRB submission to ICDDR,B), in addition to international consultants with specific expertise and experience in nutrition, child development, sanitation, handwashing, and tropical enteropathy. All consultants will participate in the development of the intervention and training modules for their specific areas and develop field quality assurance protocols for their materials. They will not participate in activities involving contact with human subjects nor in the analysis of data that might include personal identifiers. The
consultants include Patricia Kariger, PhD (child development expert), Thomas Clasen, MSc, PhD, JD (water and sanitation expert), Pavani Ram, PhD (handwashing expert), Elli Leontsini, PhD (behavior change expert) and Peter Winch, PhD (behavior change expert).

**Protocol Title:**	Measuring the benefits of sanitation, water quality, handwashing and nutrition interventions for improving health and development in rural Bangladesh
**Protocol Status:**	APPROVED
**Date Submitted:**	08/18/2014
**Approval Period:**	**09/16/2014-11/03/2016**
**Important Note:**	**This Print View may not reflect all comments and contingencies for approval. Please check the comments section of the online protocol. Questions that appear to not have been answered may not have been required for this submission. Please see the system application for more details.**

* * * Subject Population * * *

5. Subject Population

a) Describe proposed subject population, stating age range, gender, race, ethnicity, language and literacy.

The subject population will be young children and their mothers/guardians living in approximately 3-4 areas of Bangladesh where communities meet the following study criteria:

•Rural communities
•Drinking water
  o low levels of iron (<1mg/L on average) and arsenic (<50 mg/L on average) as documented in the collaborative assessments by the Government of Bangladesh and the British Geological Survey and internal testing using Hach iron kits
  o Sources known to be frequently contaminated with fecal indicator bacteria (including shallow tubewells)
•Low levels of fully hygienic latrines coverage as indicated by the Multiple Indicator Cluster Survey
•Levels of childhood stunting greater than or equal to 30%
•That the Government of Bangladesh, international non-governmental organizations working in Bangladesh and local government authorities report that no major water, sanitation, or focused nutrition programs are currently operating or planned in the area in the next 2 years.
  o (All participating communities will remain free to engage in intervention opportunities which they see as in their best interest. If water, sanitation, hygiene or nutrition promotion activities are initiated in the study community during the course of the trial, this will complicate the inferences we can draw from these areas, but we will not drop any such communities from the analysis, but, as noted in the analytic plan, we will retain the intention to treat analysis.)

  o Specifically avoiding hoar areas, hill tracks and coastal belts (i.e. significant flood risk areas)

Target children will be unborn children of pregnant women identified by report of their last menstrual period at enrollment. Target children will age to between 17 and 24 months over the course of the study. Older siblings or neighbors (age 18 – 27 months) of the target children will also be included in the study. The subject population will include both males and females, and no one will be excluded based on their race, ethnicity, language or literacy.

b) State total (maximum) number of subjects planned for the study and how many must be recruited to obtain this sample size. Explain how number of subjects needed to answer the research question was determined.

Figure 1 (attached) provides an overview of the WASH Benefits study design (The Kenya study is described in a separate protocol). The interventions will require about 3 months from the baseline survey to deliver. The follow-up rounds are planned for 12 and 24 months after intervention delivery.

In Bangladesh, we plan to enroll 90 clusters per intervention arm, a double-sized control arm, and 7 children per cluster. Because of the risk of pregnancy loss, we will enroll 8 pregnant women per cluster.

Protocol Title: Measuring the benefits of sanitation, water quality, handwashing and nutrition interventions for improving health and development in rural Bangladesh

Protocol Status: APPROVED

Date Submitted: 08/18/2014

Approval Period: 09/16/2014-11/03/2016

Important Note: **This Print View may not reflect all comments and contingencies for approval. Please check the comments section of the online protocol. Questions that appear to not have been answered may not have been required for this submission. Please see the system application for more details.**

The control arm will include a maximum of 1,440 children and 1,440 mothers and the 6 intervention arms will include a maximum of 4,320 children and 4,320 mothers. The total number of children planned for the study is 5,040, but because of the uncertainty of pregnancy outcome our goal will be to enroll 5760 target children, 5760 older siblings/neighbors of the target children, and 5760 mothers of target children. We will also interview 540 promoters. We will collect additional samples from 1800 children aged approximately 18-27 months at baseline who live in the same compounds as target children. We will enroll 2,500 children neighboring children enrolled in the study as part of the spillover substudy. The maximum number of subjects we will enroll in the whole study is 17,500 people.

HAZ Sample Size Calculations:
Using data from the SHEWA-B assessment conducted by the icddr,b Water and Sanitation Research Group with included a large sample of rural Bangladesh, the mean (SD) HAZ among the 1,413 children < 3 years old with height data is -1.825 (1.243). The village level intra-class correlation is 0.01. There are two hypotheses of interest: (H1) comparison of individual treatment arms vs. control, and (H3) comparison of the combined nutrition+WSH arm to the nutrition arm. We have slightly more power for comparison to control for due to the double sized control arm. We identified the minimum detectable effect (MDE) for the two comparisons with > 80% power. We have assumed a one sided α of 0.05. With the current design, we will have power to detect differences of +0.15 HAZ; this is approximately half the mean effect size observed in supplemental feeding intervention studies (Dewey 2008). We have targeted a smaller effect size because we expect the impacts from water, sanitation, and handwashing interventions to be smaller than from nutritional interventions.

Diarrhea Sample Size Calculations:
In the Bangladesh SHEWA-B dataset, described above, the 48 hour period prevalence of diarrhea is 12.5%. For all calculations, we have assumed that the diarrhea prevalence in the control group will be 12.0%. For comparisons of the combined WSH arm to any single treatment arm we assume that the single treatment arm prevalence is 8.0% (a 33% relative reduction from the control). There are two hypotheses of interest: (H1) comparison of individual treatment arms vs. control, and (H2) comparison of impacts in the combined WSH arm with the individual treatment arms. We identified the minimum detectable effect (MDE) for the two comparisons with > 80% power (see Table 1). We have assumed a one sided α of 0.05.

Environmental Enteropathy Subgroup Sample Size Calculations:
We will collect serum, urine, and stool specimens from a subsample of 1,500 children in the study to measure biomarkers for environmental enteropathy. Environmental enteropathy is one of the key hypothesized mechanisms for intervention impact on child growth and development. The sample of 1,500 children will be distributed equally over four arms in the study – 375 children in each of the control arm, LNS alone arm, combined WSH arm, and the LNS+ combined WSH arm. Specimen collection will take place 3 months following baseline, at midline (1 year following baseline), and endline (2 years following baseline). We expect that only 500 of the 1,500 children in the subsample will have been born at the time of our first EE assessment. We plan to collect specimens on the entire subsample at the 1-year and 2-year follow-up measurements.

We arrived at a sample size of 1,500 children (375 children per arm) using two outcome measurements we collected in our Bangladesh environmental enteropathy pilot study, the lactulose:mannitol (L/M) ratio and

---

Protocol Title: Measuring the benefits of sanitation, water quality, handwashing and nutrition interventions for improving health and development in rural Bangladesh

Protocol Status: APPROVED

Date Submitted: 08/18/2014

Approval Period: 09/16/2014-11/03/2016

Important Note: **This Print View may not reflect all comments and contingencies for approval. Please check the comments section of the online protocol. Questions that appear to not have been answered may not have been required for this submission. Please see the system application for more details.**

Endotoxin Core Antibody (EndoCAb). In the pilot, the 119 children ranged in age from 10 to 48 months and lived in 83 villages across rural Bangladesh. Using estimates of variability in the lactulose:mannitol (L/M) ratio and Endotoxin Core Antibody (EndoCAb), we estimate that this design will have greater than 80 percent power to detect differences between groups of –0.20 SDs in the L:M ratio and –0.25 SDs in IgG EndoCAb antibodies. These detectable differences are smaller than those observed between children in poor versus improved hygiene households in the pilot study: –0.42 SDs for L:M and –0.29 SDs for EndoCAb. In these calculations, we assumed a village-level intra-class correlation of 0.05 for L:M and 0.27 for EndoCAb, and child-level intra-class correlations (for repeated measures within children) that range between 0.5 and 0.9.

Intestinal Parasite Measurement in Target Children and their peers:
At baseline, we plan to collect stool specimens and blood spot samples from children 18 – 27 months who live in the same compound as the target children (7 children per cluster; 5,040 total). At the 2-year follow-up, we will collect stool specimens and blood spot samples from target children. The purpose of the stool collection is to measure the presence and intensity of intestinal parasite infections. For the stool samples, we propose to only measure protozoan parasites in this young age group because we expect the prevalence of these organisms to be reasonably high. The eventual goal of the blood spot collection will be to analyze the samples at some point in the future for intestinal helminths and protozoans using antigen-based assays.

We estimate that these samples will be sufficient to detect a relative reduction of 18% in infection prevalence. Our power calculations assume 50% prevalence in the control arm, a village intraclass correlation of 0.14, and 71% successful stool collection and analysis (10 / 14 samples per village), which is highly conservative.

Spillover Study Sample Size Calculations:
Since spillover effects are likely smaller than the direct effects of the intervention, we will need to measure outcomes in more children per arm in the spillover study than are enrolled in the main trial. We aim to detect a relative reduction of 6% in our primary outcomes. We calculated the prevalence and intraclass correlation coefficients (ICC) for diarrhea from a WASH Benefits pilot study and soil-transmitted helminths from a study of children in rural India. In the WASH Benefits pilot, the prevalence of Ascaris and Trichuris was 7.5% and 10.4%, respectively, in households with poor hygiene and 20.7% and 13.8%, respectively, in households with good hygiene. Our sample size calculations did not focus on respiratory illness due to their higher prevalence relative to diarrhea and helminth infection. The ICCs ranged from 0.023 to 0.153, and since these are somewhat larger than ICCs reported in the literature, we expect our sample size estimates are conservative. We assumed that we would measure 10 children per cluster. Assuming 80% power and a type I error of 0.05, we calculated the required sample size for each outcome of interest, adjusting for the ICC. Given these assumptions, the spillover study plan to enroll 2,000 children in 180 clusters (1,000 children and 90 clusters per arm).

c) **If any proposed subjects are children/minors, prisoners, pregnant women, those with physical or cognitive impairments, or others who are considered vulnerable to coercion or undue influence, state rationale for their involvement.**

The proposed subjects include very young children, pregnant women, and educationally and economically disadvantaged subjects. The goal of the WASH Benefits study is to generate rigorous evidence about the

impacts of sanitation, water quality, handwashing, and nutrition interventions on child growth and development in the first years of life. There is abundant evidence aggregated over more than 325,000 children from around the world that the window for interventions to improve growth is in the first 1,000 days of life, including the 9 months before birth (Victora 2010). Meeting the study goals requires intervention in the middle of this development window. Enrolling pregnant women will ensure our ability to meet our sample size goals. The rural population that we are targeting in this study are very poor, and lack the water, sanitation, and hygiene infrastructure which we are assessing.

6. Recruitment

a) Explain how, where, when, and by whom prospective subjects will be identified/selected and approached for study participation. If researcher is subject's instructor, physician, or job supervisor, or if vulnerable subject groups will be recruited, explain what precautions will be taken to minimize potential coercion or undue influence to participate. See CPHS Guidelines on Recruitment for more information.

The intervention trial will be implemented in 5 districts of rural Bangladesh. Eligible communities will be identified through area surveys conducted by ICDDRB research assistants. The fieldwork will be implemented by local field workers recruited and supervised by the Bangladesh-based scientific team. The trained fieldworkers will travel to the eligible communities and will ask community leaders for permission to conduct research within their community. If the community leaders agree, then the team will proceed with recruitment. A local village leader will accompany the field staff during their first visit to potential study households at the time of enrollment to ensure that subjects are fully informed of the implications of study participation and to avoid any potential mistrust in the community. Fieldworkers will approach randomly selected, eligible baris (a group of 3-20 households that share a common courtyard and are usually blood relatives) within a community. Only compounds that include a pregnant woman in the first or second trimester of her pregnancy with self reported low levels or no iron problems in their drinking water, who will stay in this household for the next 24 years are identified as eligible by the team for participation. Occasionally compounds may have multiple pregnant mothers and both eligible households are listed in this case. The prospect of participation in the study will be discussed with adults in the compound, including the pregnant mother/caregiver of the target infants. If a potential respondent is not able to read the consent form, the field worker will suggest that they invite a witness to help read the form with them so that they can be certain that they agree to participate in the procedures in the study. If a witness is present, both the respondent and witness will be asked to provide a thumb print or signature. After providing time for discussion among the compound residents and verbal interest to participate, a member of the field team records the GPS coordinates at the front door of the mother’s household. These coordinates will be compiled and analyzed using ArcGIS mapping software, to identify 8 closest mothers for a cluster. A buffer region of 1 km is then excluded around this cluster, before identifying the next cluster of 8 mothers. Field teamsreturn and seek formal informed consent from the head of the selected compounds and from the pregnant mother/guardian of target infants within each cluster during enrollment.

The intervention is a cluster randomized controlled trial. Each cluster will be a group of compounds that includes at least seven eligible children. The compounds within a single cluster will be located closely enough together so that a single hygiene promoter can reach each of the participating compounds by walking. If the compounds are too dispersed for a hygiene promoter to reach all of them on foot, then they will not be enrolled in the study. More than one cluster may be enrolled in a single village but clusters within the same village will need to be separated from each other by a minimum of 1km distance between the two closest households. Each of our water sanitation and hygiene interventions have both a hardware

the two closest households. Each of our water sanitation and hygiene interventions have both a hardware component (and our nutritional intervention requires ongoing provision of nutrient supplements) as well as a software, communication component. Neighboring clusters will not receive the hardware or supplies. Moreover, water sanitation, and hygiene interventions are generally plagued with low levels of uptake and regular use. Thus we do not anticipate spillover of the intervention into neighboring clusters. Since there will be at least 1 km distance, equivalent to 15-20 minutes walking, that separates the closest point of intersection between intervention areas we do not expect direct effects of the intervention to spill over into the closest cluster. In addition, each cluster will have its own community hygiene promoter, and so we will not be asking the same hygiene promoter to deliver different messages to different households. We will have GPS location information from each participating household and as part of the analysis evaluate whether effects of uptake for impact is affected by proximity to other interventions. Although we do not expect to see spillover effects, if we do will adjust for them in the analysis.

After 8 clusters have been enrolled in a particular geographical area, these 8 compound identifications will be sent to Dr. Ben Arnold at UC Berkeley, who will block randomize and assign each cluster to receive one of the 6 intervention or to the double sized control arm.

Field staff will enroll and randomize compounds into the intervention trial over a 10-month period. The enrollment will be stopped for two months after the first 80 clusters. The implementation team will follow and roll out the interventions. This time will allow the team to improve and learn from the experience so that the next rounds are more efficient.

Children born into existing study compounds, other than those born to pregnant mothers enrolled at baseline, will also receive the same intervention as the child in the compound who was originally enrolled. This will be essential to maintain a coherent and consistent intervention within each compound. Since LNS is a more targeted (and expensive) intervention, we plan to limit LNS provision to additional children born to the same mother as target children. For children who are born into existing study compounds, we will attempt to enroll them at midline and endline and if enrolled we will measure diarrhea, anthropometry (length, weight, head circumference) and child development outcomes (when possible: children would need to be > 4 months old to administer most tests). Due to cost and logistics, we do not plan to measure environmental enteropathy biomarkers or parasitic infections in the additional enrollees. We do not plan to include the additional newborn children in our primary analysis because the exposure to intervention for this subset will be shorter than for our index children, and their inclusion would increase the variability of intervention exposure length in our population (and would make defining what constitutes the “intervention” more difficult). However, children born into existing compounds may provide additional information about the impact of our interventions on very young children who are born into cleaner environments. For the spillover substudy, we will enroll children aged 0-5 years in neighboring compounds in the combined intervention and control arms at endline.

For the spillover substudy, we will enroll children aged 0-5 years in neighboring compounds in the combined intervention and control arms at endline. Compounds will be eligible to participate in the spillover substudy if they are located in close proximity to enrolled compounds in the combined intervention and control arms and if a child 0-59 months resides there at endline. Specifically, we will use GPS information to pre-define a perimeter around the study clusters in which compounds will be eligible to enroll in the spillover substudy. This perimeter will ensure sufficient distance remains between study clusters since our aim is to measure within-cluster spillovers rather than between-cluster spillovers. The diameter of the

---

Protocol Title: Measuring the benefits of sanitation, water quality, handwashing and nutrition interventions for improving health and development in rural Bangladesh

Protocol Status: APPROVED

Date Submitted: 08/18/2014

Approval Period: 09/16/2014-11/03/2016

Important Note: This Print View may not reflect all comments and contingencies for approval. Please check the comments section of the online protocol. Questions that appear to not have been answered may not have been required for this submission. Please see the system application for more details.

perimeter will be defined through piloting. We will use satellite imagery from Google Earth to identify potential compounds that will be eligible for the spillover study. These compounds will be located within 160 meters of compounds enrolled in the main study and within the perimeter described above. Because satellite images may be outdated, during endline data collection, field staff will ground truth the identified compounds upon arriving in the cluster using GPS devices. At that time, they will also determine which compounds within 160 meters of main study compounds have children 0-5 years. Compounds with children 0-5 years will be eligible for the spillover study and will be invited to participate. They will determine which study compounds have children under 0-5 years by asking neighboring compounds if any 0-5 year old children live there using the recruitment script in the attachments section.

b) Describe any recruitment materials (e.g., letters, flyers, advertisements [note type of media/where posted], scripts for verbal recruitment, etc.) and letter of permission/cooperation from institutions, agencies or organizations where off-site subject recruitment will take place (e.g., another UC campus, clinic, school district). Attach these documents in Attachments section.

Recruitment is integrated in to the consent process. There are no separate materials just for recruitment. Trained field staff will approach the eligible household and introduce themselves and describe the study and the participant involvement should they choose to enroll. The consent documents are attached.

c) Will anyone who will be recruiting or enrolling human subjects for this research receive compensation for each subject enrolled into this protocol? If yes, please identify the individual(s) and the amount of payment (per subject and total).

No

7. Screening

a) Provide criteria for subject inclusion and exclusion. If any inclusion/exclusion criteria are based on gender, race, or ethnicity, explain rationale for restrictions.

The study will be conducted in communities in 5 districts in rural Bangladesh. These communities must meet all of the following criteria:
•Have no on-going, externally-funded projects implementing or promoting water, sanitation and hygiene technologies or behaviors
•Have no on-going, externally-funded projects implementing or promoting nutritional supplementation or promotion of specific foods based on their micronutrient content
•Have water with an iron concentration of <3milligrams/L on average
•Have water with an arsenic concentration of <50 micrograms/L on average

Compounds (within eligible communities) will be eligible to participate if they include at least one pregnant woman currently living in the compound. Within each enrolled compound, we will collect information from three types of children:

(1) Infants (target child) will be eligible to participate in the study if they are:
1. They were in utero at the baseline survey

2. Their parents/guardians are planning to stay in the study village for the next 24 months (if a mother is planning to give birth at her natal home and then return, she will still be a candidate for enrollment)

(2) Children < 36 months at baseline that are living in the compound of a target child will be eligible to participate in diarrhea measurement if:
1. They are 3 – 36 months old at the baseline survey
2. Their parents/guardians are planning to stay in the study village for the next 12 months

(3) An older sibling or older child from each enrolled bari that includes a target child will be eligible to participate in the intestinal parasite specimen measurement if:
1. They are between the ages of 18 and 27 months at baseline
2. Their parents/guardians are planning to stay in the study village for the next 12 months

There will be no exclusion criteria based on gender, race, or ethnicity.

Compounds will be eligible to participate in the spillover substudy if they are located in close proximity to enrolled compounds in the combined intervention and control arms and if a child 0-59 months resides there at endline. Specifically, we will use GPS information to pre-define a perimeter around the study clusters in which compounds will be eligible to enroll in the spillover substudy. This perimeter will ensure sufficient distance remains between study clusters since our aim is to measure within-cluster spillovers rather than between-cluster spillovers. The diameter of the perimeter will be defined through piloting. We will use satellite imagery from Google Earth to identify potential compounds that will be eligible for the spillover study. These compounds will be located within 160 meters of compounds enrolled in the main study and within the perimeter described above. Because satellite images may be outdated, field staff will ground truth the identified compounds upon arriving in the cluster using GPS devices. At that time, they will also determine which compounds within 160 meters of main study compounds have children 1-5 years. These compounds will be eligible for the spillover study.

b) If prospective subjects will be screened via tests, interviews, etc., prior to entry into the "main" study, explain how, where, when, and by whom screening will be done. NOTE: Consent must be obtained for screening procedures as well as "main" study procedures. As appropriate, either: 1) create a separate "Screening Consent Form;" or 2) include screening information within the consent form for the main study.

Field staff will assess child and household eligibility by asking caregivers the age of their children (or the approximate due date for pregnant women) when they visit the household to potentially enroll them.

8. Compensation and Costs

a)

Describe plan for compensation of subjects. If no compensation will be provided, this should be stated. If subjects will be compensated for their participation, explain in detail about the amount and methods/ terms of payment.

Include any provisions for partial payment if subject withdraws before study is complete.

When subjects are required to provide Social Security Number in order to be paid, this data must be collected separately from consent documentation. If applicable, describe security measures that will be used to protect subject confidentiality.

If non-monetary compensation (e.g., course credit, services) will be offered, explain how

No monetary compensation will be given to subjects for their participation. Participants in treatment arms will receive free sanitation hardware, handwashing hardware as part of the study, which they will retain after the completion of the study. Participants with target children in the household will additionally receive free nutritional supplements (LNS), detergent for making liquid soap and a regular supply of waterless hand sanitizer and water treatment supplies for the duration of their participation in the study.

b) **Discuss reasoning behind amount/method/terms of compensation, including appropriateness of compensation for the study population and avoiding undue influence to participate.**

In rural villages in Bangladesh, the standard wage is $1.50 per day. Thus, even modest compensation risks being coercive. ICDDRB's practice, consistent with the practice of other research organizations in Bangladesh, is to provide modest tangible benefits to participants in studies (for example the water treatment supplies included in some arms of this project) and then provide potential study participants with the clear option to participate or not.

c) **Costs to Subjects. If applicable, describe any costs/charges which subjects or their insurance carriers will be expected to pay. (If there are no costs to subjects or their insurers, this should be stated.)**

Participation in the study will not result in any direct costs to subjects or their insurers, other than cases in which subjects elect to devote their own time to improving their water quality, sanitation or hygiene practices, as encouraged and facilitated by the interventions.

---

* * * Study Procedures, Alternatives to Participation * * *

9. Study Procedures

a) Describe in chronological order of events how the research will be conducted, providing information about all study procedures (e.g., all interventions/interactions with subjects, data collection procedures etc.), including follow-up procedures. If any interviews, questionnaires, surveys, or focus groups will be conducted for the study, explain and attach one copy each of all study instruments (standard and/or non-standard) in the Attachments section.

Please see Figure 1 (attached) for the overall chronology of the study. The interventions will require about 3 months from the baseline to implementation. The follow-up rounds are planned for 12 and 24 months after intervention delivery. The major activities involved in the study include:

1)Promoter Selection and Training
2)Baseline Assessment
3)Intervention Implementation
4)Midline Assessment
5)Endline Assessment

1) Promoter Selection and Training
The fieldwork will be implemented by local field workers recruited and supervised by the Bangladesh-based scientific team. For all study arms, local promoters will be nominated by community members participating in the study. Following the baseline survey icddr,b field staff will search for potential candidates by interviewing community members within each cluster. During interviews, they will describe promoter eligibility criteria and explain the role of the promoter in the community. Field staff will seek nominations from at least one member of each target household and at least two community leaders (includes school teachers, religious leaders, village doctors, NGO workers, and others) for a few candidates to work as a promoter in their community. Based on the nominations from the community, field staff will tally marks against each candidate on a checklist. Staff will identify the 3 nominated candidates with the highest scores and then they will visit them to inform the eligibility criteria, promoter roles and responsibilities, and benefits of being a promoter. Based on the discussion, if the candidate shows interest in being a promoter then field staff will proceed in collecting detailed socio-demographic information otherwise they will stop the interview and proceed to the next candidate. Following this, field staff with the support of other team members will analyze the data to identify eligible candidates for interviews. After randomization, the team will invite at least 3 preliminary eligible candidates from each intervention cluster for interview (both written and viva). Based on the performance of the interview one will be chosen as the promoter while other will be in the waiting list as a back-up promoter in case the selected promoter is unable to satisfactorily carry out his/her responsibilities or drop out due to any reasons.

All selected promoters will be invited for 2 -day basic training conducted by icddr,b staff which includes research and training team on interpersonal communication, introduction to behavior change communication strategies, basic adult learning theory, time management/planning and reporting producers. Upon successful completion of the initial training, and based on the trainer's assessments of the two nominees from each village, one will be chosen as the promoter. The selected promoter of individual arm will subsequently attend a two-day training specific to the intervention they will be promoting

individual arm will subsequently attend a two-day training specific to the intervention they will be promoting and, if the cluster is randomized to the combined arm, an additional 4 days of training. For example, promoters in nutrition arms will address the importance of good nutrition for child development, the basics of a healthy diet for children in the target age range, the LNS product and nutrition specific health education modules that have been specifically developed for this project. Field staff along with the training team will assess the performance (knowledge, skill and attitude) of promoter in each quarter by using performance assessment tools and will do the grading according to their performance. Based on the assessment there will be refresher trainings at every quarter that will last for one to two days and present a review of general themes as well as any new behavior change communication strategies that have been developed. The refresher training will serve as a venue to share ideas, lessons learned and best practices among the promoters and their supervisors.

Shortly after the training workshops, community meetings will be conducted to introduce the intervention and to present the promoter and describe their role to the community and mothers participating in the study by a representative from icddr,b. The study representative will then accompany the promoter on his/her first 2-3 participant interactions (lasting ~1 hr each) and provide the promoter with feedback on his/her techniques. The promoter supervisors will each oversee ~12 promoters for the duration of the study. They will stay in touch with promoters through monthly meeting, monthly phone calls and site visits in each month. In order to ensure that study households are adequately supported, promoters will be asked to make frequent contact with study households in the first days and weeks after the intervention is launched, with interactions then tapering off after the first 6 months of the study to a long-term pattern of monthly visits by promoters to deliver LNS, Aquatabs, hand sanitizers and technical support on repairs as required in addition to provision of ongoing behavior change support, and check on uptake. Promoters will be compensated for their efforts at a rate that is commensurate with the government’s pay to community health workers, with a strong emphasis on the prestige of being selected as a promoter (actualized in the form of a diploma from the training, a household visit kit, and study identification badge and potentially monthly top-up for promoters cell phone to facilitate communication between research staff and promoters).

2) Baseline Assessment (age -7 to 0 mo.)

After a pregnant mother has been enrolled in the study, trained ICDDRB staff will conduct a baseline assessment. Mothers will be asked standard questions about their (and spousal) education, activities, occupation, household assets, and current sanitation and hygiene practices. The baseline assessment in all participating households will include bar soap and detergent powder consumption measurements, latrine use (visual inspection), and numerous spot-check hygiene indicators (e.g. presence of animal or human feces in the household environment). The field team will collect these measurements at the three measurement rounds of the study (baseline, 1-year follow-up, 2-year follow-up). We will ask mothers to provide a 10 mL sample of blood for future genetic testing and testing of nutrition and other biochemical parameters, a urine sample for chemistry and a stool sample to permit future testing of the intestinal microbiome. For the blood sample we will separately freeze aliquots of sera for eventual biochemical analysis, and retain a clot for genetic testing. Although we are not currently funded to analyze these samples, we envision future interest in assessing the impact and controlling in the analysis for maternal nutritional deficiencies or other biochemical factors on child development, and so we will collect and archive this prenatal sample. We are requesting consent to hold these samples for up to 20 years because we want to take advantage of expected future advances in characterizing immunological and other

---

Protocol Title: Measuring the benefits of sanitation, water quality, handwashing and nutrition interventions for improving health and development in rural Bangladesh

Protocol Status: APPROVED

Date Submitted: 08/18/2014

Approval Period: 09/16/2014-11/03/2016

Important Note: **This Print View may not reflect all comments and contingencies for approval. Please check the comments section of the online protocol. Questions that appear to not have been answered may not have been required for this submission. Please see the system application for more details.**

parameters relevant to the pathophysiology of environmental enteropathy. We have not specified the tests, because we do not know which relevant tests will become available, though the field is making substantial advances each year, and so we expect that highly relevant tests will become available.

Fly Density
In the random subset of 720 households within each group we will assess environmental contamination at baseline by measuring fly density. Field workers will start by locating the nearest latrine, food preparation area (usually rural outdoor kitchens) and the garbage disposal site for the target household. To capture flies, they will use Revenge fly tapes from Roxide inc. We chose these passive sticky tapes because of their adhesion ability. They will cut out three 1.5 ft of these tapes and hang them parallel to each other near the sites. The field staff will set these traps between 9-10 am,ask the household not to disturb the tapes, and collect them after 24 hours. Trained field workers will count the number of flies in each trap and speciate them using a simple visual identification chart made from The Fauna of British India series (Aubertin and Smart 1940; Van Emden 1965; Nandi 2002).

Parasite Assessment (compound residents aged 18 to 27 months)
Since at enrollment the target children will not yet be born, and even those who are born are at low risk of parasitic infection, we will enroll older children age 18 to 27 months who live in the same compound as the target child as a proxy to assess the risk for parasitic infection in our target communities. These children will be the same age at baseline as the target will be at endline. Stool and blood spot samples will be collected from these children. Stool collection will require two visits to each household. On day 1, the field team conducting the survey will deliver to each caregiver a stool collection kit and instruct them how to collect stool from their children. Caregivers will be instructed to have their child defecate on a sheet of provided plastic, to use a provided plastic scoop (integrated into a storage container) to collect ~10 mL of fresh stool from the top of the pile. On day 2, field staff will return to the household to collect the stool sample. Field staff will aliquot fresh stool specimens for parasite microscopy at endline. We will maintain a cold chain of 4°C until the samples are transported to the field lab (<6 hours) where they will be stored at –20°C until shipment on dry ice to ICDDRB where they will be stored at final temperature of -80°C.

Paired with each stool sample we collect we will also collect a finger prick blood sample. One of the child's fingers will be cleaned using the disinfectant liquid and after drying completely prick to adapt to 0.25 mm using a spring-loaded disposable handset (BD Microtainer®). Six drops of blood (about 60 µl) will be collected using a filter disk. We will store the filter disk samples at 4°C. The filter disk will then be frozen and transported to the ICDDR,B lab, where they will be stored at –80°C. The eventual goal of the filter disk blood specimen collection will be to analyze them for intestinal helminthes, protozoans, and other pathogenic organisms using antigen-based assays (Luminex), but this protocol does not include the filter disk analysis activities. At endline, we will measure the study child's hemoglobin concentration using Hemocue analyzers (Hemocue 301). Results will be provided to the household in the field.

Subsample Environmental Enteropathy (EE) Assessment (6 months after baseline, midline at 15 months and endline at 27 months after baseline)
We will randomly select 80 clusters from the control group, 80 clusters from the combined water,

sanitation, hygiene (WASH) group, 80 clusters from the nutrition group, and 54 clusters from the nutrition plus WASH group for markers of environmental enteropathy. This sample collection will be implemented over the course of 2 days per village at the household level. We plan to collect samples from 1500 infants at the first survey round, which will take place approximately 6 months after baseline (however, 1500 infants is an optimistic number as a significant fraction of mothers may be living in their parents' village during the first few months of their child's life or a fraction of infants will still be in utero and will thus be unavailable for sample collection). However, the entire sample of 1,500 will likely not be be present for the 1-year (midline) and 2-year (endline) assessments. Thus, at the midline and endline assessments, we will need to increase the sample size to 2,000 children. Each round of assessment will include the urine, serum, and stool collection described below. Furthermore, at the midline and endline assessments, we will collect blood, urine, saliva, hair, and stool specimens from a subsample of 2,000 children in the study to measure biomarkers for environmental enteropathy, allostatic load, and telomere length. Additionally, we will collect blood, saliva, hair, and urine from the 2,000 mothers of the children in the EE subsample.

On the first day, consent, anthropometric measurements, blood pressure, heart rate, autonomic function, and skin conductance (sweat) measurements, provision of stool collection materials, and saliva, hair, and blood collection take place. On the second day we will collect urine and additional saliva samples from the study children and their mothers, pick up the stool from the study children, interview the mothers about their diets as well as their infant's diet during the past week and the previous 24 hours. On a non-consecutive day, a subsample of mothers (n=60) will be revisited to be interviewed about their diet as a quality control measure.

Saliva sampling:

Saliva and hair sampling, autonomic function testing:

On day 1, the field team will collect a total of 3 saliva specimens each from mothers and their children (before the blood draw, immediately following the blood draw, and at a later time point after the blood draw) using the previously approved Salimetrics swab. They will place the Salimetrics swab under the child's tongue for 2-3 minutes and store the swab in a tube. These Salimetrics samples will be used to measure salivary cortisol reactivity and cytokine response before, during, and after an acute minor stressor (the blood draw). A hair sample consisting of 3-4 strands of hair from each mother and child will also be taken with stainless steel scissors. Three to four strands of hair will be cut from three or four locations on the head. These hair samples will be used to measure hair cortisol and long term stress. To measure allostatic load, blood pressure measurements will be taken using a standard sphygmomanometer, heart rate and autonomic function will be measured using a portable electrocardiogram (Biopac ECG), and skin conductance (changes in sweat response, a test of the autonomic nervous system) will be measured using a galvanic skin response meter (MindWare GSR). The ECG and GSR will be attached before, during and after the acute minor stressor (the blood draw), to correlate autonomic function with cortisol activity. The mother will receive the blood pressure and heart rate results for herself and her child. The saliva and hair samples collected from day 1 will be used to measure cortisol reactivity, cortisol recovery, and cumulative cortisol levels using a commercial ELISA kit. On day 2 in the morning, the staff will collect an additional saliva sample from the child and the mother using the Oragene kit which consists of a soft sponge and tube. Telomere length will be measured in saliva samples collected from the children and mothers on day 2. On day 2 in the afternoon, field workers will collect samples using the Oracal sampling workers will

---

Protocol Title: Measuring the benefits of sanitation, water quality, handwashing and nutrition interventions for improving health and development in rural Bangladesh

Protocol Status: APPROVED

Date Submitted: 08/18/2014

Approval Period: **09/16/2014-11/03/2016**

Important Note: **This Print View may not reflect all comments and contingencies for approval. Please check the comments section of the online protocol. Questions that appear to not have been answered may not have been required for this submission. Please see the system application for more details.**

collect samples using the Oracol sampling tube. Specifically they will 1) Remove the sampler being careful to touch only the handle. 2) Holding the handle as a tooth brush. Insert it in child's mouth and the rub sponge against the gums for one to two minutes, ensuring that the sponge is completely wet/saturated. 3) Without touching the sponge, insert the sampler into the original Oracol tube, sponge down 4) Close the lid tightly 5) Put the Oracol sampler tube into a storage bag for transfer to a cooler. The Oracol device is used to collect crevicular fluid to study pathogen-specific antibodies. These Oracol samples will be assessed using new assays to measure pathogen specific IgA and IgG antibodies and compared to serological markers.

To measure telomere lengths, DNA will be extracted by using a commercial kit (QIAamp. We will use a validated published method to measure relative telomere lengths by quantitative PCR (Cawthon, 2002). Briefly, this method determines relative telomere lengths by measuring the factor by which each DNA sample differs from a reference DNA sample in its ratio of telomere repeat copy number (T) to single copy gene copy number (S) (Cawthon, 2002). The T/S ratio is proportional to the average telomere length. To convert the T/S ratio into base pairs, we will use a formula derived from the mean telomeric restriction fragment length from Southern blot analysis and the slope of the plot of mean telomeric restriction fragment length versus T/S (Entringer, 2011).

Urine specimen collection and analysis: From a subset of every fifth cluster (n=144), urine will be collected from the pregnant mother for micronutrient and iodine testing. These will be maintained at 4 degrees C at the field level and stored at -20 degree C at the field office.

Our field teams will collect urine samples from all eligible children and their mothers in a study cluster (up to 7 children) in one day per cluster. The field team will request the mother to collect a sample from her first urine of the day. Additionally, the field team will request that mothers not feed their children for at least one hour before they receive the lactulose-mannitol solution. The children will be weighed and measured using the same anthropometric procedures as described above. The lactulose-mannitol solution will be prepared at the ICDDR,B nutritional biochemistry lab using lactulose syrup and mannitol powder secured from international pharmaceutical suppliers. The lactulose-mannitol solution will be mixed with sterile water to produce a solution with a concentration of 250 mg of lactulose and 50 mg of mannitol per milliliter. The lactulose-mannitol assay requires the collection of an additional pre-LM urine sample to serve as a "control or baseline" urine for comparison with the post-LM urine. A pre-LM urine sample is a sample of urine (12 ml) that is collected during the 1-hour fasting period preceding the administration of lactulose-mannitol solution to the child. This additional pre-LM urine sample does not change the amount of time we are present in the household. For assay standardization and QA/QC purposes, we also plan to spike these pre-LM urine samples with fixed concentrations of lactulose, mannitol, or a known interfering compound during analysis.

Field workers will administer 2 ml of the solution per kilogram of body weight of the child. A urine collection bag equipped with a drainage tube will be attached to the infant immediately after dosing. Thirty minutes after the infant consumes the sugar solution, mothers will be encouraged to breastfeed infants <6 months or offer water to their children >= 6 months to help their urination. Children over 6 months will be given purified drinking water 30 minutes after taking the sugar to help urination. Whenever the child urinates, the urine will be removed from the bag and placed in a container with 0.1% thimerosal (1 drop per 5 ml), a preservative. The total volume of urine collected after 5 hours will be noted, a 12ml well mixed sample will be stored at -80 degrees C, and the urine bag will be removed from the child.

Since the mannitol/lactulose concentration measurements necessitate the use of high performance liquid chromatography and mass spectrometry (LC-MS/MS), which is presently unavailable at icddr,b, the child urine samples will be shipped to the US and also analyzed in Bangladesh, where the LC-MS/MS labs are located. We plan to collaborate with Dr. Mohammad Alauddin and his team at Wagner College to analyze these urine samples. Dr. Alauddin has a mass spec machine at his lab in Wagner College that will need to be calibrated and standardized with the Pre-LM urine samples before he ships the mass spec machine to his lab in Dhaka, where the remaining urine samples will be analyzed. Oxidative stress and hypothalamic-pituitary-adrenal axis markers will also be measured in the urine samples from the mother and child: F2-isoprostanes will be analyzed using gas chromatography (GC)/negative ion chemical ionization (NICI) mass spectrometry (MS) (Morrow and Roberts 2002), 8-hydroxy-2'-deoxyguanosine (8-OHdG) will be measured in using high-performance liquid chromatography-tandem mass spectrometry (HPLC-MS/MS) (Weimann, Belling et al. 2002), and catecholamines (epinephrine, norepinephrine, and dopamine) will be measured using a commercial ELISA kit (Parks, Miller et al. 2009).
The urine samples will be analyzed for concentrations of mannitol and lactulose using high performance ion chromatography at the ICDDRB laboratory (Barboza 1999).

The urine samples will be analyzed for concentrations of mannitol and lactulose using high performance ion chromatography at the ICDDRB laboratory (Barboza 1999).

Venous blood specimen collection and analysis: Before the urine specimens are collected, trained phlebotomists will collect up to 7.7 ml of venous blood from each child (< 2.5% of total blood volume for infants > 2 kg). and 10 ml of venous blood from each mother. We plan to collect an additional 2.7 mL of blood for serum micronutrient biomarker analysis during endline and we believe that this additional blood volume are not physiologically significant since the children are around two years old during endline. Retinol binding protein, transferrin receptor, ferritin, hepcidin, folate and B12, c-reactive protein, and alpha-1 acid glycoprotein will be measured in these serum samples. The additional sample collection tube is required due to the fact that a number of these assays do not perform well in samples collected in trace element-free plasma collection tubes and to ensure that there is sufficient sample volume to meet the assay requirements. With this added tube, the total blood volume to be collected from children at endline will be 7.7 mL. Blood samples will be centrifuged within three hours of collection to separate the plasma and serum from the red blood cells. The plasma and serum will then be stored at –80°C. Commercially available ELISA kits will be used to measure total IgG, IgG endotoxin core antibodies (Coaset EndoCAb) and C-reactive protein, alpha-1 acid glycoprotein (AGP), interleukin 6 (IL-6), interleukin 1 (IL-1), tumor necrosis factor (TNF), insulin-like growth factor 1 (IGF-1), and other environmental enteropathy biomarkers. Maternal blood samples will be assayed for inflammation and stress biomarkers. Blood spots will be collected for luminex testing of antibodies to intestinal parasites. At least 50 microliters of each sample will be reserved for nutritional markers (iron, vitamin A, B12, folate) and the rest of the sample will be frozen to allow for the analysis of infection with enteric pathogens and environmental enteropathy biomarkers. Aliquots of the blood samples will be shipped to Mark Davis's lab at Stanford University (USA) for immunological analyses using CyTOF, a Time-of-Flight mass spectrometer to measure highly multi-parametric single cell data including cytokine panels and peripheral blood phenotyping. Aliquots of the blood will also be shipped to Hohenheim University (Germany) for micronutrient and acute phase protein panel analyses in Juergen Erhardt's lab.

Stool specimen collection: The field team will collect stool samples from all eligible children in the study cluster. Stool collection will require two visits to each household. On day 1, the field team will deliver to

each caregiver a stool collection kit and instruct them how to collect stool from their children. Caregivers will be instructed to collect stool from their children on the following morning in the event that the child defecates before they report to a central location in the cluster for urine and serum sample collection. Caregivers will be instructed to have their child defecate in a plastic, non-absorbent diaper and to use a provided plastic scoop (integrated into a storage container) to collect ~10 mL of fresh stool from the top of the pile. On day 2, field staff will visit the households for the urine sample collections in each cluster (details above). Field staff will aliquot stool specimens (either collected by the caregiver in the early morning, or collected by field staff on Day 2) into 5 cryovials and maintain a cold chain of -20°C until the samples are transported to ICDDR,B (<6 hrs) where they will be stored at –80°C until they are analyzed using qPCR to identify Entamoeba histolytica, Giardia and Cryptosporidium. The remaining 4 aliquots will be retained for testing stool markers of environmental enteropathy and to assess the fecal microbiota and microbiome. At endline, the remaining fresh stool will be used to measure soil transmitted helminthes (as outlined in the endline parasite assessment).

Quality Assurance / Quality Control: We will include biological and technical replicates to ensure data validity. Aliquots from the same biological or environmental sample will be analyzed separately and compared. We will include negative controls daily for water testing. For example, the ELISA test can be performed twice on two separate days for the same sample. We will set aside an aliquot of each batch of L/M solution for further testing in case there are any batch inconsistencies. Multiple field research assistants can record anthropometric, blood pressure, and heart rate measurements for the same child to measure human error. Two percent of questionnaire assessments will be repeated by the supervisor within 7 days of data collection

A list of the modules/instruments that will be utilized at baseline include:

Module 2. Diarrhea and illness symptoms
Module 7. Handwashing assessment
Module 8. Sanitation assessment
Module 9. Child defecation and feces disposal assessment
Module 10. Water treatment, storage, and quality assessment
Module 14. Environmental enteropathy assessment (subset of study population)
Module 15. Intestinal parasites assessment (for older siblings of target children)
Module 18. Quantitative fly assessment (subset of households)
Module 20. Behavioral determinants
Module 22. Household food insecurity

3) Intervention Implementation

The interventions arms to be implemented include:

1) Improved water quality: chorine tablets (Aquatabs) + 5 liter safe storage vessels, water treatment promotion

2) Improved sanitation: sanitation promotion, child potties, sani-scoop hoes to remove feces from household environments, latrine upgrades to dual pit latrines

3) Improved handwashing: promotion of handwashing with soap or waterless hand sanitizers at critical times, handwashing stations, soapy water at handwashing locations. Specifically, for the kitchen a 16 liter bucket with tap fitting, stool, bowl and soapy water bottle will be provided. For the latrine, a 40 liter bucket with tap fitting, stool, bowl and soapy water bottle will be provided.

4) Combined water + sanitation + handwashing: this combined arm includes all interventions described above in #1, 2, & 3, with phased implementation

5)Nutrition supplementation: the nutrition supplement we will use provides a combination of energy and micronutrients delivered in 10 gram sachets (produced by Nutriset), to be mixed into existing meals (i.e. porridge) two times per day for target children age 6 mo. to 24 months in age. The supplement provides 108 kcal/day and includes a broad suite of essential fatty acids and micronutrients. The nutrition supplement is meant to supplement breastfeeding and locally available complementary foods. Additional messages about breast feeding and the consumption of micronutrient-rich complementary foods modeled on those recommended in the Guiding Principles for Complementary Feeding of the Breastfed Child [Dewey 2003] and the recent UNICEF Program Guide for Infant and Young Child Feeding Practices [Unicef 2011]

6) Nutrition supplementation + water + sanitation + handwashing: this arm will include all of the sub components described above, with phased implementation

The behavior change strategy and communication plan is attached in a separate document.

Two additional arms will be the control group.

Upon completion of the baseline data collection in 8 clusters, the research investigator overseeing site selection (Sania Ashraf) will compile the list of 8 clusters with their cluster IDs drawn from the database and listed in the order that the baseline data collection aws completed. An external research investigator (Swapon Biswas) will email the cluster IDs to a second offsite co-investigator (Ben Arnold). The offsite co-investigator will use prespecified statistical code to generate blocks of 8 assignments in random order specifying each of the 6 potential interventions (improved water quality; improved sanitation; improved handwashing; combined water, handwashing and sanitation; nutrition; nutrition plus improved hand washing; combined water, handwashing and sanitation) plus two assignments to the control group. He will return a password protected WASH B Bangladesh Treatment Assignment spreadsheet to Mahbubur Rahman, senior program manager for this study at icddr,b. It will be stored on a secure server that is backed up regularly. He and the four investigators in charge of delivering the interventions will be the only people who have access to the file to prevent unblinding the primary investigators and primary data analysts for the trial.

We will hold separate training sessions for community promoters who are delivering different interventions. Thus, when we convene a training session for community promoters who will be implementing the water quality intervention, there will be no training on handwashing promotion. This will reduce the risk of spillover of intervention from one intervention group to another. However, it also requires that enough clusters be enrolled and community promoters identified to be able to convene an intervention specific training.

In order to accumulate sufficient intervention-specific promoters for training there will be a period of up to 3 months between the baseline assessment and the intervention implementation. Field staff will return to the intervention compounds after the baseline assessment and randomization into intervention arms. ICDDRB staff and the community hygiene promoters will distribute handwashing stations, potties, sani scoops, provide Aquatabs and safe water storage containers, and/or LNS packets, depending on the study arm. The intervention team will work in collaboration with the Village Education Resources Center (VERC), who has considerable experience installing dual pit latrines in rural communities in Bangladesh, to install dual pit latrines in eligible compounds as determined from baseline data. Field staff will discuss the process of intervention implementation and messaging with study participants in each community as the interventions are being rolled out.

At the commencement of intervention roll out, community meetings will be held including community leaders to explain the study and the interventions. A trained, local female health promoter will deliver the behavior change messaging and will promote intervention use. The hygiene promoter will work with authority figures in the community to communicate messages depending on the assigned intervention, for example that child feces should be disposed in a latrine, that it is the occasional unseen contaminant in both water and on hands that needs to be protected against, and that key times to wash hands with soap include after defecation, after cleaning a child who has defecated, before preparing food and before eating or feeding their infants. Promoters will engage in a conversation with participants. They will observe household and compound conditions and personalize placement and use of enabling technologies, listen to people's concerns work with them to solve problems, respond to questions, encourage household members to use the enabling hardware and products in their presence to ensure understanding of use, and encourage and congratulate the adopters. Intervention promoters will also collect a subset of the indicators (LNS sachet consumption, hardware use indicators) on a monthly basis.

The nutrition intervention will be implemented in two study arms. During the first 6 months of life, promoters will encourage mothers to exclusively breast feed their children. When children turn 6 months of age and are starting to eat solid foods, community promoters will instruct mothers/guardians to continue breastfeeding along with offering solid food. Promoters will instruct mother/guardians to mix 1 sachet (10 mg) of the supplied nutrition supplement (LNS) and either feed it directly to the child or mix it with rice or other food fed to the child 2 times per day. The mother/guardian will be given a 1-month supply of the supplement at a time. In the event that the specified LNS is not immediately available at the study start-up, we will initiate the trial with Nutributter, an off-the-shelf product that is very similar in nutrient content to our research formulation of LNS (Appendix 8), and is available through a second factory in the United States (Edesia). The local health promoter will deliver the monthly supplies, will be responsible for delivering behavior change communication (BCC) messages encouraging continued breastfeeding, feeding of nutrient rich complementary foods, feeding frequency, and proper use of the supplement. The behavior change communication messages will follow the best practices for complementary feeding interventions specified by WHO and Unicef and utilize the recommendations and practices from the Alive and Thrive program in Bangladesh (Dewey 2003, Unicef 2011).

For all intervention arms, the hygiene promoter will visit the participating household frequently (i.e. 1 visit per week) early in the study; later in the study, the promoter visits will taper off to one visit per month.

If at any visit to an intervention household a community hygiene promoter identifies a serious illness or injury that she believes is related to the intervention (e.g. an injury associated with construction of a new pit

latrine) then the hygiene promoter will inform her supervising field research assistant who will record the details and notify Dr. Md. Mahbubur Rahman.

4) In-Depth Environmental Assessment in Sanitation, Combined WASH and Control Arms

In order to assess the impact of sanitation improvements alone and in combination with water and hand hygiene interventions on fecal bacterial commination in the household environment, we will collect environmental samples from all households enrolled in the sanitation arm and the combined water, sanitation, and hygiene arm, and half of the households in the double-sized control arm. This environmental assessment will occur between 2 -10 months post intervention delivery, preceding the 1-year midline assessment. We will measure the level of fecal contamination along five transmission pathways, including water, hand, soil, food, and flies among 2160 households (720 households per arm). Each enrolled household will be visited twice, as discussed below (consent form in Appendix 1q).

On the first visit, we will collect the following samples for microbial analysis: hand rinse from target child, soil from the child's play area, stored drinking water, source drinking water, pond water, food to be served to the target child, and flies captured near the food preparation area. Field microbiologists will collect 250 mL of water samples in sterile Whirlpak bags from the household's tubewell as well as from drinking water storage containers, by asking participants to provide a glass of water that they would give to their child to drink. Index child hand samples will be collected by rinsing the hands of the index child, one at a time, in 200 mL of sterile water in a sterile Whirlpak bag (Pickering et al., 2010). In each study compound, ~100 grams of soil will be excavated using a disposable sterile scoop from approximately 20cm by 20cm area where the index child is currently playing or reported to most recently have played (Pickering et al., 2012). In Bangladesh food is usually prepared in the morning, then fed to children throughout the day. Any previously prepared food being stored in the household for consumption by the index child will be sampled by collecting ~25-50g directly from the storage pot using a sterile spoon, then placed into a sterile plastic bag. Flies will be captured using sticky tape or baited traps placed near the food preparation area at the beginning of the household visit.

All water, hand rinse, food, fly, and soil samples will be placed on ice and transported to the WASH Benefits field laboratory for quantitative analysis for E. coli and fecal coliforms within 8 hours by the Colilert most probable number method (IDEXX) (Eckner, 1998). Lab technicians will analyze water and hand rinse samples directly. They will analyze soil samples by first homogenizing the sample, then suspending and agitating a specified amount (5-20g) of the soil in sterile water for subsequent processing by the IDEXX method. Food samples will be processed by mixing an aliquot of ~25g with 100ml 0.1% peptone water (Islam et al., 2012). Flies (up to 5) will be removed from the traps with a sterile tweezer, placed in a sterile tube with 1ml sterile saline solution, crushed with a sterile pestle, then further diluted with saline solution for processing by IDEXX. Serial dilutions will be prepared as needed for pond, food, fly, and soil samples. One duplicate and one blank of sterile water will be analyzed for every 10th sample.

In addition to IDEXX analysis for E. coli and fecal coliforms, aliquots from all food and fly homogenized samples (mixed with sterile water) will be processed by membrane filtration and subsequently cultered for the presence of shigella and enterobacteriaceae in the field lab. For these same food and fly samples, IDEXX wells that are positive for E. coli post-incubation will be lanced and the contents removed and centrifuged to isolate E. coli cells. These cells will be frozen and transported to the icddr,b laboratory for DNA extraction and molecular analysis by multiplex PCR of the following pathogenic E.coli genes: eae, ial, bfp, ipaH, st, lt, aat, aaiC, stx1, stx2.

An aliquot of each soil sample will also be processed by Kato Katz microscopy for detection of Ascaris, hookworm, and Trichuris ova (Albonico et al., 2012)- the most common soil transmitted helminth infections in Bangladesh affecting children. In addition, aliquots from a subset of hand rinse, soil rinse, diluted food, and water samples (total of ~2000 samples) will be vacuum filtered and archived for subsequent DNA/RNA extraction, molecular fecal source tracking analysis (to differentiate fecal contamination of animal vs. human origin), and detection of two of the most common child diarrheal pathogens in Bangladesh: rotavirus and pathogenic E. coli. The archived filters (n~2000) will be transported to Stanford University for molecular analysis.

During the first household visit for in-depth environmental assessment, we will also administer a brief interview to the primary caregiver of the index child to assess caregiver reported diarrhea for the index child and other children < 60 months living in the compound. This will be followed by a second visit where we will administer the same questionnaire about children's health. The second visit is designed to fall within a plausible incubation period between child exposure to a diarrheal pathogen and illness.

During each of the two household visits, spot checks will be conducted to assess indicators of relevant behaviors (compliance with the interventions), including water treatment, latrine usage, child and animal feces management, hand hygiene, and food hygiene. These spot checks include: latrine features (slab, functional water seal), presence and quantity of human and animal feces in compound, presence and functionality of child feces management tools and potty, presence of soap and water at handwashing stations, presence of visible dirt on caregiver and child's hands, presence of chlorine residual in stored drinking water, drinking water storage container and extraction method, presence of clean cover over stored food, presence of animals in household and animal feces in compound, and presence of flies in food preparation area and near the latrine.

5. Longitudinal Environmental Assessment in Sanitation and Control Arms

To assess the long term impact of the sanitation intervention on microbiological contamination in the household environment, we will longitudinally follow 720 householdsinthesanitation and control arms (360 households per arm) through quarterly visits over two years, for a total of eight visits per household over the study period.The visits will start approximately 12 months after intervention implementation.

Data Collection:
At each quarterly sampling round, we will collect samples from households' stored water, mother hands and children's hands for analysis of fecal indicator organisms (E. coli and fecal coliforms). First, concentrations of fecal indicator organisms in environmental samples and on hands have substantial temporal and spatial variability [Levy 2009, Pickering 2011, Ram 2011], and repeated samples have been recommended to adequately characterize contamination [Boehm 2002, Jensen 2004]. Fecal indicator detection frequency over repeated measurements has also been shown to better predict pathogen presence in tubewells than single samples [Ferguson 2012]. Additionally, we also anticipate temporal trends in fecal contamination of water and hands. Bangladesh has a dry season from November to April and a monsoon season from May to October, during which flooding is common. Seasons have a marked impact on environmental contamination in rural Bangladesh, with heavier contamination during the rainy season [Leber 2011, van Geen 2011]; wet conditions also lead to prolonged pathogen survival in the environment [Santamaria 2003]. Even within a given season we expect variation in the conditions that

---

Protocol Title: Measuring the benefits of sanitation, water quality, handwashing and nutrition interventions for improving health and development in rural Bangladesh

Protocol Status: APPROVED

Date Submitted: 08/18/2014

Approval Period: 09/16/2014-11/03/2016

Important Note: **This Print View may not reflect all comments and contingencies for approval. Please check the comments section of the online protocol. Questions that appear to not have been answered may not have been required for this submission. Please see the system application for more details.**

spread pathogens from feces into the environment; for example, the groundwater table is low during the early monsoon and equalizes with surface waters by the late monsoon [Knappett 2011]. Quarterly sampling will allow nuanced assessment of contamination during different seasons (i.e. early wet, late wet, early dry, late dry).

During two sampling rounds (quarterly visits 2 and 3), we will collect an additional aliquot of stored tubewell water, mothers' and children's hand rinse samples as well as a sample of soil from the child play area within the compound from the same 720 households to measure selected pathogens and conduct microbial source tracking. We will analyze these samples with molecular methods for three of the most common diarrheagenic pathogens in Bangladesh (rotavirus, enterotoxigenic E. coli, Shigella) [Black 1981, Albert 1999] and for the protozoa that WASH Benefits measures in child stool (Cryptosporidium sp., Giardia sp. and Entamoeba histolytica). We will also run molecular assays for general, human, ruminant, and avian unique fecal markers in these samples to identify the source of the fecal contamination as originating from humans, cattle or poultry. These source tracking assays detect bacteria that are specific to fecal hosts, such as Bacteroidales; and the identification of host-specific genes by molecular methods allows their use for microbial source tracking [Stoeckel 2007]. We will also analyze the soil samples by microscopy for the soil-transmitted helminths that WASH Benefits measures in stool (Ascaris lumbricoides, Trichuris trichuria, Ancylostoma duodenale and Necator americanus). Finally, we will analyze the soil samples collected during these two rounds for E. coli and fecal coliforms as well to aid with interpreting the pathogen data. This sampling will be done during the first year of the study period to capture the impact of the interventions during ongoing behavior change promotion and the two rounds of sampling (which will fall during the late wet and early dry seasons) will allow us to assess seasonal impacts.

Sample Processing:

Measurement of Fecal Indicator Organisms: Field microbiologists will collect 250 mL of water from household storage containers by asking participants to provide a glass of water that they would give to their child to drink. A composite soil sample will be collected from three soil locations where the child was reported to have most recently played/slept as recalled and reported by the primary caregiver. At each location, 50 g of soil will be excavated from a 30-cm by 30-cm area by scraping the soil surface until 50 g of sample has been obtained, for a total of 150 g. A separate sample of 50 g will be collected from the tubewell area adjacent to the hand pump or to the tubewell platform if one exists. Soil will be scraped into a sterile centrifuge tube with a sterile disposable plastic scoop. Target child and mother hand samples will be collected by rinsing the hands, one at a time, in 200 mL of sterile water in a sterile Whirlpak bag.

All samples will be placed on ice and transported to the ICDDR,B field laboratory for analysis for E. coli and fecal coliforms within 8 hours. Lab technicians will process aliquots of 100 mL from water and hand rinse samples following the standard IDEXX Colilert most probable number (MPN) method. Soil samples will first be homogenizedby vigorous shaking; 20 g of soil will then be mixed with 200 mL of sterile water and homogenized by mechanical agitation. Serial dilutions will be prepared as needed for pond and soil samples. Duplicates and sterile blanks will be run for every 10th sample.

Measurement of Selected Pathogens and Microbial Source Tracking: Aliquots of stored water, mother and child hand rinse samples, and soil samples from the child's play area will be collected and pre-processed to concentrate organisms for molecular detection of enteric pathogens, and human and animal specific fecal markers during the two specified rounds of data collection. Aliquots from hand rinse samples (50mL),

stored water samples (100 mL) and homogenized soil samples (5g) will be designated for molecular work. Each water and hand rinse sample aliquot will be vacuum filtered through a 0.45 uM-pore sized filter (HA filter) in order to capture bacterial and viral DNA. Prior to filtration, 0.5 mL of 2.5 M MgCl2 will be added to every 50 mL of sample filtered to facilitate the capture of virus particles on the filter. Filters will then be treated with 500 uL of RNA/DNA stabilizing agent (RNAlater, Qiagen), vacuum aspirated, then stored at -80°C until transport to UC Berkeley. An aliquot of 2g of homogenized soil samples will be measured out and placed in DNA free centrifuge tubes with 1mL of RNAlater, vortexed for 20 seconds, then placed at -80°C until transport to UC Berkeley. A second aliquot of 3g will be measured to determine the soil moisture content.

Archived filters and soil samples will be shipped to UC Berkeley at room temperature, then stored at -80°C until DNA extraction and molecular analysis. RNA and DNA will be extracted simultaneously from water and hand rinse filters using the MoBioPowerWater RNA isolation kit, and RNA and DNA will be extracted from soil samples using the MoBio RNA PowerSoil Total RNA isolation kit. Selected pathogens and fecal source markers will be detected using the PCR, multiplex PCR, and qPCR assays.

Measurement of Soil-Transmitted Helminths: Soil samples will be cleaned and helminth eggs will be concentrated through settling, sieving, and floatation steps. Processed samples will be enumerated by direct microscopy in duplicate by two different lab technicians trained in parasitology. The number of Ascaris lumbricoides, Trichuris trichuria, Ancylostoma duodenale and Necator americanus eggs will be counted. Multiple microscopic slides will be prepared and read for each sample, if necessary. Egg counts will be multiplied by the total grams analyzed to determine the concentration of eggs per gram of soil. Samples will be processed and read within 6 hours of collection.

Monitoring Of Uptake:
At each quarterly visit, we will monitor the presence of functional latrine and feces disposal hardware, latrine use and feces disposal practices and presence and quantity of feces in the living environment using spot check observations. We propose collecting repeated measures because spot check observations are a noisy indicator of household behaviors because of temporal variation, and a longitudinal index based on repeated measurements can more finely distinguish meaningful behavioral patterns [Gorter 1998, Ruel 2002]. We also expect time trends in the uptake of the latrine intervention; uptake might initially be low due to unfamiliarity, increase with behavior change promotion and taper off as the novelty dissipates. Quarterly data will allow us to monitor these trends.

We will augment the spot checks with a novel, Passive Latrine Use Monitor (PLUM) that has been developed and validated by members of our team specifically for monitoring latrine usage. We will deploy 30 PLUM sensors in our study population of 720 households in rotating fashion (15 sensors per arm rotated in 24 waves per year in two-week periods) for the two-year study period.The PLUM sensors have been shown to be widely acceptable to households in rural India [Clasen 2012]. PLUM uses a passive infrared sensor and a door switch to discretely and anonymously measure latrine visits. The sensors will enable us to measure the number of latrine visits per household as well as a rich set of information about defecation practices (event frequency, timing, duration) impossible to collect without structured observation. The advantage of sensors over traditional structured observation is that they are less expensive and are less likely to cause measurement bias by reactivity, a demonstrated problem with structured observations of sanitation and hygiene practices [Ram 2010].

Monitoring of Child Health Outcomes:

At each visit, we will also administer a brief interview to the primary caregiver of the index child to assess caregiver reported diarrhea for the index child and other children < 60 months living in the compound.

6. Midline Assessment
Field teams will measure outcomes at 1 year following the initiation of intervention. Children will be between 8 and 15 months at the 1-year survey. This will be the first round in which the field team measures anthropometry. The team will collect information about how many weeks the children and their mothers stayed in another village to understand how many weeks they were out of interventions. The field teams will measure length, weight, and head circumference using standardized measurement techniques. Our anthropometric teams will have been trained and standardized in measurement techniques according to the FANTA and WHO guidelines (Cogill 2003, deOnis 2004). The child will be weighed using a calibrated scale and measure his/her length or height using a height board and head circumference using a tape measure. We will also measure maternal height and weight, and will conduct an assessment of short term maternal stress. The common modules that will be utilized at the midline assessment include:

Module 0. Tracking information
Module 1. Birthdate, age, and sex measurement
Module 2. Diarrhea and illness symptoms
Module 3. Deworming
Module 4. Anthropometry
Module 5. Vaccination history
Module 6. Child Food frequency questionnaire (24 hour and 7 day recall)
Module 7. Handwashing assessment
Module 8. Sanitation assessment
Module 9. Child defecation and feces disposal assessment
Module 10. Water treatment, storage, and quality assessment
Module 12. Home care environment
Module 13. Measures of spillover
Module 14. LNS measurement
Module 15. Environmental microbial assessment and Quantitative fly assessment (subset of households)
Module 16. Children's motor milestones using WHO validated tool and language development via Bangladesh adapted MacArthur Communicative Development Inventories at midline
Module 19. Maternal depression
Module 20. Environmental enteropathy assessment (subset of study population)

We will measure diarrhea morbidity using caregiver report with a 48-hour recall period. We will collect information on index children's deworming medications. The assessment of the sanitation interventions will be the spot checks of latrine structures to assess type, cleanliness, stated use, and state of repair as well as the presence of child feces or other feces that appears to be human in or near the compound. Measures of fly density and sentinal object (Child Toy) contamination will also be assessed.

Assessments of secondary child outcomes

Child:

In assessing the effects of the WASH and nutrition interventions on child health and wellbeing, we will measure some aspects of children's development that may be affected by the treatments. The links between nutrition and cognitive development are clear (Grantham-McGregor et al 2007, Walker et al 2007, Allen et al 2001, Sigman, 1995), but the pathways through which diarrhea and WASH interventions may affect child development are still speculative (Humphrey 2009, Walker et al 2011, Bowen et al 2012). A recent study demonstrated that intensive handwashing interventions for 7 months during the first 30 months of life predicted higher development scores across a range of domains (adaptive, personal-social, communication, cognitive, and motor) at 5-7 years of age (Bowen et al 2012). However, the precise mechanisms of how the treatments improved child development could not be determined. The present study provides the unique opportunity to rigorously examine the associations between WASH and nutrition interventions, child nutritional status, tropical enteropathy and child development outcomes. The findings have the potential of making novel contributions to the WASH, nutrition and child development fields.

We will measure children's motor and language development in all children ~8-15 months of age (that is, born during or after the baseline). Motor skills (sitting, walking, standing) will be assessed directly, using a WHO validated protocol (Wijnhoven et al 2004) adapted for use in Bangladesh. The motor milestone scale has been widely used throughout the world to detect nutritional effects on motor acquisition in Africa (Adu-Afarwuah 2007, Kariger et al 2005), Nepal (Siegel et al 2005) and Bangladesh (Hamadani et al 2013; Tofail et al 2006). Language skills (understanding and speaking words) will be assessed via parent report using the MacArthur Communicative Development Inventory (CDI) (Fenson et al 1994). The CDI is a well-established measure that has been used in more than 40 dialects to describe language development in infants and young children as well as identify group differences in language development (Law &Roy, 2008). The measure provides a valid and reliable method for assessing language in large groups of very young children. Both measures were validated for use in Bangladesh, and have successfully discriminated development in populations with poorly nourished children (Hamadani et al 2010; Tofail 2006) .

Assessments for potential interactions

Child
We will collect a sample of blood for future genetic testing and testing of nutrition and other biochemical parameters, a urine sample for chemistry and a stool sample to permit future testing of the intestinal microbiome from each enrolled child. For the blood sample we will separately freeze aliquots of sera for eventual biochemical analysis, and retain a clot for genetic testing.

We will measure indicators that describe the intake of food for the infants by interviews conducted at the household level using a household survey methodology. The indicators and the instruments of the household survey will be adapted from WHO and UNICEF guidelines on "Indicators for assessing infant and young child feeding practices: Part 2 Measurement" (WHO, 2010). We will measure 24 hours recall and 7 days recall for the indicators of food frequency.

Mother
We will measure the mother's height and weight and administer the parental stress index (Module 21).

We will collect data on the mother's height and weight, and maternal depressive symptoms (Module 19) and maternal cognitive abilities to control for their influences on child growth and development. There is substantial evidence that maternal characteristics -- such as education, intelligence and depression -- are

associated with infant undernutrition and poor developmental outcomes (Anoop et al 2004; Wachs et al 2009; Walker et al, 2007; 2011). The Centers for Epidemiological Studies-Depression Scale (CESD) (Radloff, 1977) is a brief, widely used measure of 20 statements that assess the likelihood of depressive symptomology. The ICCDR,B psychologists have adapted and used the CESD in various studies, and have noted relationships between higher scores (indicating depression risk), and stunting and lower developmental scores in young children (Black et al 2009; Nahar et al 2012). We will administer the adapted Bangladesh version of the CESD to all mothers of children 8-15 months of age.

Information on maternal education was collected at baseline, but to ensure we are adequately capturing the possible effects of maternal intelligence on child outcomes, we will measure cognitive functioning using three different measures: the Mini-Mental Status Exam, the Digit Span Task and the Verbal Fluency task.

The Mini-Mental Status Exam is a measure of cognitive functioning used in many parts of the world (Mitchell 2009) that has been adapted in Bangladesh for use with illiterate populations (known as BAMSE) (Kabir&Herlitz 2000) . It assesses orientation (knowledge of day, current prime minister), memory, simple calculation, capacity to carry out instructions, and summarization of a short (oral) story. The BAMSE has been used successfully in Bangladesh to detect the effects of early child health interventions on adolescent development (Barham&Calimeris 2008) and the association between malnutrition and cognitive development in older adults (Ferdous et al 2010). The BAMSE will be administered to all mothers of children 8-15 months of age.

The other cognitive measures that we will use with mothers of children 8-15 months of age includes Digit Span and Verbal Fluency Tests. Both the tests are classic tests ofworking memory and short-term memory that has been used around the world. We will specifically use Digit Span (Backward) test that requires respondents to repeat back a string of digits (3-7 in length) in reverse order and has also been used in Bangladesh on primary school aged children (Baddely, 1992; Wechsler, 1994, 1997; Wasserman et al 2011, Huda et al 2001). We will also administer a Verbal Fluency task, which assesses cognitive processing speed by asking respondents to name as many animals as possible in 60 seconds. The test has been used in Jamaica (Baddely et al 1995), Tanzania (Jukes et al 2002) and nutritional trials in Bangladesh (Huda et al 1999; 2001). Both the tests were piloted at field level on 80 rural mothers for the current study with good test-retest reliability, r value for Digit Span and Verbal Fluency were 0.72 and 0.91 respectively and good correlation with socio-demographic variables

Home Care Environment
It is well established that the home care environment has a large influence on child health and development (Bradley &Corwyn, 2005; Grantham-McGregor et al 2007; Walker et al 2007 and 2011). We will control for the influence of the home environment on child and health outcomes by collecting data using items adapted from the Home Measurement for Observation for the Environment (HOME) (Bradley et al 2001; Ertem et al 1997) and from the UNICEF Multi-Indicator Cluster Surveys (Kariger et al 2012). Items from these measures have been used to determine differences in child development in Bangladesh (Hamadani et al 2010)

Environmental microbial assessment
Measures of fly density, sentinel object (Child Toy) contamination, hand contamination and drinking water contamination will also be assessed within the subset of households that has been selected for environmental enteropathy measurements (80 clusters (500 households) from each of four arms-- the

---

Protocol Title: Measuring the benefits of sanitation, water quality, handwashing and nutrition interventions for improving health and development in rural Bangladesh

Protocol Status: APPROVED

Date Submitted: 08/18/2014

Approval Period: 09/16/2014-11/03/2016

Important Note: **This Print View may not reflect all comments and contingencies for approval. Please check the comments section of the online protocol. Questions that appear to not have been answered may not have been required for this submission. Please see the system application for more details.**

control group, the combined WASH interventions, the nutrition intervention and the combined WASH and nutrition interventions, for a total of 2000 households). In addition, we will measure hand contamination and drinking water contamination among a subset of up to 360 households per arm in the single intervention (water and hygiene) arms. The sample collection and analysis procedures are detailed below.

Drinking Water
In the 2000 households selected for environmental enteropathy measurements as well as in a subset of households in the water arm, we will ask the caregiver of the target child to give us a glass of water as if giving it to her child. We will collect 250 ml of this water in a sterile Whirlpak and record whether it came from a tubewell or a storage container. We will also ask the caregiver if the water has been treated in any way. All samples will be delivered on ice to the ICDDR,B Laboratory within 24 hours of retrieval for analysis. We will use standard membrane filtration methods to quantify the number of colony forming units (cfu) of E. coli. Sample aliquots of 100 ml will be filtered through 0.45 μm Millipore member filters and filtered samples will be plated on MI Agar.

Target Child Hand Rinse
In the 2000 households selected for environmental enteropathy measurements as well as in a subset of households in the hygiene arm, we will collect a hand rinse sample by rinsing the hands of the target child, one at a time, in 200 mL of sterile water in a sterile Whirlpak bag. All samples will be delivered on ice to the ICDDR,B Laboratory within 24 hours of retrieval for analysis. We will use standard membrane filtration methods to quantify the number of colony forming units (cfu) of E. coli. Sample aliquots of 10 ml will be filtered through 0.45 μm Millipore member filters and filtered samples will be plated on MI Agar and incubated at 35°C.

Sentinel Toy
In the 2000 households selected for environmental enteropathy measurements, we will assess environmental contamination using a sentinel non-porous toy ball. The toy ball will be initially sterilized and stored in a sterile bag or aluminum foil until it is given to the selected households. After one day, a field assistant will return and ask the mother to locate the toy ball without touching it to avoid hand contamination. The field research assistant will use sterile gloves to retrieve the toy and place it in a sterile Whirlpak bag containing 200-250ml of recovery media (water with salts) or sterile water. The toy will be immersed and bathed in the recovery media for 15 seconds. The field research assistant will remove the toy, place the sealed bag on ice packs, and then wash the toy with soap and water before returning it to the household. All samples will be delivered on ice to the ICDDR,B Laboratory within 24 hours of retrieval for analysis. We will use standard membrane filtration methods to quantify the number of colony forming units (cfu) of thermotolerant fecal coliforms. Sample aliquots of 10 ml and 100 ml will be filtered through 0.45 μm Millipore member filters and filtered samples will be plated on MI Agar and incubated at 44.5°C modified fecal coliform (mFC). If necessary, 10-fold dilutions will be made and plated following the same protocol.

Fly Density
In the 1500 households selected for environmental enteropathy measurements, we will count and speciate flies caught in the latrine and food preparation areas of the compounds using the methods detailed under baseline assessment.

The assessment for the handwashing intervention will be the presence of soap/soapy water and water at

the handwashing station, the per capita consumption of soap in the compound, and the assessment of visible dirt on mothers and children's hands. Assessment of hand contamination will be through the use of target child hand rinse samples to test for the presence of E. coli in the environmental enteropathy subset and in a subset of households in the single intervention arms, as discussed above.

The primary assessment of drinking water quality will be through the use of household drinking samples to test for the presence of E. coli in the environmental enteropathy subset and in a subset of households in the single intervention arms, as discussed above. We will also assess the presence of reportedly treated water through tests for chlorine residual, obvious evidence for unused Aquatabs, and the presence and accessibility of the provided storage container (e.g. not readily accessible where water is consumed).

The primary assessment of compliance with the LNS intervention will be through monitoring the remaining LNS sachets unused at the end of every month. In addition, caregiver knowledge, attitudes and practices related to LNS usage and complementary feeding practices will be assessed.

The assessments of each of the interventions will also include questions that address the major behavior change constructs for social cognitive theory, for new habit formation and for community mobilization.

Environmental Enteropathy biomarkers will also be measured, as described above (2. Baseline Assessment). We collected 5 ml of blood from each EE participant at midline.

7) Endline Assessment (21 – 27 month)
The final assessment will take place 2 years after the initiation of intervention. Children will be between 21 and 27 months in age. The common modules to be included are:

Module 0. Tracking information
Module 1. Birthdate, age, and sex measurement
Module 2. Diarrhea and illness symptoms
Module 3. Deworming
Module 4. Anthropometry
Module 5. Vaccination history
Module 6. Childfood frequency questionnaire (24 hour and 7 day recall)
Module 7. Handwashing assessment
Module 8. Sanitation assessment
Module 9. Child defecation and feces disposal assessment
Module 10. Water treatment, storage, and quality assessment
Module 12. Home care environment
Module 13. Measures for spillover
Module 14. LNS measurement
Module 16. Child development
Module 19. Maternal depression
Module 20. Environmental enteropathy subsample
Module 21. Maternal intelligence

Trained field staff will repeat the anthropometric, child development, and diarrhea morbidity assessments. Spot checks and behavior change assessments will be performed as described above (see Midline Assessment).

Child developmental measures for endline will include the A-NOT-B and Tower Tests for measuring working memory, inhibition and executive function of the children. The A-NOT-B tasks require children to search for objects hidden in specified locations after short delays and reversals. The procedures are based on protocols described in Epsy, K. A., Kaufmann, P. M., McDiarmid, M. D., & Glisky, M. L. (Espy et al 1999). The Tower Test consists of building a tower with the child, and assesses how well children can inhibit responses and impulses and follow directions. The A-NOT-B and Tower Tests are direct tests of the child. In addition, we will use two parent report measures to gather information on development in various domains. These are (1) the MacArthur Communicative Development Inventory (CDI) (Fenson et al 1994; 2007), also administered at midline, that documents words children speak and understand; and (2) the extended and adapted version of the Ages and Stages Questionnaires (Bricker et al 1999; Fernald et al 2012), which will gather information on children's communication, gross motor and personal social skills. (Boyce et al; Fernald et al 2012; Fenson et al 2007).

Information on maternal education was collected at baseline, but to ensure we are adequately capturing the possible effects of maternal intelligence on child outcomes, we will measure cognitive functioning using the Backward Digit Span Task (Baddelly, 1992; Wechsler, 1994, 1997). This short-term memory and information manipulation test requires respondents to repeat back a string of digits (3-7 in length) in reverse order. The test has also been used in Bangladesh on primary school aged children (Wasserman et al 2011, Huda et al 2001). The test was piloted at field level on 80 rural mothers for the current study with good test-retest reliability, r value 0.72, and scores showed good correlation with socio-demographic variables.

The assessment of the sanitation interventions will be the spot checks of latrine structures to assess type, cleanliness, stated use, and state of repair as well as the presence of child feces or other feces that appears to be human in or near the compound. We will also collect information about pit switching and pit emptying for the households who have received dual pit latrines. Measures of fly density, sentinel object (Child Toy) contamination, hand contamination and drinking water contamination will also be assessed.

We will collect a sample of blood and urine from each child for future testing of nutrition and other biochemical parameters and a stool sample to permit future testing of the intestinal microbiome from each enrolled child. Furthermore, after endline is completed, we will collect 10 additional monthly follow up stool samples from 60 children enrolled in the EE subset (20 children in the WASH arm, 20 children in the nutrition arm, and 20 children in the control arm). These additional stool samples will be used for future testing of the intestinal microbiome.

Environmental Enteropathy, allostatic load, and telomere length biomarkers will also be measured in a subset of participants, as described above (Midline Assessment). In addition, at endline, whole blood hemoglobin will be measured at the time of blood sample collection using a portable spectrophotometer (Hemocue 301). An aliquot of whole blood will be also be sent to the Thalassemia Center at Shishu Hospital to test for markers of inherited hemoglobin disorders (thalassemia and HbE). We will collect a total of 7.5 ml of blood from each EE participant at endline. The increase in blood volume at endline reflects the addition of the anemia measurement.

Intestinal parasitic infections will also be measured at endline. We will collect stool and blood spot samples from 7 target children per cluster and the older child living in the same household compound. The procedures will be the same as described in the Baseline Assessment section. Additionally, at endline, an aliquot of each stool sample will also be processed by Kato Katz microscopy for detection of Ascaris, hookworm, and Trichuris ova (Albonico et al., 2012)- the most common soil transmitted helminth infections in Bangladesh affecting children. All children will be provided with deworming medicine at endline. Field workers will offer it to children because it is logistically too difficult to provide results of tests for worm infections to participants, so they provide it to them all regardless of results in case they are infected. This is a standard practice in research on soil-transmitted helminths. Furthermore, deworming is commonly distributed in mass drug administrations around the world (including Bangladesh) to all school age children, regardless of infection status.

Chlorine water testing will be conducted for households that report treating their water. Water samples will be collected from all households and after departure from the home, field staff will test on the samples from those that indicate using Aquatabs to treat their water. In addition, we will collect a sample from source of drinking water from all the households during endline to measure the arsenic concentration using EconoQuick kits.

Sustainability: We propose to add some questions in endline survey in different sections to assess whether participants will maintain their behaviour once we stop visiting their households and stop providing them supplies. The purpose of adding sustainability questions is to obtain a baseline for comparison to use after we survey again in 1-2 years. We have developed these questions through a process of discussion and revision with several of the WASH Benefits team members. Currently, we are in the process of finalizing the list of sustainability questions through a broader discussion. There are three domains of sustainability questions. The first domain includes questions regarding whether participants have the knowledge and skills to use and maintain latrines, safely store water, wash their hands, and provide nutrient-rich complementary foods to their children. The second domain includes questions regarding participants' self-efficacy to use and maintain latrines, safely store water, wash their hands, and provide nutrient-rich complementary food to their children. The third domain includes questions regarding some of the physical items and structures present at the time of the survey such as the types of water storage containers in the home and the condition of the latrines. We will also take a photograph of the latrine slab to use as a baseline.

Monitoring and Process Documentation

Valid evaluation of the study hypotheses requires a consistent intervention, but the WASH Benefits intervention is a large complex intervention spread over 5 districts. While an intervention of this scope and complexity involving this many individuals will inevitably have some deviation from optimal implementation as planned, meaningful interpretation of the results requires a rigorous assessment of how consistently the intervention activities were delivered. This assessment will include 3 components:
1)A monitoring system that tracks
a)procurement and distribution of commodities and technologies
b)recruitment, training and supervision of promoters

2)An unannounced fidelity assessment of the delivered intervention (Appendix 6)
a)The fidelity assessments will be conducted
i)In each of the initial 1216 intervention implementation clusters after 1, 2, 3 and 4 months of intervention
ii)In each of the second group of 1216 implementation clusters after 4 months of intervention
iii)In each of the third group of 1216 implementation clusters after 3 months of intervention
iv)In each of the fourth group of 1216 implementation clusters after 2 months of intervention
v)In each of the fifth group of 1216 implementation clusters after 1 month of intervention
vi)In each of these groups (i-v) subsets of BCC samples 50% which is 4 out of 8 households
vii)Assessments will continue in 24intervention clusters (4 blocks) per month (4from each of the 6 interventions with a subsets sample 50%)

Revised fidelity assessment strategy :
The revision of the fidelity assessment will be made to see the uptake status both for initial phases as well as new phases with a intention to have a similar number of samples from new phases and reducing samples from initial phases.

Stages:
Selection of 1 block from more than 6 months intervention implementation blocks from each phase (10 blocks phase = 1 stratum) and selection of 2 blocks from each phase (stratum) where intervention implementation less than or equal to 6 months.

Selection of 9 blocks (1 block from each phase) randomly after 6 assessment months and it will continue up to reaching 24 months by phase one.

Fidelity assessment will be ended in one point of time when the first phase will complete 24 months of intervention

b)The focus will be on the households that received the intervention and will address key elements of the intervention
i)Are each of the elements of hardware distributed to the appropriate home
ii)Are each of the elements of distributed hardware functional?
iii)Is there objective evidence of uptake
(1)Handwashing
(a) presence of a HW station
(b)Water present?
(c)Soap present?
(d)Soapy water bottle present?
(e)Soapy water present?
(f)Is all equipment in working order?
(2)Water
(a)Is the icddr,b provided water storage container present?
(b)Are Aquatabs present?
(c)Is drinking water in the storage container?
(d)Is the equipment in working order?
(e)Are the containers being used for storage of other liquids?
(f)Is there detectable residual chlorine

(3)Sanitation
(a)Is the potty available?
(b)Is the potty immediately available to the child?
(c)Is the potty in working order?
(d)Is the poop scoop available within 30 seconds?
(e)Is the poop scoop in working order?
(f)Does the latrine show signs of use?
(g)Is there an odor of feces in the latrine?
(h)Is the latrine in working order?
(4)Supply of LNS
(a)How many days ago was it delivered?
(b)How many sachets are present?
(c)How many sachets have been used?
iv)For each intervention, 1 – 3 key questions will be asked of persons who received the intervention to see if they received the principal message of the intervention
v)How many times has the community health worker visited
(1)in the preceding week?
(2)in the preceding month?
c)The data will be collected by smart phone, that is uploaded to ICDDRB server the day it is collected with reports produced every month and circulated to the management team.
d)Assessments are front loaded so that early problems can be addressed through refresher training, additional supervisory visits or other appropriate strategies.
3)A process documentation system that tracks operational problems and how they were addressed, and modification made to intervention and the reasons for these modifications.

A primary goal of the process documentation is to provide the CHP supervisors with the information necessary to ensure the intervention is implemented according to the prescribed strategy and to identify areas where additional communication and training would be helpful. In addition an external consultant has been contracted to conduct process evaluation throughout the intervention.

Structured Observation

Trained field workers will conduct structured observation in a subset of participating households to observe how the interventions are being used. We will randomly select 6 blocks from each of the nine Phases of study area. From each of the selected 54 blocks, we'll randomly select one household in each cluster. Therefore a total 432 households will be selected for structured observation during midline. If the uptake seems to be low during midline below the benchmark, we will consider conducting structured onservation during endline.

If the structured observation data suggest important difficulties in uptake and use of the interventions, then the project leadership will consider additional qualitative investigation (detailed below) or changes to the intervention.

Additional structured observations will be conducted in a total of 150 households (subset of the 2160 households enrolled in the in-depth environmental contamination assessment) in the sanitation alone arm

(n=50), the combined sanitation, water, and hygiene arm (n=50), and the control arm (n=50) to characterize hygiene, water treatment, food hygiene, and sanitation behaviors in richer detail and with an emphasis on child exposure to feces or fecal contamination (consent form in Appendix 1r). Each structured observation will allow for observation of child defecation events and child feces disposal practices, child feeding events, latrine usage, food preparation, food storage, and handwashing behavior at critical times.

To complement the structured observations, we will obtain video surveillance of practices and activities in the same 150 households to capture key water, sanitation and hygiene practices and behaviors. Video surveillance will be conducted by local women trained to conduct video data collection; videographers will also record notes on behaviors during the observation. Behaviors includes personal hygiene, household water management (water usage and storage pattern), handwashing practices at critical times, object contact with children's hand and mouth, presence of feces within and surrounding the households, defecation practices of older children (3+ years) interactions with domestic and pet animals, dealing with and disposal of animal feces, contact with mother and family members. Observations will be made of how commonly children come in contact with soil in their own and neighboring compounds, play with children including those from neighboring compounds.

Qualitative Investigation

We will conduct up to 45 in-depth interviews (subset of the 2160 households enrolled in the in-depth environmental contamination assessment) in the sanitation alone arm (n=15), the combined sanitation, water, and hygiene arm (n=15), and the control arm (n=15) to explore individual beliefs and perception of certain practices that contribute to fecal contamination (consent from in Appendix 1s). We will focus on personal and household hygiene behaviors (handwashing, food preparation, water management), decision making processes, empowerment, defecation practices among children, women, sick and older people; animal rearing methods (open or corralled, within or outside the households) contributing to environmental fecal contamination. The in-depth interview guidelines are in Appendix 3 (Module 53).

We will conduct at least:
•30 interviews with primary and secondary caregivers of the children,
•5-10 interviews with the person accompany a child most of the time in absence of a caregiver (grandmothers, older siblings, aunts of the child).
•5-10 interviews with fathers of the children to explore their concern and role on child's health and development.

The principal and co-principal investigators will carefully review the intervention fidelity assessments and identify any areas of low uptake of interventions. Critical benchmarks for uptake based on unannounced visits are:

The principal and co-principal investigators will carefully review the intervention fidelity assessments and identify any areas of low uptake of interventions. Critical benchmarks for uptake based on unannounced visits are:

•Handwashing promotion households

Protocol Title: Measuring the benefits of sanitation, water quality, handwashing and nutrition interventions for improving health and development in rural Bangladesh

Protocol Status: APPROVED

Date Submitted: 08/18/2014

Approval Period: 09/16/2014-11/03/2016

Important Note: **This Print View may not reflect all comments and contingencies for approval. Please check the comments section of the online protocol.**
Questions that appear to not have been answered may not have been required for this submission. Please see the system application for more details.

o65% of households have at least one handwashing station with soap and water present
•Water quality intervention households
o65% of households with children 6 – 24 months of age have stored chlorinated drinking water
•Sanitation
o80% of households have a potty easily accessible to mother
o50% of households have a potty easily accessible to child 12 – 36 months of age
o80% of households have a sani-scoop easily accessible to mother
o80% of households have a latrine with a functional water seal
•Nutrition
o80% report hearing any messages on infant/child nutrition and or Sonamoni
o90% report at least one visit by the CHP in household to discuss infant and child nutrition
oWithin households with targeted children > 6 months of age, the stock of LNS sachets in 70% of households is consistent with daily use of two sachets per day

If any of the uptake measures are below the critical benchmarks, then a qualitative team will review the monitoring and process documentation in the low performing area, visit the site of the low uptake, meet with community hygiene promoters, supervisors and study subjects and troubleshoot the cause of the low uptake. Because these interventions have each been piloted and in the pilots achieved these benchmarks of uptake, we expect that uptake below the benchmark will indicate a problem where the intervention was not implemented as planned, and the investigation will identify needs to provide additional training or other support to achieve the planned intervention.

While unlikely, it is also possible that the community hygiene promoters will be implementing the intervention precisely as planned, but uptake is lower than expected. If uptake is below the benchmark in the setting where implementation followed the prescribed approach, the qualitative team will conduct more in-depth evaluation will be framed around the Integrated Behavioral Model for Water, Sanitation and Hygiene (IBM-WASH), based on the earlier Integrated Model for Hygiene, Point-of-use water treatment and Sanitation behaviors (IMHPS). The IBM-WASH was developed and refined based on the pilot phase of the WASH Benefits project, as well as the concurrent Cholera Behavior Change (CBC) study carried out in Mohammadpur, Dhaka. It also incorporates behavioural determinants from a number of previous models used for WASH behaviour change interventions. This ecological (multi-level) model has 5 levels (Societal/structural, Community, Interpersonal/household, Individual and Behavioral/Habitual) and 3 dimensions (Contextual, Psychosocial and Product/Technology).

One investigation in one cluster will involve up to:
•5 interviews with implementation partners
•10 observations of household visits by CHPs
•10 household-level interviews with 3 individuals in each household: One person responsible for maintaining the product/hardware e.g. handwashing station, and two users of the product/hardware.
•10 household-level observations of the product, its condition and associated factors affecting its use.

We anticipate 10 investigations per year, for a total of up to 35x10 = 350 interviews and 10x10 = 100 observations of CHP visits and 10x10 =100 household-level observations.

The objective of these investigations is to rapidly identify problems that can be addressed throughout the intervention to ensure a consistent high quality intervention with regular uptake by study participants.

Protocol Title: Measuring the benefits of sanitation, water quality, handwashing and nutrition interventions for improving health and development in rural Bangladesh

Protocol Status: APPROVED

Date Submitted: 08/18/2014

Approval Period: 09/16/2014-11/03/2016

Important Note: **This Print View may not reflect all comments and contingencies for approval. Please check the comments section of the online protocol. Questions that appear to not have been answered may not have been required for this submission. Please see the system application for more details.**

Ethnographic investigation
Two female researchers will stay for approximately two months in two communities at a time and collect data through observations during waking hours in a natural setting when the activities related to defecation, handling fecal matter or waste disposal occurs. The selected communities will not be enrolled in the WASH Benefits study, but will be near the WASH Benefits study area. The ethnographers will collect data through participant observation (unstructured), informal conversation, in-depth interviews (consent form in Appendix 1s) and focus group discussions (consent form in Appendix 1t) when necessary with community members, for a total of 15 in-depth interviews with adult males and females, with the primary and secondary caregivers of children to record beliefs and practices related to water, sanitation and hygiene. The researchers will also conduct 6 (depending on the community setting) focus groups with the community members. The focus group guidelines are in Appendix 3 (Module 54).

Lead Assessment
At baseline mothers have a blood sample collected using lead-free blood collection equipment, and are asked where most of the rice they eat comes from. We will randomly select 500 mothers for whom most of the rice they eat comes from their own fields and will analyze their blood lead levels in the nutritional biochemistry laboratory at icddr,b. Lead will be measured in whole blood by the Graphite Furnace Atomic Absorption Spectrometry (Shimadzu). For quality control, Standard Reference Material from the National Institute of Standards and Technology (SRM, NIST) is used for external quality control purpose. For internal quality of the assay, duplicates are run as well as recovery checks in every lot are performed to maintain the quality.
Qualitative researchers will visit 15 of the women with high blood lead levels and seek informed consent for participation in this component of the study (Appendix 1n). They will observe the women's living environment and ask questions about various potential sources of lead exposure including pesticides, herbicides, fertilizer, industrial processes or wastes that use lead, cosmetics and canned food (Appendix 3, Module 50). Through these conversations they will generate a list of potential exposures. The anthropologists will visit four area shops that sell agrochemicals, seek the informed consent of shop proprietors (Appendix 1o) and ask questions to understand the range of chemicals available and their typical selling pattern (Appendix 3, Module 51). They will use this information in discussion with the women and whomever in the household purchases and applies agrochemicals to develop a taxonomy that explicates how people in this area classify the various chemicals and so how questions can be framed to assess exposures. The anthropologists will also ask questions about the process of applying agrochemicals, including who mixes and applies them, how they are mixed and applied, where the chemicals are stored, and how the containers that stored the chemicals are used or disposed (Appendix 3, Module 51).
The project collaborators will review the findings from the in-depth interviews and use these to revise the multiple choice questions to explore potential exposures to lead in these communities (Appendix 3, Module 52). We will classify the 100 mothers with the highest blood lead levels as cases and the 100 mothers with the lowest blood lead levels as controls. Field research assistants will re-visit these mothers and seek their consent to participate in this component of the study (Appendix 1p). The field team will administer the questionnaire to the cases and controls.
During the household visit the field team will collect a sample of uncooked rice that was grown in the study participant's field and a sample of soil from the field. To collect the soil sample, the field worker will ask the respondent to identify the agricultural field where most of the rice that they consume is grown. They will identify the corner of the field that is the farthest from the household and designate the border of the field

---

Protocol Title: Measuring the benefits of sanitation, water quality, handwashing and nutrition interventions for improving health and development in rural Bangladesh

Protocol Status: APPROVED

Date Submitted: 08/18/2014

Approval Period: 09/16/2014-11/03/2016

Important Note: **This Print View may not reflect all comments and contingencies for approval. Please check the comments section of the online protocol. Questions that appear to not have been answered may not have been required for this submission. Please see the system application for more details.**

that is most closely aligned east to west access as the x-axis and the border that intersects it as the y-axis. They will consult a random number table that lists random integers between 1 and 9. They will use these 2 numbers as coordinates measured in meters to identify a point within the field to collect a soil sample. If the random number selected corresponds to a point that is not located within the field, the field team will select the next random number in the table. The field team will identify 3 coordinates within the field.
At each of the selected coordinates, field workers will collect soil to a depth of 25 cm (rooting depth) using a 2 cm diameter push corer. Field workers will extrude soil from the corer and placed in plastic, zip-lock bags. Soil and food samples will be shipped to Stanford, dried and ground. The samples will then be examined by X-ray fluorescence (XRF) spectroscopy to determine the concentrations of Pb. The XRF analysis will be conducted in the Environmental Measurements facility (http://em-1.stanford.edu/) at Stanford, which is shared analytical facility in the School of Earth Sciences.
We will analyze the questionnaire data, soil and food lead levels and assess which exposures are statistically associated with elevated blood lead levels.
The anthropology team will follow up on the exposures that are identified in the case-control study to determine how and why people come in contact with this source. If the analysis supports the hypothesis of agrochemical contamination as the primary pathway of lead exposure in this population, the field team will return to a subset of houses over the course of the season to collect a representative sample of agrochemicals to understand more thoroughly which chemicals are being applied and identify those that are most likely to contain lead for further testing.
Once the source of lead is confirmed, by confirmation of high levels of lead in the pathway statistically associated with high lead levels in the mothers, the Bangladesh anthropology team will return to the community to explore the patterns of lead containing product use and trade further. Where are the products purchased? Why are these specific products used, and where do these products come from? Who sells the products, and why do they sell these product and not others? We will trace back the source of production as far as possible and explore any existing regulations and the process of their enforcement.

Spillover substudy
The household with a child 0-59 months that is nearest to a compound enrolled in the combined intervention and control arms will be eligible for this study. We will administer a survey to measure caregiver reported diarrhea and respiratory illness of children under five years. We will define diarrhea as 3 or more loose or watery stools in 24 hours, or ≥1 stool with blood. Respiratory illness will be defined as a persistent cough or difficulty breathing in the 7 days before the interview. Symptoms will be reported by caregivers collected daily for the 7 days preceding the interview. We will collect a stool sample and analyze it for soil transmitted helminth (Ascaris, Trichuris, hookworm) ova in stool specimens among children 0-59 months. We focus on this age range because children in this range experience the greatest burden of diarrhea and respiratory diseases and are not covered by the national school-based deworming campaign. To measure helminth infection, field workers will deliver a stool collection kit, give instructions to the caregiver, and return the following day to collect the specimen. Field staff will collect specimens, preserve them, and detect helminth ova using the Kato-Katz technique.

Field workers will also visually inspect of mother and children's fingers, fingernails, and palms for dirt and assess the presence of a handwashing station and soapy water bottles in the kitchen/latrine. They will take a sample of drinking water typically provided to a child, and we will analyze the samples for E. Coli using DelAgua kits. Field workers will also count feces piles in courtyards. They will assess contamination of sentinel objects using the methods outlined above. They will assess fly density in the compound by recording the number of flies that land on sticky cards affixed to walls near where food is prepared for a 24 hour period.

b) Explain who will conduct the procedures, where and when they will take place. Indicate frequency and duration of visits/sessions, as well as total time commitment for the study.

Trained field staff will conduct all anthropometry, diarrhea morbidity, and child development assessments in the participant's home or at a nearby field office at the beginning of the study, one year following intervention implementation, and 2 years following intervention implementation. These assessments will take about 1 hour to complete, and we expect the baseline/enrollment visit to require 2 hours total.

Local health promoters will visit households several times weekly early in the study to encourage families to adopt the new behaviors, help with solving problems and answering questions. Visits will gradually taper off to at most monthly by the end of the two year period, though participants will be encouraged to contact the promoters if they encounter equipment breakage, or consumable product shortage. In the arms receiving the nutrition intervention, the promoters will deliver the supplement to target households, teach the mother/guardian about proper use of the supplement, and will deliver behavior change communications to encourage breastfeeding and proper feeding of complementary foods after 6 months of age. In arms receiving water, sanitation, and/or handwashing interventions, likewise, the promoters deliver technologies and supplies, look after repairs, problem solve, answer questions and doubts, encourage and congratulate the adopters.

Environmental Enteropathy assessment will be conducted by trained staff, including phlebotomists, at two household visits within participating communities. This assessment will take place at the beginning, middle and end of the study. The assessment will take about 8 hours per participant and will occur during two days. On the first day, the field team visit each household to deliver the stool collection kit and provide instructions as well as conduct anthropometry, collect blood, saliva, and hair, and measure heart rate, blood pressure, autonomic function, and skin conductance. On the second day, they will collect stool, urine, saliva and administer the infant food frequency questionnaire. The first visit is expected to take 2 hours and the second visit is expected to take 6 hours.

Stool specimen collection to test for intestinal parasites will be conducted by trained field staff during the final survey. Stool collection will require two visits to each household. The first visit will last about 20 minutes and will involve delivery of the stool collection kit and provision of instructions to the caregiver regarding stool collection. The following day, field staff will return to collect the stool specimen. This visit is expected to take 10 minutes.

The qualitative team will investigate barriers for uptake using unstructured and structured observations, in-depth interviews, and doer/non doer analysis as indicated by the situation. The objective of these investigations is to rapidly identify problems that can be addressed throughout the intervention to ensure a consistent high quality intervention with regular uptake by study participants.

c) Identify any research procedures that are experimental/investigational. Experimental or investigational procedures are treatments or interventions that do not conform to commonly accepted clinical or research practice as may occur in medical, psychological, or educational settings. Note: if the study only involves standard research or clinical procedures, state "N/A."

The element of this study that is not a standard public health interventions is the intensive data collection required to learn from the experience. Most of this data collection involves observation and collection of information that is not particularly culturally sensitive.

The LNS used in the study will be a slightly modified variant of Nutributter, which is a commercially-available supplemental feeding product sold by Nutriset. The specific LNS formulation that we will use has been developed by our nutrition colleagues at UC Davis and has been tested extensively in Bangladesh, Malawi, Ghana and Burkina Faso.

d) If a placebo will be used, provide rationale and explain why active control is not appropriate.

The control group will not receive a placebo.

e) If any type of deception or incomplete disclosure will be used, explain what it will entail, why it is justified, and what the plans are to debrief subjects. See CPHS Guidelines on Deception and Incomplete Disclosure for more information. Any debriefing materials should be included in the Attachments section.

N/A

f) State if audio or video recording will occur and for what purpose (e.g. transcription, coding facial expressions).

Audio, video taping and photography may be done during assessment visits to record interviews and observations. The video, audio records and the photos will be erased from cameras after transferring into the computer. The soft copies will be secured by the computer security system (password protection) until the completion of the study and only the investigator of this study will have the access to open it. All media will be digitized and securely stored in password protected devices for up to 20 years. At that point or once the data is analyzed, the media files will be deleted from the devices. Some photographs or video sections may be used in public presentations and on project websites. Specific consent for these uses will be obtained from study participants. Identifiable information stored electronically on a removable medium or networked computer will be encrypted, as per CPHS policy.

10. Alternatives to Participation

Describe appropriate alternative resources, procedures, courses of treatment, if any, that are available to prospective subjects. If there are no appropriate alternatives to study participation, this should be stated. If the study does not involve treatment/intervention, enter "N/A" here.

Prospective subjects are free to carry on with their current sanitation and hygiene practices, regardless of whether or not they chose to participate in the study. Those who choose not to participate can purchase materials (available outside the study) on their own to improve their child’s nutrition and/or their home environmental conditions.

Protocol Title: Measuring the benefits of sanitation, water quality, handwashing and nutrition interventions for improving health and development in rural Bangladesh

Protocol Status: APPROVED

Date Submitted: 08/18/2014

Approval Period: 09/16/2014-11/03/2016

Important Note: This Print View may not reflect all comments and contingencies for approval. Please check the comments section of the online protocol.
Questions that appear to not have been answered may not have been required for this submission. Please see the system application for more details.

* * * Radiation * * *

11. Ionizing Radiation (e.g. X-ray) and Non-ionizing Radiation (e.g. MRI)

a) Do you intend to use ionizing radioactive materials or ionizing radiation-producing devices in your research (e.g., injectable, oral, x-rays, etc.)? CAUTION: The UCB Radioactive Materials License does not permit human research using radioactive materials or radiation from such materials. — N

If Yes, provide Radiation Use Authorization (RUA) number(s):

Note: The research may not proceed without an RUA. Please visit:
http://ehs.berkeley.edu/rs/133-radiation-use-authorization-rua.html

b) Do you intend to use any non-ionizing radiation sources (laser or magnetic sources) in your research? — N

If Yes, provide Laser Use Registration (LUR) number(s):

And/or Magnetic Inventory number(s):

Note: The research may not proceed without an LUR or Magnetic Inventory Number. Please visit:
http://www.ehs.berkeley.edu/hs/64-laser-safety/67-laser-use-registration-lur.html

c) Describe the source of ionizing radiation or non-ionizing radiation.

**Protocol Title:**	Measuring the benefits of sanitation, water quality, handwashing and nutrition interventions for improving health and development in rural Bangladesh
**Protocol Status:**	APPROVED
**Date Submitted:**	08/18/2014
**Approval Period:**	**09/16/2014-11/03/2016**
**Important Note:**	**This Print View may not reflect all comments and contingencies for approval. Please check the comments section of the online protocol. Questions that appear to not have been answered may not have been required for this submission. Please see the system application for more details.**

* * * Medical Equipment, Investigational Devices * * *

12. Medical Equipment

If the research involves use of medical equipment, explain whether the equipment is approved for marketing and routinely employed in clinical practice.

The following equipment will be used to collect blood and urine samples and for anthropometric measurements. These equipment are all available through major commercial retailers, so our understanding is that they are approved for marketing and routinely employed in clinical practice.

- ECLP Hypodermic needles
- Syringes
- Contact-activated lancets
- Trace metal needles for children
- Reusable tourniquets for children
- Urine specimen collection bags
- Head circumference measuring tape
- ITC Tenderfoot incision devices
- Powder-free nitrile gloves
- Seca 383, 413, 874 digital scales
- Salimetrics children's swabs
- Urine collection jar
- Pediatric urine collector
- Cotton swabs
- ShorrBoard infant/child measuring boards
- Sterile newborn U-bag, 24-hour style
- Prestige medical tourniquets
- 3m Transpore surgical tape

13. Investigational Devices

List in the table below all Investigational Devices to be used on subjects

Investigational Devices

**Protocol Title:**	Measuring the benefits of sanitation, water quality, handwashing and nutrition interventions for improving health and development in rural Bangladesh
**Protocol Status:**	APPROVED
**Date Submitted:**	08/18/2014
**Approval Period:**	**09/16/2014-11/03/2016**
**Important Note:**	**This Print View may not reflect all comments and contingencies for approval. Please check the comments section of the online protocol. Questions that appear to not have been answered may not have been required for this submission. Please see the system application for more details.**

* * * Drugs, Reagents, or Chemicals * * *

14. Drugs, Reagents, or Chemicals

a) List in the table below all investigational drugs, reagents or chemicals to be administered to subjects during this study.

b) List in the table below all commercial drugs, reagents or chemicals to be administered to subjects during this study.

**Generic Drug Name and Synonyms**	Lactulose:mannitol solution
**Source of Drug**	International pharmaceutical supplier
**Manufacturer**	
**If not pre-formulated, where will the material be prepared and by whom?**	ICDDRB, by trained laboratory technicians. The solution will consist of 250 mg of lactulose and 50 mg of mannitol per milliliter of sterile water
**Indications for Use of Drug**	To measure intestinal absorption
**Dosage**	2mL per kilogram body weight
**Route of Administration**	oral
N	Are these new or unapproved uses of these commercially available drugs, reagents, or chemicals? If Yes, you may be required to submit an Investigational New Drug Application (IND) to the FDA. See Off- Label Use of Marketed Drugs.
**IND# (if applicable)**	

**Generic Drug Name and Synonyms**	Aquatabs (chlorine tablets)
**Source of Drug**	in-country supplier
**Manufacturer**	Medentech
**If not pre-formulated, where will the material be prepared and by whom?**	

**Protocol Title:**	Measuring the benefits of sanitation, water quality, handwashing and nutrition interventions for improving health and development in rural Bangladesh
**Protocol Status:**	APPROVED
**Date Submitted:**	08/18/2014
**Approval Period:**	**09/16/2014-11/03/2016**
**Important Note:**	**This Print View may not reflect all comments and contingencies for approval. Please check the comments section of the online protocol. Questions that appear to not have been answered may not have been required for this submission. Please see the system application for more details.**

**Indications for Use of Drug**	water purification
**Dosage**	5 mg per liter of water
**Route of Administration**	drinking water treatment
N	Are these new or unapproved uses of these commercially available drugs, reagents, or chemicals? If Yes, you may be required to submit an Investigational New Drug Application (IND) to the FDA. See Off- Label Use of Marketed Drugs.
**IND# (if applicable)**	

Protocol Title: Measuring the benefits of sanitation, water quality, handwashing and nutrition interventions for improving health and development in rural Bangladesh

Protocol Status: APPROVED

Date Submitted: 08/18/2014

Approval Period: 09/16/2014-11/03/2016

Important Note: This Print View may not reflect all comments and contingencies for approval. Please check the comments section of the online protocol. Questions that appear to not have been answered may not have been required for this submission. Please see the system application for more details.

* * * Risks and Discomforts * * *

15. Risks and Discomforts

a) Describe all known risks and discomforts associated with study procedures, whether physical, psychological, economic or social (e.g., pain, stress, invasion of privacy, breach of confidentiality), noting the likelihood and degree of potential harm.

There is minimal risk of physical, psychological, social, or legal injury from participation in this study.

No severe allergic or other reactions to Nutributter (a product with similar ingredients to the LNS used in this pilot study) were observed in similar studies in Ghana or Bangladesh (conducted by our UC Davis team members), and none are expected in this study. In the LNS arms, when the children are age 6 months we will ask about peanut allergies and will test children with a small sample of the LNS supplement. If a child is allergic, we will not give them LNS but we will retain them in the intention to treat analysis. Community health workers will be trained to tell mothers that they should be aware of any allergic reactions in their children after using the LNS supplement, and in the event of an allergic reaction, not to give the supplement.

The intervention hardware, improved latrines, child potties, handwashing stations, and aluminum water storage vessels are interventions that are widely promoted and used in a variety of contexts. The interventions involve a behavior change component that involves developing communication messages and training local health promoters to deliver these messages. This is a standard approach to public health promotion used throughout Bangladesh and other contexts.

Some aspects of the interventions and data collection activities might be uncomfortable for subjects to discuss, given cultural sensitivities surrounding the topic of defecation. Currently many young children defecate in the open and parents sometimes do not clean this up and sometimes clean it up with a hoe. When using a potty or sani-scoop, parents may be exposed to their children’s feces in somewhat different ways than they previously were.

There is the slight risk of breach of confidentiality. Community members may see study staff entering other baris and homes of their neighbors (usually all family members within a bari), and may overhear interviews. We will make every effort to ensure that household surveys and structured interviews are conducted in privacy.

The measurements for environmental enteropathy involve administration naturally occurring sugars, and small children seem to enjoy the flavor. There is the risk that some children will not like the flavor and will be upset when it is administered. Collection of urine and stool may be uncomfortable to the parent or child. There is also the risk of short-term discomfort and pain during the collection of venous blood and blood spot samples.

b) Discuss measures that will be taken to minimize risks and discomforts to subjects.

We will discuss the objectives of the research with participants as part of delivering the intervention. All participants will be informed of potential discomforts during the consent process, and may decide not to participate at any time during the study. The study team will obtain informed consent from all participants. Individually identified information will be kept confidential.

Potential harms of the study include that people will give time to the study that would be better given to address other issues. We will address this risk by securing informed consent, and clarifying that study participants can drop out at any time, even in the middle of an interview or group discussion.

Discomfort during venous blood draw will be minimized by using trained phlebotomists to collect the specimens. Needles used to collect blood will be disposed of in a safe manner and will not be re-used. Field staff will be carefully trained to collect blood spot samples. We will explain the procedures to the parents and will be available to answer any questions they may have.

We will streamline the data collection procedures as much as possible in order to take as little of the subjects' time as possible. We will make every effort to put subjects at ease during discussions of sensitive topics such as defecation, by using culturally appropriate terminology or euphemisms as possible, and by reminding subjects at the outset that they are free to withdraw from the study activities at any point. It will be made clear during promotion activities that individuals are free to continue their current sanitation practices, and that no one should be coerced to adopt latrine usage. When parents are trained in use of the child's potty, they will be taught the importance of handwashing with soap afterwards.

To minimize the risk of breach of confidentiality, every effort will be made to conduct interviews in the privacy of the participant's home. Data collected during interviews and observations will be kept secure by study staff.

c) If applicable, indicate if a particular study treatment or procedure may involve risks to the subject (or to the embryo or fetus, if the subject is or may become pregnant) that are currently unforeseeable.

N/A

d) If applicable, describe the Data Safety Monitoring Plan (DSMP). NIH may require a DSMP for some projects.

An independent Data Monitoring Committee will be assembled in Bangladesh to monitor adverse events and safety and to advise research investigators. The Committee will include a multi-disciplinary team that will track adverse events in the nutrition arms of the study. This board will meet twice each year, once by phone and once in person.

e) Explain how unanticipated negative outcomes/experiences or serious adverse events will be managed. (NOTE: This may apply in social-behavioral as well as biomedical research, e.g., undue stress or anxiety of subject, breach of confidentiality via loss of laptop computer with study data. Provisions should be made and described here if applicable.)

Protocol Title: Measuring the benefits of sanitation, water quality, handwashing and nutrition interventions for improving health and development in rural Bangladesh

Protocol Status: APPROVED

Date Submitted: 08/18/2014

Approval Period: 09/16/2014-11/03/2016

Important Note: **This Print View may not reflect all comments and contingencies for approval. Please check the comments section of the online protocol. Questions that appear to not have been answered may not have been required for this submission. Please see the system application for more details.**

The interventions used in the study have been used in many other settings and consistently found to be safe, but if hygiene promoters or study staff learn of a severe illness or injury that appears related to study activities, they will inform study supervisors who will inform Dr. Mahbubur Rahman at ICDDRB who will collect information on the event, advise appropriate clinical care for an affected participant, and will notify the Principal Investigator. The event will be documented on an adverse event form and will be submitted to the study investigators at ICDDRB and UC Berkeley. The ICDDRB investigator will report the event to both ICDDRB's Ethical Review Committee and the Data Monitoring Committee.

f) Discuss plans for reporting unanticipated problems involving risks to subjects or others, or serious adverse events, to CPHS. (This applies to all types of research.) See Adverse Event and Unanticipated Problem Reporting.

Adverse events will be reviewed by the UC Berkeley PI. The event will be reported to CPHS if the event 1) is unexpected; 2) is related or possibly related to study participation; AND 3) suggests that the research places subjects or others at a greater risk of harm than was previously known or recognized. If the event is determined by the PI to be reportable, an initial report will be submitted via email to the Director, Research Subject Projection at CPHS. The initial report will be submitted as soon as possible, but no later than 7 days after the PI learns of the event. The initial report will be followed by a formal written report within 14 days of learning of the incident. The formal report will be submitted to CPHS via eProtocol.

g) Describe plans for provision of treatment for study-related injuries, and how costs of injury treatment will be covered. If the study involves more than minimal risk, indicate that the researchers are familiar with and will follow University of California policy in this regard, and will use recommended wording on any consent forms (see CPHS Informed Consent Guidelines).

This study does not involve more than minimal risk. In the LNS arms, health promoters will recommend that caregivers stop using the LNS and notify one of the ICDDRB staff immediately should their child have any adverse reactions shortly after ingesting the supplement (such as vomiting, stomach pain, rash, breathing problems with wheezing). In the event of an adverse reaction, ICDDRB staff will assess the child’s condition and, if necessary, provide transport to the closest medical facility for treatment.

In the anthropometry and enteropathy assessment survey, children who are found to be acutely malnourished based on WHO/Unicef criteria (severely wasted [WHZ < -3] and/or bipedal edema) will be referred to the appropriate existing treatment programs. Children who are found to be infected with intestinal protozoan or helminth parasites will be referred to treatment at the closest health facility. Children with severe anemia (Hb<70 g/L) will be referred for treatment to the closest health facility. Families with children found to have β-thalassemia major will be informed at will be referred to the nearest facility with the capacity for counseling and treatment.

Protocol Title: Measuring the benefits of sanitation, water quality, handwashing and nutrition interventions for improving health and development in rural Bangladesh

Protocol Status: APPROVED

Date Submitted: 08/18/2014

Approval Period: 09/16/2014-11/03/2016

Important Note: This Print View may not reflect all comments and contingencies for approval. Please check the comments section of the online protocol. Questions that appear to not have been answered may not have been required for this submission. Please see the system application for more details.

* * * Benefits, Confidentiality * * *

16. Benefits

Describe any potential benefits to the individual subject, group of subjects, and/or society. If subjects will not benefit directly from study procedures, this should be stated.

NOTE: Do not include compensation/payment of subjects in this section, as remuneration is not considered a "benefit" of participation in research.

Participants will receive the results of all assessments and referrals to appropriate treatment will be made as necessary. Households in the intervention arms will additionally benefit from free sanitation, handwashing and water quality improvements, and nutrient supplements provided by the study. In the long term, the results of this study could benefit other children in Bangladesh and elsewhere by helping us understand the effects of providing nutrition supplements in combination with WASH interventions.

17. Confidentiality and Privacy

NOTE: See CPHS Data Security Policy and Guidelines before completing this section.

a) **If reviewing or accessing Protected Health Information (PHI) from UC Berkeley's Tang Center, Optometry Clinic, Psychology Clinic, Intercollegiate Athletics, or Human Resources for activities preparatory to research, describe the process and confirm that the health information will not be removed from the "covered entity".**

N/A

b) **What identifiable data will you obtain from participants? Note: Audio, photo, and video recordings are generally considered identifiable unless distinguishing features can be successfully masked.**

ICDDR,B field workers will collect participants' names and dates of birth in order to be able to locate them for follow-up data collection. Such data will be collected for the mothers and the target children, as well as their older siblings and neighboring children included in the baseline assessment. We will also collect GPS coordinates of the location of each participating compound. Photographic images used for presentations and websites will not include personal information such as name or GPS coordinates. Field workers will also collect audio and video files as part of the qualitative assessments.

c) **If obtaining existing data/specimens, will you have access to identifiers?**

Protocol Title: Measuring the benefits of sanitation, water quality, handwashing and nutrition interventions for improving health and development in rural Bangladesh

Protocol Status: APPROVED

Date Submitted: 08/18/2014

Approval Period: 09/16/2014-11/03/2016

Important Note: **This Print View may not reflect all comments and contingencies for approval. Please check the comments section of the online protocol. Questions that appear to not have been answered may not have been required for this submission. Please see the system application for more details.**

UC Berkeley scientists will not have access to identifiers with the exception of images to be used in presentations. The ICDDR,B field workers, field managers, and Project Manager for the study will have access to personal identifiers. These names will only be used to guide survey enumerators to the respondents in follow-up survey rounds (no addresses or phone numbers are collected from household survey respondents). Researchers working with the data will only have numerical identification codes for each entry in the database.

d) Explain how the confidentiality of subject information will be maintained. Include:

i. Who will have access to study records/specimens? If the study is subject to FDA regulations, include a statement that the FDA might inspect the records of the study.

The ICDDR,B field workers, field managers, and Project Manager for the study will have access to personal identifiers. Lab scientists working with specimens will not have access to personal identifiers.

ii. How the records will be secured (e.g., password-protected computer, encrypted files, locked cabinet). Response should be consistent with CPHS Data Security Policy.

ICDDRB takes a number of precautions to ensure the confidentiality of all information collected from subjects in the studies it conducts. The majority of data will be collected by PDA (personal digital assistant), which minimizes the risk of loss of confidentiality. GPS coordinates will be recorded digitally as well. The cognitive development and environmental enteropathy teams may use paper-based questionnaires. Interviewers will be trained to keep data confidential. Records of phone numbers collected from health promoters and households selected for structured interviews will be stored in a locked room at the research office in Bangladesh, to which only the project manager at ICDDRB will have access. Blood, stool and urine samples will be labeled with the same numerical ID code used on the household surveys. For audio recordings taken during assessments, the digital output will be transferred to and stored on a secure computer, accessible only to the project manager. The records containing identifiers will be stored in field offices in a locked cabinet. The blood, stool and urine samples will be transported to ICDDR,B and stored in a freezer. Access to the freezer room is restricted by a padlock. Samples will be analyzed within 24 months of collection. Identifiable information stored electronically on a removable medium or networked computer will be encrypted, as per CPHS policy.

Data collection instruments, photographs, and audiotapes will be stored at ICDDRB after study completion. They will be stored securely under lock and key. The study PI and Project Manager will have access. After study completion, these materials will be digitized, and the hardcopy will be destroyed. Digitized materials will be securely stored for an indefinite period of time. Identifiable information (including audio and video files) stored electronically on a removable medium or networked computer will be password protected and encrypted, as per CPHS policy. Digital copies of the data will not contain any identifying information. Hard copies of the survey forms (if any), including those with subject identifiers, will be stored in locked cabinets at ICDDRB.

iii. How long study data will be retained.

Digital copies of the data will be stored indefinitely after the conclusion of the study, for the purposes of additional analysis and informing future research project design. Forms (if any) and digital files containing identifying information will be retained indefinitely for use in a possible follow-up study to assess the long-term health and economic impacts of the interventions. Hard copies of forms which have been digitized will be destroyed at the conclusion of the project. Data resulting from research involving children will be stored for at least 7 years after the child reaches the age of 18 years.

We are interested in holding biological specimens that we collect for 20 years because we envision that these study subjects will be followed in subsequent studies for at least this long so the study population will be a group for whom there is active ongoing scientific interest. The issues that such samples allow us to explore-- how cellular level characteristics influence growth, development and ultimately adult function--are issues where the assays are actively developing in what they can measure. Every year brings substantial breakthroughs. We anticipate that these advances will continue over the next 20 years and permit important insights to be quickly realized without the cost and delay of conducting another decades long longitudinal study. The ICDDR,B WASH Benefits Principal Investigator will be the “gatekeeper” regarding future access and analysis of the stored samples.

iv. When audio/video recordings will be transcribed and when they will be destroyed (if ever).

Audiotapes and videotapes will be securely stored for an indefinite period of time. Identifiable information (including audio and video files) stored electronically on a removable medium or networked computer will be password protected and encrypted, as per CPHS policy.

e) Identifiers should be removed from data/specimens as soon as possible following collection, except in cases where the identifiers are embedded (e.g., voices in audio or faces in video recordings). If data are coded in order to retain a link between the data and identifiable information, explain where the key to the code will be stored, how it will be protected, who will have access to it, and when it will be destroyed.

Identifiers will be removed from the data at the time that the data is sent out for cleaning and analysis. Identifiers will not be destroyed, but will be stored indefinitely at ICDDRB, as we may return at some future date to evaluate long term intervention effectiveness. All personal identifiers other than the household and child identification codes will be removed from paper-based questionnaires prior to digitization. Researchers working with the data have only numerical identification codes for each entry in the database, while the paper forms with respondent names and corresponding study identification codes are stored in a locked room at the research office in Bangladesh. These names are used only to guide survey enumerators to the respondents in follow-up survey rounds (no addresses or phone numbers are collected from household survey respondents). The key to identifiers will be stored in field offices in a locked cabinet and/or password protected server. Only study personnel that require the key to complete the study will have access to the locked cabinet. Once the study is completed, the key to identifiers will be stored at ICDDRB in a locked cabinet and/or secure server.

Protocol Title: Measuring the benefits of sanitation, water quality, handwashing and nutrition interventions for improving health and development in rural Bangladesh

Protocol Status: APPROVED

Date Submitted: 08/18/2014

Approval Period: 09/16/2014-11/03/2016

Important Note: This Print View may not reflect all comments and contingencies for approval. Please check the comments section of the online protocol. Questions that appear to not have been answered may not have been required for this submission. Please see the system application for more details.

f) Describe how identifiable data will be transferred (e.g., courier, mail) or transmitted (e.g., file transfer software, file sharing, email). If transmitted via electronic networks, describe how you will secure the data while in transit (e.g., prior encryption). If not applicable, enter N/A.

Information technology staff directly download the data to desktop computers that are connected to the icddr,b network. The database is password-protected on both PDAs and computers. Data are converted into specified format (STATA, SPSS) and send back to us for error checking and cleaning. Following data cleaning, electronic data are stored in data repository system (DRS) that is connected to the ICDDR,B server and is password-protected as well. Data is encrypted before it is transferred.

g) Will subjects be asked to give permission for release of identifiable data (e.g., for future studies, publications, presentations, etc.), now or in the future? If so, explain here and include appropriate statements in the consent materials. See Media Records Release Form template for guidance.

Participants will be asked for permission to use their images in for public presentations and/or on project websites. No other identifiable data will be included within or along with these photos (i.e. no names or other identifiable data). Appropriate statements have been included in consent materials. Special consent will be obtained to allow the use of this material in public presentations and/or public websites.

h) Explain how subject privacy will be protected (e.g., conducting interviews in a discreet location).

Field workers will ask to conduct interviews inside the household if possible in order to ensure the privacy of the respondent and the compound members. If non-compound members are present, the field worker will ask them to leave during the interview to ensure the privacy of the respondent. Compound members are often present, but for sensitive questions, field workers will politely ask them to leave so the interview can be conducted in private.

**Protocol Title:**	Measuring the benefits of sanitation, water quality, handwashing and nutrition interventions for improving health and development in rural Bangladesh
**Protocol Status:**	APPROVED
**Date Submitted:**	08/18/2014
**Approval Period:**	**09/16/2014-11/03/2016**
**Important Note:**	**This Print View may not reflect all comments and contingencies for approval. Please check the comments section of the online protocol. Questions that appear to not have been answered may not have been required for this submission. Please see the system application for more details.**

* * * Potential Financial Conflict of Interest * * *

18. Potential Financial Conflict of Interest

Individuals who have independent roles in projects and who are responsible for the design, analysis, conduct, or reporting of the results of research performed (or to be performed) under a human subjects protocol must disclose whether or not they have a financial interest in or association with the sponsor or the company supplying materials, drugs, or devices for the project. This checklist pertains to the entire project team working under the protocol. Any individual who has a conflict must comply with University regulations and procedures for disclosure of financial conflict of interest.

See Conflict of Interest Committee Website for more information.

Please answer the following questions:

Does any member of the project team (defined as UCB or non-UCB personnel working under the protocol) with substantive responsibility for the design, conduct, or reporting of activities under the protocol, or any member of their immediate family (defined as spouse, dependent child or registered domestic partner) have any of the following relationships with the non-UC entity financing the research to be done under the protocol or the non-UC entity supplying materials, drugs or devices being tested under the protocol:

1. N **Positions of management (e.g., board member, scientific advisor, director, officer, partner, trustee, employee, consultant).**
2. N **Equity interest (e.g., stock, stock options, investment, or other ownership).**
3. N **Rights to a pending patent application or issued patent to any invention(s), or license rights or copyright for software that has a direct relationship to the project proposed.**

If the answer to any of the above is Yes, then each individual with any "Yes" response (s) must submit a Human Subjects Financial Conflict of Interest Form DIRECTLY to the Conflict of Interest (COI) Committee for a separate review.

NOTE: When review by the COI Committee is required, CPHS approval or exemption of protocols will be contingent upon the disclosure and resolution of all financial conflicts of interest, as determined by the COI Committee.

**Protocol Title:**	Measuring the benefits of sanitation, water quality, handwashing and nutrition interventions for improving health and development in rural Bangladesh
**Protocol Status:**	APPROVED
**Date Submitted:**	08/18/2014
**Approval Period:**	**09/16/2014-11/03/2016**
**Important Note:**	**This Print View may not reflect all comments and contingencies for approval. Please check the comments section of the online protocol. Questions that appear to not have been answered may not have been required for this submission. Please see the system application for more details.**

* * * Informed Consent * * *

19. Informed Consent

Add the consent documents and/or waivers needed for this research using the table at the bottom of the page. Any foreign language versions should also be added. You will be asked to provide relevant background information for each consent document or waiver. The various consent/waiver options are described below.

Note: DO NOT include child assent documents, parent permission documents or waivers here (these are addressed in the next section).

Altered and Unsigned Consent - A consent document that has omitted required information and does not include a place for a participant's signature. This means that CPHS is being asked to waive one or more elements of consent in addition to the requirement for documented consent.

Altered Consent Form - A consent form that has omitted required information. This means that the CPHS is asked to waive one or more required elements of informed consent. For example, if the purpose of the study will not be disclosed to participants in order to avoid bias, this option should be selected because disclosure of the "purpose" is a required element of informed consent. The form must include a signature line and date line for the individual to sign if he or she agrees to participate.

Consent Form - A standard consent document that embodies all of the required information (elements of informed consent) designed to help an individual make an informed decision about whether or not to participate in the research. The form must include a signature line and date line for the individual to sign if he or she agrees to participate. The Consent Form can also be presented as a "short form" document stating that the required elements of informed consent have been presented orally to the participant. When the short form method is used, a "summary" of the information that is presented to the participant must also be provided for CPHS approval and there must be an impartial witness to the oral presentation. The witness must sign the summary as well as the short form and the participant must sign the summary. The "short form" method may be used in circumstances where oral presentation of consent is preferable or necessary, e.g., subjects are illiterate in English or their native language.

Consent Waiver - No consent will be sought at all. This means that the CPHS is asked to waive the requirement for informed consent. This option is often appropriate for research that involves use of existing data or samples

Unsigned Consent - A document that embodies all of the required information (elements of informed consent), but does not include a place for a participant to indicate with a signature that he or she agrees to

take part in the research. This means that the CPHS is asked to waive the requirement for documented (signed) consent. For example, if consent will be obtained verbally or using a button on the web, this option should be selected.

•Informed Consent Guidelines, Templates and Sample Forms

•Informed Consent Policies and Procedures

•Consent Builder: Online Tool for Creating Consent Forms

Informed Consent

Consent/Waiver Description	Consent Type	Consent Document

Informed Consent

Consent/Waiver Description (e.g. Consent for Group A, Waiver for Group B, Surrogate Consent for Group C)	Consent 1i		
Consent Type	Consent Form		
Attach Consent Document (in PDF format)	X	Consent Document	2011-09-3652_Colford_1i-Consent-Parasite-v20-English

Explain how, where, when, and by whom informed consent will be obtained. If any vulnerable subject groups are involved, discuss relevant considerations. Note: If attaching multiple consent forms and consent process has already been described for another consent form, simply refer to the other form (e.g., consent process is the same as for Group A).

ICDDR,B field workers will approach eligible compounds and ask to speak with the pregnant woman/mother and head of compound. The study will be described, and participant involvement described. If the compound agrees to participate, the consent process will be completed. If a potential respondent is not able to read the consent form, field workers will suggest that they invite a witness to help read the form with them so that they can be certain that they agree to participate in the procedures in the study. If a witness is present, both the witness and respondent will be asked to provide a thumb print/signature.

Consent/Waiver Description (e.g. Consent for Group A, Waiver for Group B, Surrogate Consent for Group C)	Consent 2i - Bengali		
Consent Type	Consent Form		
Attach Consent Document (in PDF format)	X	Consent Document	2011-09-3652_Colford_2i-Consent-Parasite-v20-Bengali

**Protocol Title:**	Measuring the benefits of sanitation, water quality, handwashing and nutrition interventions for improving health and development in rural Bangladesh
**Protocol Status:**	APPROVED
**Date Submitted:**	08/18/2014
**Approval Period:**	**09/16/2014-11/03/2016**
**Important Note:**	**This Print View may not reflect all comments and contingencies for approval. Please check the comments section of the online protocol. Questions that appear to not have been answered may not have been required for this submission. Please see the system application for more details.**

Explain how, where, when, and by whom informed consent will be obtained. If any vulnerable subject groups are involved, discuss relevant considerations. Note: If attaching multiple consent forms and consent process has already been described for another consent form, simply refer to the other form (e.g., consent process is the same as for Group A).

ICDDR,B field workers will approach eligible compounds and ask to speak with the pregnant woman/mother and head of compound. The study will be described, and participant involvement described. If the compound agrees to participate, the consent process will be completed.

Consent/Waiver Description (e.g. Consent for Group A, Waiver for Group B, Surrogate Consent for Group C)	Consent 1k1		
Consent Type	Consent Form		
Attach Consent Document (in PDF format)	X	Consent Document	2011-09-3652_Colford_1k1-Consent-EnvAssess-v19-English

Explain how, where, when, and by whom informed consent will be obtained. If any vulnerable subject groups are involved, discuss relevant considerations. Note: If attaching multiple consent forms and consent process has already been described for another consent form, simply refer to the other form (e.g., consent process is the same as for Group A).

ICDDR,B field workers will approach eligible compounds and ask to speak with the pregnant woman/mother and head of compound. The study will be described, and participant involvement described. If the compound agrees to participate, the consent process will be completed.

Consent/Waiver Description (e.g. Consent for Group A, Waiver for Group B, Surrogate Consent for Group C)	Consent 2k1 - Bengali		
Consent Type	Consent Form		
Attach Consent Document (in PDF format)	X	Consent Document	2011-09-3652_Colford_2k1-Consent-EnvAssess-v19-Bengali

Explain how, where, when, and by whom informed consent will be obtained. If any vulnerable subject groups are involved, discuss relevant considerations. Note: If attaching multiple consent forms and consent process has already been described for another consent form, simply refer to the other form (e.g., consent process is the same as for Group A).

ICDDR,B field workers will approach eligible compounds and ask to speak with the pregnant woman/mother and head of compound. The study will be described, and participant involvement described. If the compound agrees to participate, the consent process will be completed.

Consent/Waiver Description (e.g. Consent for Group A, Waiver for Group B, Surrogate Consent for Group C)	Consent 1k3		
Consent Type	Consent Form		
Attach Consent Document (in PDF format)	X	Consent Document	2011-09-3652_Colford_1k3-Consent-EnvAssess-hygiene-v19-English

Explain how, where, when, and by whom informed consent will be obtained. If any vulnerable subject groups are involved, discuss relevant considerations. Note: If attaching multiple consent forms and consent process has already been described for another consent form, simply refer to the other form (e.g., consent process is the same as for Group A).

ICDDR,B field workers will approach eligible compounds and ask to speak with the pregnant woman/mother and head of compound. The study will be described, and participant involvement described. If the compound agrees to participate, the consent process will be completed.

Consent/Waiver Description (e.g. Consent for Group A, Waiver for Group B, Surrogate Consent for Group C)	Consent 2k3 - Bengali		
Consent Type	Consent Form		
Attach Consent Document (in PDF format)	X	Consent Document	2011-09-3652_Colford_2k3-Consent-EnvAssess-hygiene-v19-Bengali

Explain how, where, when, and by whom informed consent will be obtained. If any vulnerable subject groups are involved, discuss relevant considerations. Note: If attaching multiple consent forms and consent process has already been described for another consent form, simply refer to the other form (e.g., consent process is the same as for Group A).

ICDDR,B field workers will approach eligible compounds and ask to speak with the pregnant woman/mother and head of compound. The study will be described, and participant involvement described. If the compound agrees to participate, the consent process will be completed.

Consent/Waiver Description (e.g. Consent for Group A, Waiver for Group B, Surrogate Consent for Group C)	Consent 1l		
Consent Type	Consent Form		
Attach Consent Document (in PDF format)	X	Consent Document	2011-09-3652_Colford_1l-Consent-5hr-SO-v18-English

Explain how, where, when, and by whom informed consent will be obtained. If any vulnerable subject groups are involved, discuss relevant considerations. Note: If attaching multiple consent forms and consent process has already been described for another consent form, simply refer to the other form (e.g., consent process is the same as for Group A).

**Protocol Title:**	Measuring the benefits of sanitation, water quality, handwashing and nutrition interventions for improving health and development in rural Bangladesh
**Protocol Status:**	APPROVED
**Date Submitted:**	08/18/2014
**Approval Period:**	**09/16/2014-11/03/2016**
**Important Note:**	**This Print View may not reflect all comments and contingencies for approval. Please check the comments section of the online protocol. Questions that appear to not have been answered may not have been required for this submission. Please see the system application for more details.**

ICDDR,B field workers will approach eligible compounds and ask to speak with the pregnant woman/mother and head of compound. The study will be described, and participant involvement described. If the compound agrees to participate, the consent process will be completed.

Consent/Waiver Description (e.g. Consent for Group A, Waiver for Group B, Surrogate Consent for Group C)	Consent 2l - Bengali		
Consent Type	Consent Form		
Attach Consent Document (in PDF format)	X	Consent Document	2011-09-3652_Colford_2l-Consent-5hr-SO-v18-Bengali

Explain how, where, when, and by whom informed consent will be obtained. If any vulnerable subject groups are involved, discuss relevant considerations. Note: If attaching multiple consent forms and consent process has already been described for another consent form, simply refer to the other form (e.g., consent process is the same as for Group A).

ICDDR,B field workers will approach eligible compounds and ask to speak with the pregnant woman/mother and head of compound. The study will be described, and participant involvement described. If the compound agrees to participate, the consent process will be completed.

Consent/Waiver Description (e.g. Consent for Group A, Waiver for Group B, Surrogate Consent for Group C)	Consent 1n		
Consent Type	Consent Form		
Attach Consent Document (in PDF format)	X	Consent Document	2011-09-3652_Colford_1n-Consent-Lead exposure-v17-English

Explain how, where, when, and by whom informed consent will be obtained. If any vulnerable subject groups are involved, discuss relevant considerations. Note: If attaching multiple consent forms and consent process has already been described for another consent form, simply refer to the other form (e.g., consent process is the same as for Group A).

ICDDR,B field workers will approach eligible compounds and ask to speak with the pregnant woman/mother and head of compound. The study will be described, and participant involvement described. If the compound agrees to participate, the consent process will be completed.

Consent/Waiver Description (e.g. Consent for Group A, Waiver for Group B, Surrogate Consent for Group C)	Consent 2n - Bengali
Consent Type	Consent Form

Protocol Title: Measuring the benefits of sanitation, water quality, handwashing and nutrition interventions for improving health and development in rural Bangladesh

Protocol Status: APPROVED

Date Submitted: 08/18/2014

Approval Period: **09/16/2014-11/03/2016**

Important Note: **This Print View may not reflect all comments and contingencies for approval. Please check the comments section of the online protocol. Questions that appear to not have been answered may not have been required for this submission. Please see the system application for more details.**

Attach Consent Document (in PDF format)	X	Consent Document	2011-09-3652_Colford_2n-Consent-Lead exposure-v17-Bengali

Explain how, where, when, and by whom informed consent will be obtained. If any vulnerable subject groups are involved, discuss relevant considerations. Note: If attaching multiple consent forms and consent process has already been described for another consent form, simply refer to the other form (e.g., consent process is the same as for Group A).

ICDDR,B field workers will approach eligible compounds and ask to speak with the pregnant woman/mother and head of compound. The study will be described, and participant involvement described. If the compound agrees to participate, the consent process will be completed.

Consent/Waiver Description (e.g. Consent for Group A, Waiver for Group B, Surrogate Consent for Group C): Consent 1o

Consent Type	Consent Form		
Attach Consent Document (in PDF format)	X	Consent Document	2011-09-3652_Colford_1o-Consent-Agrochemical-v17-English

Explain how, where, when, and by whom informed consent will be obtained. If any vulnerable subject groups are involved, discuss relevant considerations. Note: If attaching multiple consent forms and consent process has already been described for another consent form, simply refer to the other form (e.g., consent process is the same as for Group A).

ICDDR,B field workers will approach eligible compounds and ask to speak with the pregnant woman/mother and head of compound. The study will be described, and participant involvement described. If the compound agrees to participate, the consent process will be completed.

Consent/Waiver Description (e.g. Consent for Group A, Waiver for Group B, Surrogate Consent for Group C): Consent 2o - Bengali

Consent Type	Consent Form		
Attach Consent Document (in PDF format)	X	Consent Document	2011-09-3652_Colford_2o-Consent-Agrochemical-v17-Bengali

Explain how, where, when, and by whom informed consent will be obtained. If any vulnerable subject groups are involved, discuss relevant considerations. Note: If attaching multiple consent forms and consent process has already been described for another consent form, simply refer to the other form (e.g., consent process is the same as for Group A).

ICDDR,B field workers will approach eligible compounds and ask to speak with the pregnant woman/mother and head of compound. The study will be described, and participant involvement described. If the compound agrees to participate, the consent process will be completed.

Consent/Waiver Description (e.g. Consent for Group A, Waiver for Group B, Surrogate Consent for Group C)	Consent 1p

Consent Type	Consent Form		
Attach Consent Document (in PDF format)	X	Consent Document	2011-09-3652_Colford_1p-Consent-Lead case control-v17-English

Explain how, where, when, and by whom informed consent will be obtained. If any vulnerable subject groups are involved, discuss relevant considerations. Note: If attaching multiple consent forms and consent process has already been described for another consent form, simply refer to the other form (e.g., consent process is the same as for Group A).

ICDDR,B field workers will approach eligible compounds and ask to speak with the pregnant woman/mother and head of compound. The study will be described, and participant involvement described. If the compound agrees to participate, the consent process will be completed.

Consent/Waiver Description (e.g. Consent for Group A, Waiver for Group B, Surrogate Consent for Group C)	Consent 2p - Bengali

Consent Type	Consent Form		
Attach Consent Document (in PDF format)	X	Consent Document	2011-09-3652_Colford_2p-Consent-Lead case control-v17-Bengali

Explain how, where, when, and by whom informed consent will be obtained. If any vulnerable subject groups are involved, discuss relevant considerations. Note: If attaching multiple consent forms and consent process has already been described for another consent form, simply refer to the other form (e.g., consent process is the same as for Group A).

ICDDR,B field workers will approach eligible compounds and ask to speak with the pregnant woman/mother and head of compound. The study will be described, and participant involvement described. If the compound agrees to participate, the consent process will be completed.

Consent/Waiver Description (e.g. Consent for Group A, Waiver for Group B, Surrogate Consent for Group C)	Consent 1r

Consent Type	Consent Form		
Attach Consent Document (in PDF format)	X	Consent Document	2011-09-3652_Colford_1r-Consent-Structured observation with video-v17-English

---

Protocol Title: Measuring the benefits of sanitation, water quality, handwashing and nutrition interventions for improving health and development in rural Bangladesh

Protocol Status: APPROVED

Date Submitted: 08/18/2014

Approval Period: **09/16/2014-11/03/2016**

Important Note: **This Print View may not reflect all comments and contingencies for approval. Please check the comments section of the online protocol. Questions that appear to not have been answered may not have been required for this submission. Please see the system application for more details.**

Explain how, where, when, and by whom informed consent will be obtained. If any vulnerable subject groups are involved, discuss relevant considerations. Note: If attaching multiple consent forms and consent process has already been described for another consent form, simply refer to the other form (e.g., consent process is the same as for Group A).

ICDDR,B field workers will approach eligible compounds and ask to speak with the pregnant woman/mother and head of compound. The study will be described, and participant involvement described. If the compound agrees to participate, the consent process will be completed.

Consent/Waiver Description (e.g. Consent for Group A, Waiver for Group B, Surrogate Consent for Group C)	Consent 2r - Bengali		
Consent Type	Consent Form		
Attach Consent Document (in PDF format)	X	Consent Document	2011-09-3652_Colford_2r-Consent-Structured observation with video-v17-Bengali

Explain how, where, when, and by whom informed consent will be obtained. If any vulnerable subject groups are involved, discuss relevant considerations. Note: If attaching multiple consent forms and consent process has already been described for another consent form, simply refer to the other form (e.g., consent process is the same as for Group A).

ICDDR,B field workers will approach eligible compounds and ask to speak with the pregnant woman/mother and head of compound. The study will be described, and participant involvement described. If the compound agrees to participate, the consent process will be completed.

Consent/Waiver Description (e.g. Consent for Group A, Waiver for Group B, Surrogate Consent for Group C)	Consent 1s		
Consent Type	Consent Form		
Attach Consent Document (in PDF format)	X	Consent Document	2011-09-3652_Colford_1s-Consent-In depth interview environmental assessment-v17-English

Explain how, where, when, and by whom informed consent will be obtained. If any vulnerable subject groups are involved, discuss relevant considerations. Note: If attaching multiple consent forms and consent process has already been described for another consent form, simply refer to the other form (e.g., consent process is the same as for Group A).

ICDDR,B field workers will approach eligible compounds and ask to speak with the pregnant woman/mother and head of compound. The study will be described, and participant involvement described. If the compound agrees to participate, the consent process will be completed.

Consent/Waiver Description (e.g. Consent for Group A, Waiver for Group B, Surrogate Consent for Group C)	Consent 2s - Bengali		
Consent Type	Consent Form		
Attach Consent Document (in PDF format)	X	Consent Document	2011-09-3652_Colford_2s-Consent-In depth interview environmental assessment-v17-Bengali

Explain how, where, when, and by whom informed consent will be obtained. If any vulnerable subject groups are involved, discuss relevant considerations. Note: If attaching multiple consent forms and consent process has already been described for another consent form, simply refer to the other form (e.g., consent process is the same as for Group A).

ICDDR,B field workers will approach eligible compounds and ask to speak with the pregnant woman/mother and head of compound. The study will be described, and participant involvement described. If the compound agrees to participate, the consent process will be completed.

Consent/Waiver Description (e.g. Consent for Group A, Waiver for Group B, Surrogate Consent for Group C)	Consent 1t		
Consent Type	Consent Form		
Attach Consent Document (in PDF format)	X	Consent Document	2011-09-3652_Colford_1t-Consent-Focus-Group-Discussion-v17-English

Explain how, where, when, and by whom informed consent will be obtained. If any vulnerable subject groups are involved, discuss relevant considerations. Note: If attaching multiple consent forms and consent process has already been described for another consent form, simply refer to the other form (e.g., consent process is the same as for Group A).

ICDDR,B field workers will approach eligible compounds and ask to speak with the pregnant woman/mother and head of compound. The study will be described, and participant involvement described. If the compound agrees to participate, the consent process will be completed.

Consent/Waiver Description (e.g. Consent for Group A, Waiver for Group B, Surrogate Consent for Group C)	Consent 2t - Bengali		
Consent Type	Consent Form		
Attach Consent Document (in PDF format)	X	Consent Document	2011-09-3652_Colford_2t-Consent-Focus-Group-Discussion-v17-Bengali

---

**Protocol Title:**	Measuring the benefits of sanitation, water quality, handwashing and nutrition interventions for improving health and development in rural Bangladesh
**Protocol Status:**	APPROVED
**Date Submitted:**	08/18/2014
**Approval Period:**	**09/16/2014-11/03/2016**
**Important Note:**	**This Print View may not reflect all comments and contingencies for approval. Please check the comments section of the online protocol. Questions that appear to not have been answered may not have been required for this submission. Please see the system application for more details.**

Explain how, where, when, and by whom informed consent will be obtained. If any vulnerable subject groups are involved, discuss relevant considerations. Note: If attaching multiple consent forms and consent process has already been described for another consent form, simply refer to the other form (e.g., consent process is the same as for Group A).

ICDDR,B field workers will approach eligible compounds and ask to speak with the pregnant woman/mother and head of compound. The study will be described, and participant involvement described. If the compound agrees to participate, the consent process will be completed.

Consent/Waiver Description (e.g. Consent for Group A, Waiver for Group B, Surrogate Consent for Group C)	Consent 1u		
Consent Type	Consent Form		
Attach Consent Document (in PDF format)	X	Consent Document	2011-09-3652_Colford_1u-Consent-Longitudinal-EnvAssess-v20-English

Explain how, where, when, and by whom informed consent will be obtained. If any vulnerable subject groups are involved, discuss relevant considerations. Note: If attaching multiple consent forms and consent process has already been described for another consent form, simply refer to the other form (e.g., consent process is the same as for Group A).

ICDDR,B field workers will approach eligible compounds and ask to speak with the pregnant woman/mother and head of compound. The study will be described, and participant involvement described. If the compound agrees to participate, the consent process will be completed. If a potential respondent is not able to read the consent form, field workers will suggest that they invite a witness to help read the form with them so that they can be certain that they agree to participate in the procedures in the study. If a witness is present, both the witness and respondent will be asked to provide a thumb print/signature.

Consent/Waiver Description (e.g. Consent for Group A, Waiver for Group B, Surrogate Consent for Group C)	Consent 2u-Bengali		
Consent Type	Consent Form		
Attach Consent Document (in PDF format)	X	Consent Document	2011-09-3652_Colford_2u-Consent-Longitudinal-EnvAssess-v20-Bengali

Explain how, where, when, and by whom informed consent will be obtained. If any vulnerable subject groups are involved, discuss relevant considerations. Note: If attaching multiple consent forms and consent process has already been described for another consent form, simply refer to the other form (e.g., consent process is the same as for Group A).

ICDDR,B field workers will approach eligible compounds and ask to speak with the pregnant woman/mother and head of compound. The study will be described, and participant involvement described. If the compound agrees to participate, the consent process will be completed.

**Protocol Title:**	Measuring the benefits of sanitation, water quality, handwashing and nutrition interventions for improving health and development in rural Bangladesh
**Protocol Status:**	APPROVED
**Date Submitted:**	08/18/2014
**Approval Period:**	**09/16/2014-11/03/2016**
**Important Note:**	**This Print View may not reflect all comments and contingencies for approval. Please check the comments section of the online protocol. Questions that appear to not have been answered may not have been required for this submission. Please see the system application for more details.**

Consent/Waiver Description (e.g. Consent for Group A, Waiver for Group B, Surrogate Consent for Group C)	Consent 1k2		
Consent Type	Consent Form		
Attach Consent Document (in PDF format)	X	Consent Document	2011-09-3652_Colford_1k2-Consent-EnvAssess-water-v19-English

Explain how, where, when, and by whom informed consent will be obtained. If any vulnerable subject groups are involved, discuss relevant considerations. Note: If attaching multiple consent forms and consent process has already been described for another consent form, simply refer to the other form (e.g., consent process is the same as for Group A).

ICDDR,B field workers will approach eligible compounds and ask to speak with the pregnant woman/mother and head of compound. The study will be described, and participant involvement described. If the compound agrees to participate, the consent process will be completed.

Consent/Waiver Description (e.g. Consent for Group A, Waiver for Group B, Surrogate Consent for Group C)	Consent 2k2 - Bengali		
Consent Type	Consent Form		
Attach Consent Document (in PDF format)	X	Consent Document	2011-09-3652_Colford_2k2-Consent-EnvAssess-water-v19-Bengali

Explain how, where, when, and by whom informed consent will be obtained. If any vulnerable subject groups are involved, discuss relevant considerations. Note: If attaching multiple consent forms and consent process has already been described for another consent form, simply refer to the other form (e.g., consent process is the same as for Group A).

ICDDR,B field workers will approach eligible compounds and ask to speak with the pregnant woman/mother and head of compound. The study will be described, and participant involvement described. If the compound agrees to participate, the consent process will be completed.

Consent/Waiver Description (e.g. Consent for Group A, Waiver for Group B, Surrogate Consent for Group C)	Consent 1v		
Consent Type	Consent Form		
Attach Consent Document (in PDF format)	X	Consent Document	2011-09-3652_Colford_1v-Child cognitive development-v18-English

---

Explain how, where, when, and by whom informed consent will be obtained. If any vulnerable subject groups are involved, discuss relevant considerations. Note: If attaching multiple consent forms and consent process has already been described for another consent form, simply refer to the other form (e.g., consent process is the same as for Group A).

ICDDR,B field workers will approach eligible compounds and ask to speak with the pregnant woman/mother and head of compound. The study will be described, and participant involvement described. If the compound agrees to participate, the consent process will be completed.

Consent/Waiver Description (e.g. Consent for Group A, Waiver for Group B, Surrogate Consent for Group C)	Consent 2v-Bengali		
Consent Type	Consent Form		
Attach Consent Document (in PDF format)	X	Consent Document	2011-09-3652_Colford_2v-Child cognitive development-v18-Bengali

Explain how, where, when, and by whom informed consent will be obtained. If any vulnerable subject groups are involved, discuss relevant considerations. Note: If attaching multiple consent forms and consent process has already been described for another consent form, simply refer to the other form (e.g., consent process is the same as for Group A).

ICDDR,B field workers will approach eligible compounds and ask to speak with the pregnant woman/mother and head of compound. The study will be described, and participant involvement described. If the compound agrees to participate, the consent process will be completed.

Consent/Waiver Description (e.g. Consent for Group A, Waiver for Group B, Surrogate Consent for Group C)	Consent 1w		
Consent Type	Consent Form		
Attach Consent Document (in PDF format)	X	Consent Document	2011-09-3652_Colford_1w-Consent-Spillover compounds-v19-English

Explain how, where, when, and by whom informed consent will be obtained. If any vulnerable subject groups are involved, discuss relevant considerations. Note: If attaching multiple consent forms and consent process has already been described for another consent form, simply refer to the other form (e.g., consent process is the same as for Group A).

ICDDR,B field workers will approach eligible compounds and ask to speak with the pregnant woman/mother and head of compound. The study will be described, and participant involvement described. If the compound agrees to participate, the consent process will be completed.

**Protocol Title:**	Measuring the benefits of sanitation, water quality, handwashing and nutrition interventions for improving health and development in rural Bangladesh
**Protocol Status:**	APPROVED
**Date Submitted:**	08/18/2014
**Approval Period:**	**09/16/2014-11/03/2016**
**Important Note:**	**This Print View may not reflect all comments and contingencies for approval. Please check the comments section of the online protocol. Questions that appear to not have been answered may not have been required for this submission. Please see the system application for more details.**

Consent/Waiver Description (e.g. Consent for Group A, Waiver for Group B, Surrogate Consent for Group C)	Consent 2w - Bengali		
Consent Type	Consent Form		
Attach Consent Document (in PDF format)	X	Consent Document	2011-09-3652_Colford_2w-Consent-Spillover compounds-v19-Bengali

Explain how, where, when, and by whom informed consent will be obtained. If any vulnerable subject groups are involved, discuss relevant considerations. Note: If attaching multiple consent forms and consent process has already been described for another consent form, simply refer to the other form (e.g., consent process is the same as for Group A).

ICDDR,B field workers will approach eligible compounds and ask to speak with the pregnant woman/mother and head of compound. The study will be described, and participant involvement described. If the compound agrees to participate, the consent process will be completed.

Consent/Waiver Description (e.g. Consent for Group A, Waiver for Group B, Surrogate Consent for Group C)	Consent 1j		
Consent Type	Consent Form		
Attach Consent Document (in PDF format)	X	Consent Document	2011-09-3652_Colford_1j-Consent-EE-v20-English

Explain how, where, when, and by whom informed consent will be obtained. If any vulnerable subject groups are involved, discuss relevant considerations. Note: If attaching multiple consent forms and consent process has already been described for another consent form, simply refer to the other form (e.g., consent process is the same as for Group A).

ICDDR,B field workers will approach eligible compounds and ask to speak with the pregnant woman/mother and head of compound. The study will be described, and participant involvement described. If the compound agrees to participate, the consent process will be completed. If a potential respondent is not able to read the consent form, field workers will suggest that they invite a witness to help read the form with them so that they can be certain that they agree to participate in the procedures in the study. If a witness is present, both the witness and respondent will be asked to provide a thumb print/signature.

Consent/Waiver Description (e.g. Consent for Group A, Waiver for Group B, Surrogate Consent for Group C)	Consent 2j - Bengali		
Consent Type	Consent Form		
Attach Consent Document (in PDF format)	X	Consent Document	2011-09-3652_Colford_2j-Consent-EE-v20-Bengali

---

Explain how, where, when, and by whom informed consent will be obtained. If any vulnerable subject groups are involved, discuss relevant considerations. Note: If attaching multiple consent forms and consent process has already been described for another consent form, simply refer to the other form (e.g., consent process is the same as for Group A).

ICDDR,B field workers will approach eligible compounds and ask to speak with the pregnant woman/mother and head of compound. The study will be described, and participant involvement described. If the compound agrees to participate, the consent process will be completed.

Consent/Waiver Description (e.g. Consent for Group A, Waiver for Group B, Surrogate Consent for Group C)	Consent 1z		
Consent Type	Consent Form		
Attach Consent Document (in PDF format)	X	Consent Document	2011-09-3652_Colford_1z-Consent-Microbiome-v20-English

Explain how, where, when, and by whom informed consent will be obtained. If any vulnerable subject groups are involved, discuss relevant considerations. Note: If attaching multiple consent forms and consent process has already been described for another consent form, simply refer to the other form (e.g., consent process is the same as for Group A).

ICDDR,B field workers will approach eligible compounds and ask to speak with the pregnant woman/mother and head of compound. The study will be described, and participant involvement described. If the compound agrees to participate, the consent process will be completed. If a potential respondent is not able to read the consent form, field workers will suggest that they invite a witness to help read the form with them so that they can be certain that they agree to participate in the procedures in the study. If a witness is present, both the witness and respondent will be asked to provide a thumb print/signature.

Consent/Waiver Description (e.g. Consent for Group A, Waiver for Group B, Surrogate Consent for Group C)	Consent 2z - Bengali		
Consent Type	Consent Form		
Attach Consent Document (in PDF format)	X	Consent Document	2011-09-3652_Colford_2z-Consent-Microbiome-v20-Bengali

Explain how, where, when, and by whom informed consent will be obtained. If any vulnerable subject groups are involved, discuss relevant considerations. Note: If attaching multiple consent forms and consent process has already been described for another consent form, simply refer to the other form (e.g., consent process is the same as for Group A).

ICDDR,B field workers will approach eligible compounds and ask to speak with the pregnant woman/mother and head of compound. The study will be described, and participant involvement described. If the compound agrees to participate, the consent process will be completed.

Protocol Title: Measuring the benefits of sanitation, water quality, handwashing and nutrition interventions for improving health and development in rural Bangladesh

Protocol Status: APPROVED

Date Submitted: 08/18/2014

Approval Period: 09/16/2014-11/03/2016

Important Note: This Print View may not reflect all comments and contingencies for approval. Please check the comments section of the online protocol.
Questions that appear to not have been answered may not have been required for this submission. Please see the system application for more details.

---

* * * Child Assent & Parent Permission * * *

20. Child Assent and Parent/Guardian Permission

Add each assent document, parent/guardian permission document, and/or waiver needed for this research using the table at the bottom of the page. Any foreign language versions should also be added. You will be asked to provide relevant background information for each assent, permission, or waiver. The various assent, permission, and waiver options are described below.

Altered and Unsigned Parent/Guardian Permission Form - A parent permission document that has omitted required information (elements) and does not include a place for a parent to indicate with a signature that he or she agrees to permit the child's participation. This means that CPHS is being asked to waive one or more elements of consent in addition to the requirement for documented consent.

Altered Parent/Guardian Permission Form - A permission form that has omitted required information (elements). This means that the CPHS is asked to waive one or more required elements of informed consent. However, the form must include signature and date lines for the parent(s)/guardian(s) to sign if the child is permitted to take part in the research.

Assent Document - A form or script of the information that will be conveyed to the child about the study. In general, researchers must obtain the affirmative agreement of children ages seven years and older for their participation. Assent forms should be written at a level understandable to the child. If the study includes a broad age range of children, more than one assent form may be needed (i.e., an assent form suitable for a 15 year old is not usually suitable for a 7 year old child).

Assent Waiver - No child assent will be sought at all. This means that CPHS is asked to waive the requirement for child assent. Among other circumstances, this option is appropriate when the capability of the child to understand the research is too limited or when the research holds out a prospect of direct benefit that is important to the health or well being of the child.

Parent/Guardian Permission Form - A document that embodies all of the required information (elements of informed consent) designed to help the parent/guardian of a child make an informed decision about whether or not to permit the child's participation in the research. The form must include signature and date lines for the parent(s)/guardian(s) to sign if the child is permitted to take part in the research.

Permission Waiver - No parent/guardian permission will be sought at all. This means that the CPHS is asked to waive the requirement for parent/guardian permission. This option, for example, is often appropriate for research designed to study conditions in children or a study population for which parental permission is not a reasonable requirement to protect the children (e.g., neglected or abused children).

Unsigned Parent/Guardian Permission - A parent permission document that embodies all of the required information (elements of informed consent), but does not include a place for a parent to indicate with a signature that he or she agrees to permit the child's participation. This means that the CPHS is asked to waive the requirement for documented (signed) consent.

•Child Assent and Parent Permission Guidelines, Templates, and Sample Forms

•Policies and Procedures on Child Assent and Parent Permission

Documents and Waivers

Permission/Assent Description	Assent or Permission Type	Assent/Permission Document

Documents and Waivers

Permission/Assent Description (e.g. Assent for Group A, Permission for Group A, Waiver of Parent Permission for Group B, Assent for Group B etc) — Assent Waiver for EE, parasites, LNS, nutrition

Assent or Permission Type — Assent Waiver

For CPHS to approve a waiver of child assent (e.g. no assent will be obtained from child/minor at all), either criterion A, B, C or D must be met. Please check the applicable criterion and provide justification in the box below.

Y **A.** The capability of some or all of the children is so limited that they cannot reasonably be consulted.

This children in this study are very young (age 0 - 30 months). Mothers/guardians of these children will be asked to consent on behalf of these children.

B. The intervention or procedure involved in the research holds out the prospect of direct benefit to the health or well-being of the children and is available only in the context of the research.

C. (1) The research involves no more than minimal risk of harm to the subjects;

(2) The waiver or alteration will not adversely affect the rights and welfare of the subjects;

(3) The research could not practicably be carried out without the waiver or alteration; and

(4) Whenever appropriate, the subjects will be provided with pertinent information after participation.

D. (1) The research or demonstration project is to be conducted by or subject to the approval of state or local officials and is designed to study, evaluate, or otherwise examine: (i) public benefit or service programs; (ii) procedures for obtaining benefits or service; (iii) possible changes in or alternatives to those programs or procedures; or (iv) possible changes in methods or levels of payment for benefits or services under those programs; AND

(2) The research could not practicably be carried out without the waiver or alteration.

---

* * * HIPAA * * *

21. Health Insurance Portability and Accountability Act (HIPAA)

The HIPAA Privacy Rule establishes the right of an individual to authorize a covered entity, such as a health plan, health care clearinghouse or health care provider, to use and disclose his/her Protected Health Information (PHI) for research purposes. UC Berkeley's covered entities are the Tang Health Center, Optometry Clinic, and Psychology Clinic. The Privacy Rule defines the elements of individual information that comprise PHI and establishes the conditions under which PHI may be used or disclosed by covered entities for research purposes. It also includes provisions to allow an individual's PHI to be disclosed or used in research without their authorization (i.e., IRB waiver of authorization). For more information, see HIPAA and Human Subjects.

a.

Does the study involve use of Protected Health Information (PHI) from a "covered entity" outside of UC Berkeley (i.e. another organization or institution)?

N

If Yes, explain what arrangements have been made to comply with the HIPAA requirements of the entity from which the PHI will be obtained:

b.

Does the study involve use of a "limited data set" from UC Berkeley's Tang Health Center, Optometry Clinic, or Psychology Clinic?

N

If Yes, patient authorization for use of the data set is not required; however, you must have a data use agreement in place with the entity from which the data will be obtained as required by HIPAA. Attach a copy of the agreement in the Attachments section.

c.

Does the study involve use of Protected Health Information (PHI) from UC Berkeley's Tang Health Center, Optometry Clinic, or Psychology Clinic?

N

Protocol Title: Measuring the benefits of sanitation, water quality, handwashing and nutrition interventions for improving health and development in rural Bangladesh

Protocol Status: APPROVED

Date Submitted: 08/18/2014

Approval Period: 09/16/2014-11/03/2016

Important Note: **This Print View may not reflect all comments and contingencies for approval. Please check the comments section of the online protocol. Questions that appear to not have been answered may not have been required for this submission. Please see the system application for more details.**

If Yes (and a limited data set will not be used), EITHER request/add a Waiver/Alteration of HIPAA Authorization below OR provide a HIPAA Authorization Form in the Attachments section of the protocol.

HIPAA WAIVER/ALTERATION: For each waiver or alteration of the requirement for authorization from the patient for use of his or her PHI, provide justification in the table below.

Note: Use table below ONLY when requesting waiver/alteration of HIPAA authorization for use of PHI from UC Berkeley's Tang Health Center, Optometry Clinic, or Psychology Clinic. For more information, see HIPAA Decision Tree for UCB PHI

Protocol Title: Measuring the benefits of sanitation, water quality, handwashing and nutrition interventions for improving health and development in rural Bangladesh

Protocol Status: APPROVED

Date Submitted: 08/18/2014

Approval Period: 09/16/2014-11/03/2016

Important Note: This Print View may not reflect all comments and contingencies for approval. Please check the comments section of the online protocol.
Questions that appear to not have been answered may not have been required for this submission. Please see the system application for more details.

* * * Attachments * * *

22. Attachments

Add appropriate attachments (e.g., advertisements, data collection instruments, IRB approvals from collaborating institutions, etc.) in this section. Attachments MUST be in PDF format.

CITI Certificate(s)

Document Type	Document Name	Attached Date	Submitted Date
CITI Certificate(s)	Jade CITI completion report_20111127	11/28/2011	01/12/2012
CITI Certificate(s)	Eugene CITI Completion Report 20130726	07/26/2013	07/28/2013
CITI Certificate(s)	BenArnold-CITI	07/28/2013	07/28/2013
CITI Certificate(s)	CitiApprovalBiomedical_Hubbard2013	07/28/2013	07/28/2013
CITI Certificate(s)	Colford CITI Completion Report	07/28/2013	07/28/2013
CITI Certificate(s)	Fernald CITI Group 1 Completion Report	07/28/2013	07/28/2013
CITI Certificate(s)	Audrie - Part 2 Citi	07/28/2013	07/28/2013
CITI Certificate(s)	Ercumen_CITI Completion_2008	11/19/2013	11/19/2013
CITI Certificate(s)	KNelson CITI Human Subjects completion report	11/19/2013	11/19/2013

Inter-institutional Agreement

Document Type	Document Name	Attached Date	Submitted Date
Inter-institutional Agreement	2011-09-3652_IIA_Wagner	07/21/2014	07/21/2014

Notice of Intent to Rely Form

Document Type	Document Name	Attached Date	Submitted Date
Notice of Intent to Rely Form	NOITR-597-0	05/30/2013	06/03/2013

Other

Protocol Title: Measuring the benefits of sanitation, water quality, handwashing and nutrition interventions for improving health and development in rural Bangladesh

Protocol Status: APPROVED

Date Submitted: 08/18/2014

Approval Period: 09/16/2014-11/03/2016

Important Note: This Print View may not reflect all comments and contingencies for approval. Please check the comments section of the online protocol.
Questions that appear to not have been answered may not have been required for this submission. Please see the system application for more details.

Document Type	Document Name	Attached Date	Submitted Date
Other	Figure 1-Study Design	03/06/2012	03/06/2012
Other	Nutrition-Supplement-Analysis	03/06/2012	03/06/2012
Other	WASH Benefits Behavior Change Strategy Overview Feb 21 2012	03/16/2012	03/16/2012

Other Institutions' IRB Approvals

Document Type	Document Name	Attached Date	Submitted Date
Other Institutions' IRB Approvals	Prot PR-11063_ERC approval_9 April 2012	04/13/2012	04/30/2012
Other Institutions' IRB Approvals	Stanford WASH Benefits IRB approval Jan 2013	07/15/2013	07/16/2013
Other Institutions' IRB Approvals	Prot PR-11063_ERC certificate on yearly review_1 Sep 2013	09/27/2013	10/03/2013
Other Institutions' IRB Approvals	Protocol PR # 11063_ERC approval_addendum_17 NOV	12/05/2013	12/05/2013
Other Institutions' IRB Approvals	Stanford WASH B Extension Dec 2013 ApprovalLetter-25863	03/26/2014	03/29/2014
Other Institutions' IRB Approvals	Prot PR-11063_ERC approval addendum_10 June 2014	08/27/2014	08/29/2014
Other Institutions' IRB Approvals	PR-11063_RRC Addendum Approval_13 April 2014	08/29/2014	08/29/2014

Recruitment Script(s)

Document Type	Document Name	Attached Date	Submitted Date
Recruitment Script(s)	WASH Benefits Bangladesh Spillover recruitment script	04/08/2014	04/08/2014

References

Document Type	Document Name	Attached Date	Submitted Date
References	References_2014_08	08/18/2014	08/18/2014

Survey Instruments

Protocol Title: Measuring the benefits of sanitation, water quality, handwashing and nutrition interventions for improving health and development in rural Bangladesh

Protocol Status: APPROVED

Date Submitted: 08/18/2014

Approval Period: **09/16/2014-11/03/2016**

Important Note: **This Print View may not reflect all comments and contingencies for approval. Please check the comments section of the online protocol. Questions that appear to not have been answered may not have been required for this submission. Please see the system application for more details.**

Document Type	Document Name	Attached Date	Submitted Date
Survey Instruments	Wash Benefits_Main Study Baseline_04_3 2012	03/06/2012	03/06/2012
Survey Instruments	Wash Benefits Bangladesh Baseline Questionnaire_2013_03	07/08/2013	07/08/2013
Survey Instruments	EE Collection Form v6_18.02.2013 (Paper)	10/03/2013	10/03/2013
Survey Instruments	Wash Benefits Midline_Endline Instruments_131003	10/03/2013	10/03/2013
Survey Instruments	wash benefits endline spillover compounds instrument_v2	02/25/2014	02/28/2014
Survey Instruments	Wash Benefits Midline & Endline Survey Questionnaires_no life experiences	09/09/2014	09/12/2014

Document Type CITI Certificate(s)
Document Name Jade CITI completion report_20111127

Document Type CITI Certificate(s)
Document Name Eugene CITI Completion Report 20130726

Document Type CITI Certificate(s)
Document Name BenArnold-CITI

Document Type CITI Certificate(s)
Document Name CitiApprovalBiomedical_Hubbard2013

Document Type CITI Certificate(s)
Document Name Colford CITI Completion Report

**Document Type**	CITI Certificate(s)
**Document Name**	Fernald CITI Group 1 Completion Report
**Document Type**	CITI Certificate(s)
**Document Name**	Audrie - Part 2 Citi
**Document Type**	CITI Certificate(s)
**Document Name**	Ercumen_CITI Completion_2008
**Document Type**	CITI Certificate(s)
**Document Name**	KNelson CITI Human Subjects completion report
**Document Type**	Inter-institutional Agreement
**Document Name**	2011-09-3652_IIA_Wagner
**Document Type**	Notice of Intent to Rely Form
**Document Name**	NOITR-597-0
**Document Type**	Other
**Document Name**	Figure 1-Study Design
**Document Type**	Other
**Document Name**	Nutrition-Supplement-Analysis
**Document Type**	Other

Protocol Title: Measuring the benefits of sanitation, water quality, handwashing and nutrition interventions for improving health and development in rural Bangladesh

Protocol Status: APPROVED

Date Submitted: 08/18/2014

Approval Period: 09/16/2014-11/03/2016

Important Note: **This Print View may not reflect all comments and contingencies for approval. Please check the comments section of the online protocol. Questions that appear to not have been answered may not have been required for this submission. Please see the system application for more details.**

**Document Name**	WASH Benefits Behavior Change Strategy Overview Feb 21 2012
**Document Type**	Other Institutions' IRB Approvals
**Document Name**	Prot PR-11063_ERC approval_9 April 2012
**Document Type**	Other Institutions' IRB Approvals
**Document Name**	Stanford WASH Benefits IRB approval Jan 2013
**Document Type**	Other Institutions' IRB Approvals
**Document Name**	Prot PR-11063_ERC certificate on yearly review_1 Sep 2013
**Document Type**	Other Institutions' IRB Approvals
**Document Name**	Protocol PR # 11063_ERC approval_addendum_17 NOV
**Document Type**	Other Institutions' IRB Approvals
**Document Name**	Stanford WASH B Extension Dec 2013 ApprovalLetter-25863
**Document Type**	Other Institutions' IRB Approvals
**Document Name**	Prot PR-11063_ERC approval addendum_10 June 2014
**Document Type**	Other Institutions' IRB Approvals
**Document Name**	PR-11063_RRC Addendum Approval_13 April 2014
**Document Type**	Recruitment Script(s)

**Protocol Title:**	Measuring the benefits of sanitation, water quality, handwashing and nutrition interventions for improving health and development in rural Bangladesh
**Protocol Status:**	APPROVED
**Date Submitted:**	08/18/2014
**Approval Period:**	**09/16/2014-11/03/2016**
**Important Note:**	**This Print View may not reflect all comments and contingencies for approval. Please check the comments section of the online protocol. Questions that appear to not have been answered may not have been required for this submission. Please see the system application for more details.**

**Document Name**	WASH Benefits Bangladesh Spillover recruitment script
**Document Type**	References
**Document Name**	References_2014_08
**Document Type**	Survey Instruments
**Document Name**	Wash Benefits_Main Study Baseline_04_3 2012
**Document Type**	Survey Instruments
**Document Name**	Wash Benefits Bangladesh Baseline Questionnaire_2013_03
**Document Type**	Survey Instruments
**Document Name**	EE Collection Form v6_18.02.2013 (Paper)
**Document Type**	Survey Instruments
**Document Name**	Wash Benefits Midline_Endline Instruments_131003
**Document Type**	Survey Instruments
**Document Name**	wash benefits endline spillover compounds instrument_v2
**Document Type**	Survey Instruments
**Document Name**	Wash Benefits Midline & Endline Survey Questionnaires_no life experiences

---

**Protocol Title:**	Measuring the benefits of sanitation, water quality, handwashing and nutrition interventions for improving health and development in rural Bangladesh
**Protocol Status:**	APPROVED
**Date Submitted:**	08/18/2014
**Approval Period:**	**09/16/2014-11/03/2016**
**Important Note:**	**This Print View may not reflect all comments and contingencies for approval. Please check the comments section of the online protocol. Questions that appear to not have been answered may not have been required for this submission. Please see the system application for more details.**

* * * Assurance * * *

Assurance

As Principal Investigator, I have ultimate responsibility for the performance of this study, the protection of the rights and welfare of the human subjects, and strict adherence by all co-investigators and research personnel to CPHS requirements, federal regulations, and state statutes for human subject's research.

I hereby assure the following:

1. The information provided in this application is accurate to the best of my knowledge.

2. All experiments and procedures involving human subjects will be performed under my supervision or that of another qualified professional listed on this protocol.

3. This protocol covers the human subjects research activities described in the grant proposal(s) supporting this research and any such activities that are not covered have been/will be covered by a CPHS approved protocol.

4. The legally effective informed consent of all human subjects or their legally authorized representative will be obtained (unless waived) using only the current, approved consent form(s).

5. If any study subject experiences an unanticipated problem involving risks to subjects or others, and/or a serious adverse event, the CPHS will be informed promptly within no more than one week (7 calendar days), and receive a written report within no more than two weeks (14 calendar days), of recognition/ notification of the event.

6. No change in the design, conduct, or key personnel of this research will be implemented without prior CPHS review and approval, unless the changes are necessary to eliminate an apparent immediate hazard to subjects. Changes made to eliminate hazards to subjects will be reported to OPHS/CPHS via the AE/UP reporting process.

7. Applications for continuation review will be submitted in a timely manner prior to the expiration date to allow sufficient time for the renewal process. I understand that if approval expires, all research activity (including data analysis) must cease until I receive notice of re-approval by the CPHS.

**Protocol Title:**	Measuring the benefits of sanitation, water quality, handwashing and nutrition interventions for improving health and development in rural Bangladesh
**Protocol Status:**	APPROVED
**Date Submitted:**	08/18/2014
**Approval Period:**	**09/16/2014-11/03/2016**
**Important Note:**	**This Print View may not reflect all comments and contingencies for approval. Please check the comments section of the online protocol. Questions that appear to not have been answered may not have been required for this submission. Please see the system application for more details.**

8. Participants' complaints or requests for information about the study will be addressed appropriately.

9. I will promptly and completely comply with a CPHS decision to suspend or withdraw its approval for the project.

10. I will submit a study closure form at the conclusion of this project.

X **I have read and agree to the above assurances.**

Protocol Title: Measuring the benefits of sanitation, water quality, handwashing and nutrition interventions for improving health and development in rural Bangladesh

Protocol Status: APPROVED

Date Submitted: 08/18/2014

Approval Period: 09/16/2014-11/03/2016

Important Note: This Print View may not reflect all comments and contingencies for approval. Please check the comments section of the online protocol.
Questions that appear to not have been answered may not have been required for this submission. Please see the system application for more details.

* * * Event History * * *

Event History

Date	Status	View Attachments	Letters
09/28/2021	AMENDMENT 20 FORM SUBMITTED	Y	
09/28/2021	AMENDMENT 20 FORM CREATED		
06/01/2021	AMENDMENT 19 FORM APPROVED	Y	Y
06/01/2021	AMENDMENT 19 FORM REVIEWER(S) ASSIGNED		
05/26/2021	AMENDMENT 19 FORM SUBMITTED (CYCLE 2)	Y	
05/13/2021	AMENDMENT 19 FORM SUBMITTED (CYCLE 1)	Y	
05/04/2021	AMENDMENT 19 FORM PANEL MANAGER REVIEW		
04/27/2021	AMENDMENT 19 FORM SUBMITTED	Y	
04/21/2021	AMENDMENT 19 FORM CREATED		
09/01/2020	AMENDMENT 18 FORM APPROVED	Y	Y
09/01/2020	AMENDMENT 18 FORM REVIEWER(S) ASSIGNED		
09/01/2020	AMENDMENT 18 FORM SUBMITTED (CYCLE 4)	Y	
09/01/2020	AMENDMENT 18 FORM SUBMITTED (CYCLE 3)	Y	
07/28/2020	AMENDMENT 18 FORM SUBMITTED (CYCLE 2)	Y	
06/23/2020	AMENDMENT 18 FORM SUBMITTED (CYCLE 1)	Y	

Protocol Title: Measuring the benefits of sanitation, water quality, handwashing and nutrition interventions for improving health and development in rural Bangladesh

Protocol Status: APPROVED

Date Submitted: 08/18/2014

Approval Period: 09/16/2014-11/03/2016

Important Note: This Print View may not reflect all comments and contingencies for approval. Please check the comments section of the online protocol. Questions that appear to not have been answered may not have been required for this submission. Please see the system application for more details.

06/15/2020	AMENDMENT 18 FORM PANEL MANAGER REVIEW		
06/02/2020	AMENDMENT 18 FORM SUBMITTED	Y	
06/02/2020	AMENDMENT 18 FORM CREATED		
10/21/2019	CONTINUING REVIEW 6 FORM APPROVED	Y	Y
10/21/2019	CONTINUING REVIEW 6 FORM REVIEWER(S) ASSIGNED		
10/21/2019	CONTINUING REVIEW 6 FORM SUBMITTED (CYCLE 2)	Y	
10/16/2019	CONTINUING REVIEW 6 FORM SUBMITTED (CYCLE 1)	Y	
10/07/2019	CONTINUING REVIEW 6 FORM PANEL MANAGER REVIEW		
09/09/2019	CONTINUING REVIEW 6 FORM SUBMITTED	Y	
09/05/2019	CONTINUING REVIEW 6 FORM CREATED		
10/13/2018	CONTINUING REVIEW 5 FORM APPROVED	Y	Y
10/03/2018	CONTINUING REVIEW 5 FORM REVIEWER(S) ASSIGNED		
10/03/2018	CONTINUING REVIEW 5 FORM SUBMITTED (CYCLE 1)	Y	
10/01/2018	CONTINUING REVIEW 5 FORM PANEL MANAGER REVIEW		
09/20/2018	CONTINUING REVIEW 5 FORM SUBMITTED	Y	
09/17/2018	CONTINUING REVIEW 5 FORM CREATED		

Protocol Title: Measuring the benefits of sanitation, water quality, handwashing and nutrition interventions for improving health and development in rural Bangladesh

Protocol Status: APPROVED

Date Submitted: 08/18/2014

Approval Period: 09/16/2014-11/03/2016

Important Note: This Print View may not reflect all comments and contingencies for approval. Please check the comments section of the online protocol.
Questions that appear to not have been answered may not have been required for this submission. Please see the system application for more details.

10/30/2017	AMENDMENT 17 FORM APPROVED	Y	Y
10/30/2017	AMENDMENT 17 FORM REVIEWER(S) ASSIGNED		
10/30/2017	AMENDMENT 17 FORM SUBMITTED (CYCLE 1)	Y	
10/27/2017	AMENDMENT 17 FORM PANEL MANAGER REVIEW		
10/26/2017	AMENDMENT 17 FORM SUBMITTED	Y	
10/26/2017	AMENDMENT 17 FORM CREATED		
10/24/2017	CONTINUING REVIEW 4 FORM APPROVED	Y	Y
10/19/2017	CONTINUING REVIEW 4 FORM REVIEWER(S) ASSIGNED		
10/19/2017	CONTINUING REVIEW 4 FORM SUBMITTED (CYCLE 1)	Y	
10/05/2017	CONTINUING REVIEW 4 FORM PANEL MANAGER REVIEW		
09/14/2017	CONTINUING REVIEW 4 FORM SUBMITTED	Y	
09/12/2017	CONTINUING REVIEW 4 FORM CREATED		
11/02/2016	CONTINUING REVIEW 3 FORM APPROVED	Y	Y
10/28/2016	CONTINUING REVIEW 3 FORM REVIEWER(S) ASSIGNED		
10/28/2016	CONTINUING REVIEW 3 FORM SUBMITTED (CYCLE 1)	Y	
10/11/2016	CONTINUING REVIEW 3 FORM PANEL MANAGER REVIEW		

Protocol Title: Measuring the benefits of sanitation, water quality, handwashing and nutrition interventions for improving health and development in rural Bangladesh

Protocol Status: APPROVED

Date Submitted: 08/18/2014

Approval Period: **09/16/2014-11/03/2016**

Important Note: **This Print View may not reflect all comments and contingencies for approval. Please check the comments section of the online protocol.
Questions that appear to not have been answered may not have been required for this submission. Please see the system application for more details.**

10/03/2016	CONTINUING REVIEW 3 FORM SUBMITTED	Y	
09/20/2016	CONTINUING REVIEW 3 FORM CREATED		
04/11/2016	AMENDMENT 16 FORM APPROVED	Y	Y
04/08/2016	AMENDMENT 16 FORM REVIEWER(S) ASSIGNED		
04/07/2016	AMENDMENT 16 FORM SUBMITTED (CYCLE 1)	Y	
04/07/2016	AMENDMENT 16 FORM SUBMITTED (CYCLE 1)	Y	
03/29/2016	AMENDMENT 16 FORM PANEL MANAGER REVIEW		
03/29/2016	AMENDMENT 16 FORM SUBMITTED	Y	
03/29/2016	AMENDMENT 16 FORM CREATED		
12/21/2015	AMENDMENT 15 FORM APPROVED	Y	Y
12/18/2015	AMENDMENT 15 FORM REVIEWER(S) ASSIGNED		
12/18/2015	AMENDMENT 15 FORM PANEL MANAGER REVIEW		
12/18/2015	AMENDMENT 15 FORM SUBMITTED	Y	
12/08/2015	AMENDMENT 15 FORM CREATED		
11/06/2015	AMENDMENT 14 FORM APPROVED	Y	Y
11/03/2015	AMENDMENT 14 FORM REVIEWER(S) ASSIGNED		
11/03/2015	AMENDMENT 14 FORM PANEL MANAGER REVIEW		

11/01/2015	AMENDMENT 14 FORM SUBMITTED	Y	
09/28/2015	AMENDMENT 14 FORM CREATED		
09/28/2015	AMENDMENT 13 FORM APPROVED	Y	Y
09/25/2015	AMENDMENT 13 FORM REVIEWER(S) ASSIGNED		
09/23/2015	AMENDMENT 13 FORM SUBMITTED (CYCLE 1)	Y	
09/08/2015	AMENDMENT 13 FORM PANEL MANAGER REVIEW		
09/01/2015	AMENDMENT 13 FORM SUBMITTED	Y	
08/25/2015	AMENDMENT 13 FORM CREATED		
12/21/2014	AMENDMENT 12 FORM APPROVED	Y	Y
12/16/2014	AMENDMENT 12 FORM REVIEWER(S) ASSIGNED		
12/15/2014	AMENDMENT 12 FORM SUBMITTED (CYCLE 2)	Y	
12/11/2014	AMENDMENT 12 FORM SUBMITTED (CYCLE 1)	Y	
11/17/2014	AMENDMENT 12 FORM PANEL MANAGER REVIEW		
11/12/2014	AMENDMENT 12 FORM SUBMITTED	Y	
11/10/2014	AMENDMENT 12 FORM CREATED		
10/16/2014	AMENDMENT 11 FORM SUBMITTED (CYCLE 2)	Y	
10/16/2014	AMENDMENT 11 FORM WITHDRAWN		
10/16/2014	AMENDMENT 11 FORM SUBMITTED (CYCLE 2)	Y	

Protocol Title: Measuring the benefits of sanitation, water quality, handwashing and nutrition interventions for improving health and development in rural Bangladesh

Protocol Status: APPROVED

Date Submitted: 08/18/2014

Approval Period: **09/16/2014-11/03/2016**

Important Note: **This Print View may not reflect all comments and contingencies for approval. Please check the comments section of the online protocol. Questions that appear to not have been answered may not have been required for this submission. Please see the system application for more details.**

10/03/2014	AMENDMENT 11 FORM TABLED		
09/26/2014	AMENDMENT 11 FORM REVIEWER(S) ASSIGNED		
09/26/2014	AMENDMENT 11 FORM SUBMITTED (CYCLE 1)	Y	
09/23/2014	AMENDMENT 11 FORM PANEL MANAGER REVIEW		
09/22/2014	AMENDMENT 11 FORM SUBMITTED	Y	
09/22/2014	AMENDMENT 11 FORM CREATED		
09/17/2014	AMENDMENT 10 FORM APPROVED	Y	Y
09/15/2014	AMENDMENT 10 FORM REVIEWER(S) ASSIGNED		
09/15/2014	AMENDMENT 10 FORM SUBMITTED (CYCLE 3)	Y	
09/12/2014	AMENDMENT 10 FORM SUBMITTED (CYCLE 2)	Y	
08/29/2014	AMENDMENT 10 FORM SUBMITTED (CYCLE 1)	Y	
08/21/2014	AMENDMENT 10 FORM PANEL MANAGER REVIEW		
08/18/2014	AMENDMENT 10 FORM SUBMITTED	Y	
08/13/2014	AMENDMENT 10 FORM CREATED		
07/21/2014	AMENDMENT 9 FORM APPROVED	Y	Y
07/21/2014	AMENDMENT 9 FORM REVIEWER(S) ASSIGNED		
07/18/2014	AMENDMENT 9 FORM PANEL MANAGER REVIEW		

Protocol Title: Measuring the benefits of sanitation, water quality, handwashing and nutrition interventions for improving health and development in rural Bangladesh

Protocol Status: APPROVED

Date Submitted: 08/18/2014

Approval Period: 09/16/2014-11/03/2016

Important Note: This Print View may not reflect all comments and contingencies for approval. Please check the comments section of the online protocol.
Questions that appear to not have been answered may not have been required for this submission. Please see the system application for more details.

07/16/2014	AMENDMENT 9 FORM SUBMITTED	Y	
07/15/2014	AMENDMENT 9 FORM CREATED		
04/11/2014	AMENDMENT 8 FORM APPROVED	Y	Y
04/09/2014	AMENDMENT 8 FORM REVIEWER(S) ASSIGNED		
04/08/2014	AMENDMENT 8 FORM SUBMITTED (CYCLE 2)	Y	
03/29/2014	AMENDMENT 8 FORM SUBMITTED (CYCLE 1)	Y	
03/03/2014	AMENDMENT 8 FORM PANEL MANAGER REVIEW		
02/28/2014	AMENDMENT 8 FORM SUBMITTED	Y	
02/25/2014	AMENDMENT 8 FORM CREATED		
01/29/2014	AMENDMENT 7 FORM APPROVED	Y	Y
01/24/2014	AMENDMENT 7 FORM REVIEWER(S) ASSIGNED		
01/23/2014	AMENDMENT 7 FORM SUBMITTED (CYCLE 3)	Y	
01/23/2014	AMENDMENT 7 FORM SUBMITTED (CYCLE 2)	Y	
01/22/2014	AMENDMENT 7 FORM SUBMITTED (CYCLE 1)	Y	
01/21/2014	AMENDMENT 7 FORM PANEL MANAGER REVIEW		
01/21/2014	AMENDMENT 7 FORM SUBMITTED	Y	
01/08/2014	AMENDMENT 7 FORM CREATED		
12/12/2013	AMENDMENT 6 FORM APPROVED	Y	Y

Protocol Title: Measuring the benefits of sanitation, water quality, handwashing and nutrition interventions for improving health and development in rural Bangladesh

Protocol Status: APPROVED

Date Submitted: 08/18/2014

Approval Period: 09/16/2014-11/03/2016

Important Note: This Print View may not reflect all comments and contingencies for approval. Please check the comments section of the online protocol.
Questions that appear to not have been answered may not have been required for this submission. Please see the system application for more details.

12/06/2013	AMENDMENT 6 FORM REVIEWER(S) ASSIGNED		
12/06/2013	AMENDMENT 6 FORM SUBMITTED (CYCLE 1)	Y	
12/05/2013	AMENDMENT 6 FORM PANEL MANAGER REVIEW		
12/05/2013	AMENDMENT 6 FORM SUBMITTED	Y	
12/05/2013	AMENDMENT 6 FORM CREATED		
12/04/2013	AMENDMENT 5 FORM APPROVED	Y	Y
12/04/2013	AMENDMENT 5 FORM REVIEWER(S) ASSIGNED		
12/04/2013	AMENDMENT 5 FORM PANEL MANAGER REVIEW		
11/26/2013	AMENDMENT 5 FORM SUBMITTED	Y	
11/26/2013	AMENDMENT 5 FORM CREATED		
11/26/2013	AMENDMENT 4 FORM APPROVED	Y	Y
11/21/2013	AMENDMENT 4 FORM REVIEWER(S) ASSIGNED		
11/20/2013	DEVIATION 2 FORM APPROVED	Y	N
11/20/2013	DEVIATION 1 FORM APPROVED	Y	N
11/19/2013	AMENDMENT 4 FORM SUBMITTED (CYCLE 2)	Y	
11/12/2013	AMENDMENT 4 FORM SUBMITTED (CYCLE 1)	Y	
11/12/2013	AMENDMENT 4 FORM PANEL MANAGER REVIEW		

Protocol Title: Measuring the benefits of sanitation, water quality, handwashing and nutrition interventions for improving health and development in rural Bangladesh

Protocol Status: APPROVED

Date Submitted: 08/18/2014

Approval Period: 09/16/2014-11/03/2016

Important Note: This Print View may not reflect all comments and contingencies for approval. Please check the comments section of the online protocol.
Questions that appear to not have been answered may not have been required for this submission. Please see the system application for more details.

11/07/2013	AMENDMENT 4 FORM SUBMITTED	Y	
11/07/2013	AMENDMENT 4 FORM CREATED		
10/29/2013	CONTINUING REVIEW 2 FORM APPROVED	Y	Y
10/28/2013	DEVIATION 1 FORM REVIEWER(S) ASSIGNED		
10/28/2013	DEVIATION 2 FORM REVIEWER(S) ASSIGNED		
10/25/2013	CONTINUING REVIEW 2 FORM REVIEWER(S) ASSIGNED		
10/25/2013	DEVIATION 2 FORM REVIEWER(S) ASSIGNED		
10/25/2013	DEVIATION 2 FORM REVIEWER(S) ASSIGNED		
10/25/2013	DEVIATION 2 FORM REVIEWER(S) ASSIGNED		
10/25/2013	DEVIATION 1 FORM REVIEWER(S) ASSIGNED		
10/25/2013	DEVIATION 1 FORM PANEL MANAGER REVIEW		
10/25/2013	DEVIATION 2 FORM PANEL MANAGER REVIEW		
10/24/2013	DEVIATION 2 FORM SUBMITTED	Y	
10/24/2013	DEVIATION 1 FORM SUBMITTED	Y	
10/24/2013	DEVIATION 2 FORM CREATED		
10/24/2013	DEVIATION 1 FORM CREATED		

Protocol Title: Measuring the benefits of sanitation, water quality, handwashing and nutrition interventions for improving health and development in rural Bangladesh

Protocol Status: APPROVED

Date Submitted: 08/18/2014

Approval Period: 09/16/2014-11/03/2016

Important Note: This Print View may not reflect all comments and contingencies for approval. Please check the comments section of the online protocol.
Questions that appear to not have been answered may not have been required for this submission. Please see the system application for more details.

10/22/2013	CONTINUING REVIEW 2 FORM SUBMITTED (CYCLE 1)	Y	
10/08/2013	CONTINUING REVIEW 2 FORM PANEL MANAGER REVIEW		
10/03/2013	CONTINUING REVIEW 2 FORM SUBMITTED	Y	
09/23/2013	CONTINUING REVIEW 2 FORM CREATED		
07/30/2013	AMENDMENT 3 FORM APPROVED	Y	Y
07/29/2013	AMENDMENT 3 FORM REVIEWER(S) ASSIGNED		
07/28/2013	AMENDMENT 3 FORM SUBMITTED (CYCLE 4)	Y	
07/25/2013	AMENDMENT 3 FORM UNDO APPROVED		
07/24/2013	AMENDMENT 3 FORM APPROVED	Y	Y
07/24/2013	AMENDMENT 3 FORM REVIEWER(S) ASSIGNED		
07/24/2013	AMENDMENT 3 FORM SUBMITTED (CYCLE 3)	Y	
07/16/2013	AMENDMENT 3 FORM SUBMITTED (CYCLE 2)	Y	
07/08/2013	AMENDMENT 3 FORM SUBMITTED (CYCLE 1)	Y	
06/12/2013	AMENDMENT 3 FORM PANEL MANAGER REVIEW		
06/03/2013	AMENDMENT 3 FORM SUBMITTED	Y	
03/20/2013	AMENDMENT 3 FORM CREATED		
11/16/2012	AMENDMENT 2 FORM APPROVED	Y	Y

Protocol Title: Measuring the benefits of sanitation, water quality, handwashing and nutrition interventions for improving health and development in rural Bangladesh

Protocol Status: APPROVED

Date Submitted: 08/18/2014

Approval Period: 09/16/2014-11/03/2016

Important Note: This Print View may not reflect all comments and contingencies for approval. Please check the comments section of the online protocol.
Questions that appear to not have been answered may not have been required for this submission. Please see the system application for more details.

11/09/2012	AMENDMENT 2 FORM REVIEWER(S) ASSIGNED		
11/09/2012	AMENDMENT 2 FORM SUBMITTED (CYCLE 1)	Y	
11/08/2012	AMENDMENT 2 FORM PANEL MANAGER REVIEW		
11/08/2012	AMENDMENT 2 FORM SUBMITTED	Y	
11/08/2012	AMENDMENT 2 FORM CREATED		
10/24/2012	CONTINUING REVIEW 1 FORM APPROVED	Y	Y
10/18/2012	CONTINUING REVIEW 1 FORM REVIEWER(S) ASSIGNED		
10/18/2012	CONTINUING REVIEW 1 FORM SUBMITTED (CYCLE 2)	Y	
10/17/2012	CONTINUING REVIEW 1 FORM SUBMITTED (CYCLE 1)	Y	
10/12/2012	CONTINUING REVIEW 1 FORM PANEL MANAGER REVIEW		
10/11/2012	CONTINUING REVIEW 1 FORM SUBMITTED	Y	
10/03/2012	CONTINUING REVIEW 1 FORM CREATED		
05/01/2012	AMENDMENT 1 FORM APPROVED	Y	Y
04/30/2012	AMENDMENT 1 FORM SUBMITTED (CYCLE 4)	Y	
04/16/2012	AMENDMENT 1 FORM REVIEWER(S) ASSIGNED		
04/06/2012	AMENDMENT 1 FORM CONDITIONAL APPROVAL		

Protocol Title: Measuring the benefits of sanitation, water quality, handwashing and nutrition interventions for improving health and development in rural Bangladesh

Protocol Status: APPROVED

Date Submitted: 08/18/2014

Approval Period: 09/16/2014-11/03/2016

Important Note: This Print View may not reflect all comments and contingencies for approval. Please check the comments section of the online protocol. Questions that appear to not have been answered may not have been required for this submission. Please see the system application for more details.

04/01/2012	AMENDMENT 1 FORM REVIEWER(S) ASSIGNED		
03/29/2012	AMENDMENT 1 FORM REVIEWER(S) ASSIGNED		
03/26/2012	AMENDMENT 1 FORM SUBMITTED (CYCLE 2)	Y	
03/19/2012	AMENDMENT 1 FORM REVIEWER(S) ASSIGNED		
03/16/2012	AMENDMENT 1 FORM SUBMITTED (CYCLE 1)	Y	
03/12/2012	AMENDMENT 1 FORM PANEL MANAGER REVIEW		
03/12/2012	AMENDMENT 1 FORM PANEL MANAGER REVIEW		
03/06/2012	AMENDMENT 1 FORM SUBMITTED	Y	
03/05/2012	AMENDMENT 1 FORM CREATED		
01/17/2012	NEW FORM APPROVED	Y	Y
01/12/2012	NEW FORM SUBMITTED (CYCLE 1)	Y	
11/04/2011	NEW FORM CONDITIONAL APPROVAL		
10/28/2011	NEW FORM REVIEWER(S) ASSIGNED		
10/04/2011	NEW FORM PANEL MANAGER REVIEW		
10/04/2011	NEW FORM PANEL ASSIGNED		
10/03/2011	NEW FORM SUBMITTED	Y	
09/29/2011	NEW FORM CREATED		
