# Supplementary material for: A cluster-randomized trial of water, sanitation, handwashing and nutritional interventions on stress and epigenetic programming
Source: Nat Commun. 2024 Apr 26;15:3572. doi: 10.1038/s41467-024-47896-z (PMC11053067; doi:10.1038/s41467-024-47896-z)
Supplement: Supplementary file 2 — Reporting Summary [file 41467_2024_47896_MOESM2_ESM.pdf]

nature portfolio

Corresponding author(s): Audrie Lin

Last updated by author(s): 03/07/2024

Reporting Summary

Nature Portfolio wishes to improve the reproducibility of the work that we publish. This form provides structure for consistency and transparency in reporting. For further information on Nature Portfolio policies, see our Editorial Policies and the Editorial Policy Checklist.

Statistics

For all statistical analyses, confirm that the following items are present in the figure legend, table legend, main text, or Methods section.

n/a	Confirmed	
☐	☒	The exact sample size (*n*) for each experimental group/condition, given as a discrete number and unit of measurement
☐	☒	A statement on whether measurements were taken from distinct samples or whether the same sample was measured repeatedly
☐	☒	The statistical test(s) used AND whether they are one- or two-sided<br>*Only common tests should be described solely by name; describe more complex techniques in the Methods section.*
☐	☒	A description of all covariates tested
☐	☒	A description of any assumptions or corrections, such as tests of normality and adjustment for multiple comparisons
☐	☒	A full description of the statistical parameters including central tendency (e.g. means) or other basic estimates (e.g. regression coefficient) AND variation (e.g. standard deviation) or associated estimates of uncertainty (e.g. confidence intervals)
☐	☒	For null hypothesis testing, the test statistic (e.g. *F*, *t*, *r*) with confidence intervals, effect sizes, degrees of freedom and *P* value noted<br>*Give P values as exact values whenever suitable.*
☒	☐	For Bayesian analysis, information on the choice of priors and Markov chain Monte Carlo settings
☒	☐	For hierarchical and complex designs, identification of the appropriate level for tests and full reporting of outcomes
☐	☒	Estimates of effect sizes (e.g. Cohen's *d*, Pearson's *r*), indicating how they were calculated

Our web collection on statistics for biologists contains articles on many of the points above.

Software and code

Policy information about availability of computer code

Data collection	For data management, we used STATA version 14.2.
Data analysis	Analyses were conducted using R statistical software version 3.6.1. The code and replication files for the study are publicly available on Open Science Framework (https://osf.io/9573v/) and GitHub (https://github.com/washb-eed-substudies/wash-stress).

For manuscripts utilizing custom algorithms or software that are central to the research but not yet described in published literature, software must be made available to editors and reviewers. We strongly encourage code deposition in a community repository (e.g. GitHub). See the Nature Portfolio guidelines for submitting code & software for further information.

Data

Policy information about availability of data

All manuscripts must include a data availability statement. This statement should provide the following information, where applicable:

- Accession codes, unique identifiers, or web links for publicly available datasets
- A description of any restrictions on data availability
- For clinical datasets or third party data, please ensure that the statement adheres to our policy

Data availability: The prespecified, registered statistical analysis plan and deidentified individual participant data generated in this study have been deposited in Open Science Framework (https://osf.io/9573v/). Source data are provided with this paper. The raw DNA sequencing data discussed in this publication have been deposited in NCBI's Gene Expression Omnibus and are accessible through GEO Series accession number GSE261098 (https://www.ncbi.nlm.nih.gov/geo/query/acc.cgi?acc= GSE261098). The raw liquid chromatography-tandem mass spectroscopy data have been deposited in the re3data repository and are accessible

(https://doi.org/10.7924//r49311p2m). The consort checklist for the study is included in the Supplementary Information.

Research involving human participants, their data, or biological material

Policy information about studies with human participants or human data. See also policy information about sex, gender (identity/presentation), and sexual orientation and race, ethnicity and racism.

Reporting on sex and gender	The study findings apply to both sexes. Balance in the sex variable was considered in the study design. Sex was determined based on caregiver assignment because the study team administered survey questions to caregivers. A variable for sex is provided in the Source Data files. Informed consent was obtained to share deidentified individual-level data.<br><br>Stress outcomes were assessed in 688 children (51% female) at age 14.3 (IQR, 12.7–15.6) months, and 760 children (52% female) at age 28.2 (IQR, 27.0–29.6) months (Fig. 1). We conducted a pre-specified analysis estimating interactions between child sex and the intervention since biological differences, differential care practices, or other behavioral practices may influence the effect of the N+WSH interventions. The results of this analysis are presented in the Results section of the manuscript and in Supplementary Tables 4 and 5.
Reporting on race, ethnicity, or other socially relevant groupings	This study did not report on race and ethnicity.
Population characteristics	We enrolled pregnant women (mean age —24 y) in their first two trimesters and their in utero children. Table 1 is a list of enrollment characteristics by intervention group and year of measurement.
Recruitment	The main organizational unit for households in rural Bangladesh is the compound, where patrilineal families share a common courtyard. The research team visited compounds in candidate communities. If compound residents reported no iron taste in their drinking water nor iron staining of their water storage vessels, and if a woman reported being in the first two trimesters of pregnancy, the household was recruited to be in the study. The team created maps of households based on their global positioning system coordinates and created clusters of eight expectant women who lived close together, whereby a single community health promoter could walk to each compound. Trained data collectors traveled to eligible communities and asked community leaders for permission to conduct research within their community. If the community leaders agreed, then the team proceeded with recruitment. Data collectors approached randomly selected households within a community. The data collector discussed the prospect for participation in the study with the adults in the household and the pregnant woman. After providing time for discussion among the household residents, a member of the data collection team returned to the household to seek formal informed consent from the head of the household and the mother/caregiver of the target infant.<br><br>Although selection bias in an experimental study is possible due to differential losses to follow-up, this type of bias was minimized because there was <10% loss to follow-up in the overall trial. Randomization was maintained as evidenced by the balance in household enrollment characteristics between the intervention and control arms (Table 1). The enrollment characteristics of this study were similar to those from the overall trial (Supplementary Table 1). Household enrollment characteristics were balanced between individuals who had stress outcome measurements at year one versus those who were lost to follow up at year two (Supplementary Table 1).
Ethics oversight	Participants provided written informed consent. Human subjects committees at International Centre for Diarrhoeal Disease Research, Bangladesh (PR-11063 and PR-14108), the University of California, Berkeley (2011-09-3652 and 2014-07-6561) and Stanford University (25863 and 35583) approved study protocols. A data safety monitoring committee convened by icddr,b oversaw the study.

Note that full information on the approval of the study protocol must also be provided in the manuscript.

Field-specific reporting

Please select the one below that is the best fit for your research. If you are not sure, read the appropriate sections before making your selection.

[x] Life sciences   [ ] Behavioural & social sciences   [ ] Ecological, evolutionary & environmental sciences

For a reference copy of the document with all sections, see nature.com/documents/nr-reporting-summary-flat.pdf

Life sciences study design

All studies must disclose on these points even when the disclosure is negative.

Sample size	The sample size was outlined in the original trial (https://www.thelancet.com/journals/langlo/article/PIIS2214-109X(17)30490-4/fulltext). Assuming a two-sided Type-I error of 5%, 135 clusters (five children measured per cluster), and a range of cluster-level intra-class correlations for repeated measures (0.01 to 0.20), the study had 90% power to detect a difference of 333.22-438.38 U/ml/min salivary alpha-amylase, 0.26-0.34 µg/dl cortisol, 0.06-3.27 ng/mg creatinine F2-isoprostanes isomers, 1.15-1.51% NR3C1 methylation status between the combined nutrition, water, sanitation, and handwashing arm and the control arm.
Data exclusions	All inclusion and exclusion criteria were pre-established. Households with low iron and arsenic levels in drinking water, plans to live in the

	study village for the next two years, and absence of major water, sanitation, or nutrition programs were eligible for inclusion.
Replication	Four investigators (Audrie Lin, Andrew Mertens, Sophia Tan, and Lisa Kim) conducted independent masked statistical analyses following the pre-registered analysis protocol (https://osf.io/4mx7s/). The analyses were replicated once. After replication of all masked analyses, results were unmasked. All attempts at replication were successful.
Randomization	Clusters consisted of eight neighboring households with eligible pregnant women. To prevent spillover between clusters, a one km buffer around each cluster was created. Eight geographically-adjacent clusters formed a block. An investigator at UC Berkeley (Benjamin F. Arnold) used a random number generator to block randomize clusters to the non-intervention control arm or one of the six intervention arms [water; sanitation; handwashing; water, sanitation, and handwashing (WSH); nutrition; nutrition, water, sanitation, and handwashing (N+WSH)]. This study assessed children only in the control and the N+WSH arm.
Blinding	Participants and outcome assessors were not masked because interventions delivered had visible hardware (e.g., latrines, potties, etc.). Laboratory investigators were masked to group assignments. Four investigators (Audrie Lin, Andrew Mertens, Sophia Tan, and Lisa Kim) conducted independent masked statistical analyses following the pre-registered analysis protocol (https://osf.io/4mx7s/). After replication of all masked analyses, results were unmasked.

Reporting for specific materials, systems and methods

We require information from authors about some types of materials, experimental systems and methods used in many studies. Here, indicate whether each material, system or method listed is relevant to your study. If you are not sure if a list item applies to your research, read the appropriate section before selecting a response.

Materials & experimental systems

n/a	Involved in the study
☒	☐ Antibodies
☒	☐ Eukaryotic cell lines
☒	☐ Palaeontology and archaeology
☒	☐ Animals and other organisms
☐	☒ Clinical data
☒	☐ Dual use research of concern
☒	☐ Plants

Methods

n/a	Involved in the study
☒	☐ ChIP-seq
☒	☐ Flow cytometry
☒	☐ MRI-based neuroimaging

Clinical data

Policy information about clinical studies

All manuscripts should comply with the ICMJEguidelines for publication of clinical research and a completedCONSORT checklist must be included with all submissions.

Clinical trial registration	The trial was registered at ClinicalTrials.gov (NCT01590095).
Study protocol	The full study protocol can be accessed at BMJ Open: https://bmjopen.bmj.com/content/3/8/e003476
Data collection	The cluster-randomized WASH Benefits trial was conducted in rural villages in the Gazipur, Mymensingh, Tangail and Kishoreganj districts of Bangladesh. Between 31 May 2012 and 7 July 2013, 5551 women were enrolled in the study and randomly allocated to one of the intervention or control arms (Fig. 1). This study assessed stress outcomes in saliva and urine specimens collected at Year 1 and Year 2 post-enrollment.
Outcomes	The prespecified stress outcomes included physiological stress response (salivary alpha-amylase and cortisol), oxidative stress (F2-isoprostanes), and DNA methylation of the glucocorticoid receptor (NR3C1) exon 1F promoter including the NGFI-A binding site. The stress outcomes were measured as part of the original trial because they were prospectively considered and planned for in the protocol and funded by a supplemental grant from the Bill and Melinda Gates Foundation to further elucidate the mechanism of the interventions. The stress measurements were prespecified (please see pages 46-48 of the IRB protocol and IRB approval from September 17, 2014 attached) before the primary outcomes of the trial were known (trial unblinding occurred on April 6, 2016). The study protocol is available as a Supplementary Note.

Plants

Seed stocks	*Report on the source of all seed stocks or other plant material used. If applicable, state the seed stock centre and catalogue number. If plant specimens were collected from the field, describe the collection location, date and sampling procedures.*
Novel plant genotypes	*Describe the methods by which all novel plant genotypes were produced. This includes those generated by transgenic approaches, gene editing, chemical/radiation-based mutagenesis and hybridization. For transgenic lines, describe the transformation method, the number of independent lines analyzed and the generation upon which experiments were performed. For gene-edited lines, describe the editor used, the endogenous sequence targeted for editing, the targeting guide RNA sequence (if applicable) and how the editor was applied.*
Authentication	*Describe any authentication procedures for each seed stock used or novel genotype generated. Describe any experiments used to assess the effect of a mutation and, where applicable, how potential secondary effects (e.g. second site T-DNA insertions, mosiacism, off-target gene editing) were examined.*
