# Supplementary material for: A cluster-randomized trial of water, sanitation, handwashing and nutritional interventions on stress and epigenetic programming
Source: Nat Commun. 2024 Apr 26;15:3572. doi: 10.1038/s41467-024-47896-z (PMC11053067; doi:10.1038/s41467-024-47896-z)
Supplement: Supplementary file 4 — Description of Additional Supplementary Files [file 41467_2024_47896_MOESM4_ESM.pdf]

Supplementary Data 1.
Individual- and household-level dataset used in the unadjusted and adjusted analyses.

Supplementary Data 2.
Individual- and household-level dataset used in the inverse probability of censoring weighted (IPCW) adjusted analyses.
